# Insulin-degrading enzyme confers neuroprotection in Parkinson’s disease by inhibiting the Hippo signaling pathway

**DOI:** 10.1038/s41419-025-08055-4

**Published:** 2025-10-24

**Authors:** Huimin Zheng, Yu Guo, Shuyu Zhang, Yun Su, Xin Cui, Zhengwei Hu, Xiaoyan Hao, Mengjie Li, Changhe Shi, Yuming Xu, Chengyuan Mao

**Affiliations:** 1 Department of Neurology, The First Affiliated Hospital of Zhengzhou University, Zhengzhou University, Zhengzhou, 450000 Henan China; 2 The Academy of Medical Sciences of Zhengzhou University, Zhengzhou University, Zhengzhou, 450000 Henan China; 3 Henan Key Laboratory of Cerebrovascular Diseases, The First Affiliated Hospital of Zhengzhou University, Zhengzhou University, Zhengzhou, 450000 Henan China; 4 NHC Key Laboratory of Prevention and treatment of Cerebrovascular Diseases, Zhengzhou, 450052 Henan China; 5 Department of Neurosurgery, The First Affiliated Hospital of Zhengzhou University, Zhengzhou University, Zhengzhou, 450000 Henan China

**Keywords:** Cell death in the nervous system, Parkinson's disease

## Abstract

Parkinson’s disease (PD) is a progressive neurodegenerative disorder primarily marked by the degeneration of dopaminergic neurons and pathological α-synuclein (α-syn) accumulation. Although insulin-degrading enzyme (IDE) has been implicated in both type 2 diabetes mellitus and amyloid-protein clearance, its precise relevance to PD pathogenesis remains unclear. In this study, we show that IDE expression is reduced in the nigrostriatal region of aging homozygous A53T α-syn mice and in α-syn-overexpressing SH-SY5Y PD cells. Overexpression of IDE alleviated motor deficits, reduced pathological α-syn levels, and protected dopaminergic neurons in A53T α-syn mice. In SH-SY5Y PD model cells, IDE overexpression reduced α-syn-induced toxicity, whereas IDE knockdown exacerbated it. Integrated transcriptomic and proteomic analyses revealed that the Hippo signaling pathway serves as a major downstream target of IDE. Notably, inhibition of MST1/2, a pivotal Hippo kinase, recapitulated IDE’s neuroprotective effects by diminishing α-syn pathology and neuronal apoptosis. Hence, IDE confers neuroprotection partly via suppression of the Hippo signaling pathway, and pharmacological targeting of the IDE-Hippo axis may represent a promising therapeutic strategy for PD.

## Introduction

Parkinson’s disease (PD) is a progressive neurodegenerative disorder primarily marked by the degeneration of dopaminergic neurons in the substantia nigra pars compacta, leading to striatal dopamine deficiency and the accumulation of α-synuclein (α-syn) inclusions [1, 2]. Clinically, PD is characterized by motor symptoms (tremor, bradykinesia, rigidity, and postural instability) and multiple non-motor symptoms (cognitive decline, depression, and autonomic dysfunction) [3]. Despite multiple pathogenic factors—such as *SNCA* or *LRRK2* mutations, environmental exposures, and aging—a unifying mechanism remains elusive [4].

In parallel, type 2 diabetes mellitus (T2DM) has been recognized as a notable risk factor for PD, with epidemiological data indicating that insulin resistance and islet amyloid polypeptide (IAPP) deposition may trigger neurodegenerative processes [5, 6]. Clinical and preclinical studies indicate that T2DM treatments (e.g., GLP-1 receptor agonists) can ameliorate motor and cognitive symptoms in PD, underscoring the notion that overlapping protein misfolding and metabolic dysregulation may drive both diseases [7, 8]. Insulin-degrading enzyme (IDE) is a zinc metalloprotease known primarily for cleaving insulin, yet it also degrades or interacts with multiple amyloidogenic proteins, including amyloid-β (Aβ) and α-syn [9, 10]. Previous studies show that IDE binds the C-terminus of α-syn, preventing its fibrillization via a non-proteolytic mechanism [11, 12]. IDE deficiency or dysfunction has been tied to the pathogenesis of T2DM and Alzheimer’s disease (AD) [13, 14]. However, contradictory proteomic data in PD patients and limited understanding of IDE’s role in α-syn aggregation underscore the need for further investigation [15].

Here, we asked whether loss of IDE accelerates α-synuclein pathology and whether IDE restoration can halt disease progression. Longitudinal profiling in A53T α-syn mice and α-syn-overexpressing SH-SY5Y cells revealed a pronounced decline of IDE in the nigrostriatal system. Bidirectional IDE manipulation confirmed that reinstating IDE improves motor function, protects dopaminergic neurons, and reduces α-syn aggregates. Multi-omics analysis identified suppression of the Hippo kinase module as the dominant downstream signature of IDE; pharmacological inhibition of its core kinases MST1/2 further potentiated IDE-mediated protection. These results delineate an IDE–Hippo axis that mitigates α-syn toxicity and nominate IDE augmentation or Hippo inhibition as practical, mechanism-based strategies for Parkinson’s disease.

## Materials and methods

### Animals and treatment

All animal studies and experimental procedures were approved by the Life Science Ethics Review Committee of Zhengzhou University. Homozygous A53T α-syn transgenic M83+/+ mice [B6; C3-Tg (Prnp-SNCA*A53T) 83Vle/J] and the age-matched littermate wild-type (WT) male mice were purchased from the Cavens Laboratory Animal Center (Cavens, Jiangsu, China). Mice were housed under standard 12-h light/dark cycles with free access to food and water.

Adeno-associated virus (AAV) vector (AAV.CAP-b10-IDE-3xFLAG and AAV.CAP-b10-3xFLAG) was constructed in OBiO Technology Corp., Ltd. (Shanghai, China). 200 μl AAV at a final titer of 2 × 10^12^ vg/mL was injected into the tail vein. 6-month-old WT and A53T α-syn mice were randomly selected for three groups: (1) WT + Vector (AAV.CAP-b10-3xFLAG for WT mice, *n* = 12), (2) A53T+Vector (AAV.CAP-b10-3xFLAG for A53T α-syn mice, *n* = 12), and (3) A53T + IDE (AAV.CAP-b10-IDE-3xFLAG for A53T α-syn mice, *n* = 12). Subsequent experiments were performed at 180 days post injection (*dpi*).

### ELISA

Tail vein blood of fasting mice was collected and centrifuged for 15 min at 3000 rpm and 4 °C to extract serum. The harvested supernatant was stored at −80 °C until use. Serum IDE levels were measured using an ELISA kit (Cloud-Clone, Houston, TX, USA) according to the manufacturer’s protocol.

### IDE activity assays

Brain tissues were collected and homogenized in the assay buffer of the SensoLyte® 520 IDE Activity Assay Kit (AnaSpec, Fremont, US) on ice. The results of preliminary experiments determined the appropriate protein concentration (500 μg/ml) of each tissue homogenate in subsequent assays. The protein concentration of samples was determined and suitably diluted to 500 μg/ml using the BCA kit (Solarbio, Beijing, China). The assay utilizes a FRET substrate that emits enhanced fluorescence upon cleavage by IDE. The fluorescence intensity and IDE activity were measured using a BioTek® Synergy HTX (Agilent, Santa Clara, US), complying with the manufacturer’s protocol.

### Pole test

The pole test was implemented to detect locomotor coordination of mice. Before the test, mice acclimated to the pole (60 cm in length, 10 mm in diameter) to receive 3 training trials per day for 3 consecutive days. The time to descend the pole was obtained.

### Rotarod test

The rotarod test was conducted to assess balance and locomotor coordination in mice. The day before the test, mice were pretrained for 3 consecutive days on an accelerated rotating rod from 4 rpm/min to 40 rpm/min within 5 min. Each mouse was tested two times, resting for 30 min. In the testing for day 4, the same procedure was performed, and the latency to fall (in seconds) was recorded.

### Open field test

The open field (OF) test was used to test locomotor activity and anxiety-like behaviors in mice. The mice were given 30 min beforehand to acclimate to the environment of the testing room. Each mouse was placed in the center of an open field box (50 cm × 50 cm × 50 cm) with the video recorder above and allowed to freely explore for 5 min. The box was routinely cleaned with 75% ethanol following each test. The final video was assessed for the total distance travelled and the time spent in the center via *Open Field software* (Xinruan Information Technology Co., Ltd., Shanghai, China).

### Elevated plus maze test

The elevated plus maze (EPM) test was performed to evaluate the anxiety-like behaviors of mice. The mice were given 30 minutes to adapt to the environment before the formal test. Then each mouse was placed in the middle of the junction area (5 cm × 5 cm, facing the open arm) between the open arm (30 cm × 5 cm) and closed arm (30 cm × 5 cm) with the camera above and allowed 5 min of free exploration. The maze was cleaned with 75% ethanol between each trial. The recorded video was analyzed for the percentage of time spent in the open arm via *Elevated Plus Maze software* (Xinruan Information Technology Co., Ltd., Shanghai, China).

### Protein extraction and immunoblotting analysis

Brain tissues and cultured cells were lysed and homogenized with ice-cold RIPA buffer containing 1X PMSF, 1X protease inhibitor, and 1X phosphatase inhibitor (Solarbio, Beijing, China) in the high-speed, low-temperature homogenizer (Servicebio, Wuhan, China). After the execution of the grinding program, grinding tubes containing the homogenates and grinding beads were put on the ice, standing for 30 min. Grinding tubes were centrifuged to collect the supernatants. Some supernatants of samples were measured for protein concentration by the BCA kit (Solarbio, Beijing, China). Samples were suitably diluted and denatured with lysis buffer and SDS-PAGE loading buffer (Solarbio, Beijing, China) to ensure uniform total protein concentration. Harvested samples were separated via SDS-PAGE (Beyotime, Shanghai, China) and blotted onto PVDF membranes (Merck Millipore, Darmstadt, Germany). The membranes were blocked with Quick-Blocking buffer (Beyotime, Shanghai, China) at room temperature (RT) for 45 min, followed by incubation with primary antibodies overnight at 4 °C (refer to Supplementary Table S1) and HRP-linked secondary antibodies at RT for 90 min. Finally, protein bands were visualized via an ECL kit (Genview, Beijing, China), and signals were captured and measured by using the Amersham Imager 680 (Cytiva, Danaher, US) and ImageJ software.

### RNA isolation and real-time quantitative PCR

Total RNA was extracted from brain tissues and cultured cells using the FastPure® Cell/Tissue Total RNA Isolation Kit (Vazyme, Nanjing, China), according to the manufacturer’s instructions. cDNA synthesis was performed with a HiScript® III All-in-one RT SuperMix Perfect for qPCR Kit (Vazyme, Nanjing, China). Real-time quantitative PCR was conducted using the Taq Pro Universal SYBR qPCR Master Mix (Vazyme, Nanjing, China) and the QuantStudio 5 PCR system (Thermo Fisher Scientific, MA, USA). The following thermal cycling parameters complied with the manufacturer’s instructions. Notably, triplicate samples were applied in each independent experiment to further data analyses. The primers for qRT-PCR are summarized in Supplementary Table S2.

### Histology and immunohistochemistry

After the last behavioral test, mice were deeply anesthetized by inhalation of 2–3% isoflurane (RWD, Shenzhen, China) and sequentially perfused with PBS and 4% paraformaldehyde (Servicebio, Wuhan, China) by micro-pumping instrument (Longerpump, Baoding, China). Then brains were separated and fixed in 4% paraformaldehyde overnight and dehydrated in sucrose solution (Biosharp, Hefei, China). Brain tissues were then embedded in OCT (Lecia, Wetzlar, Germany) and cut into a thickness of 10 μm coronal frozen sections using the Lecia frozen sectioning machine (Lecia, Wetzlar, Germany). The slices were stored at −20 °C for further experiments.

For immunohistochemical staining, the IHC Kit (Zsbio, Beijing, China) was employed, involving 3,3-diaminobenzidine-tetrachloride (DAB) staining, hematoxylin counterstaining, and subsequent dehydration and clearing. Sections were rinsed three times in PBS and performed antigen retrieval in sodium citrate solution (Solarbio, Beijing, China). Endogenous peroxidase activity was inactivated, and sections were incubated overnight at 4 °C with primary antibodies (refer to Supplementary Table S1). The slices were stained with DAB solution to detect protein expression. After the hematoxylin counterstaining, the sections were dehydrated, permeabilized, and sealed to observe under the Pannoramic MIDI digital slicing scanner (3DHISTECH, Budapest, Hungary). The results of immunoreactivity were measured using ImageJ software.

### Immunofluorescence

For immunofluorescence, frozen tissue sections (performed the same antigen retrieval process) or fixed-adherent cells were permeabilized with 0.3% Triton X-100 (Solarbio, Beijing, China), blocked with 5% bovine serum albumin (BSA; Biotepped, Beijing, China), and then incubated with primary antibodies overnight at 4 °C (refer to Supplementary Table S1). After that, fluorescent-labeled secondary antibodies (refer to Supplementary Table S1) were incubated, and the nuclei were counterstained with DAPI (SouthernBiotech, Birmingham, USA) for visualization. The image acquisition of sealed slides was performed with the Pannoramic MIDI digital slicing scanner (3DHISTECH, Budapest, Hungary) and analyzed using ImageJ software.

### Cell culture and treatment

Human neuroblastoma cells (SH-SY5Y) were procured from Wuhan Procell Life Science and Technology (Wuhan, China) and maintained in MEM/F12 (Procell, Wuhan, China) with 15% FBS (ExCell Bio, Suzhou, China). The Human Embryonic Kidney 293T (HEK-293T) cell line used in this study was a gift from Henan Key Laboratory of Cerebrovascular Diseases (Zhengzhou, China) and cultured in DMEM (Corning, NY, USA) with 10% FBS. Cells were incubated at 37 °C in a 5% CO_2_ humidified atmosphere.

The Flag-IDE and Myc-α-syn plasmids were obtained from Shanghai Genechem Co., Ltd. (Genechem, Shanghai, China) to implement the coimmunoprecipitation (CO-IP) experiment. When HEK-293T cells reach 70–80% confluence, they are transfected with plasmids using Lipofectamine 3000 (Thermo Fisher Scientific, MA, USA) according to the manufacturer’s instructions.

Lentiviral vectors (LV) were obtained from Shanghai Genechem Co., Ltd. (Genechem, Shanghai, China), which include empty vector (EV), α-syn-WT (SNCA^WT^), α-syn-A53T (SNCA^A53T^), LV-Con and LV-IDE. LV for EV, SNCA^WT^, SNCA^A53T^ were cloned into a Flag-EGFP tag, and LV-Con and LV-IDE were cloned into a Myc-Cherry tag. SH-SY5Y cells were infected with an appropriate MOI and selected with puromycin or G418 according to the manufacturer’s instructions. Then constructed, stably overexpressed SH-SY5Y cell lines were used for subsequent experiments.

Small interfering RNA (siRNA) targeting the IDE sequence (IDE-siRNA) and negative control (NC) were designed and synthesized by OBiO Technology Corp., Ltd. (Shanghai, China). The related sequences were listed in Supplementary Table S2. IDE-siRNA or NC was transfected into SH-SY5Y cells using RNAiMAX (Thermo Fisher Scientific, MA, USA) according to the manufacturer’s instructions.

XMU-MP-1 was purchased (MCE, New Jersey, USA) to pharmacologically inhibit the MST1/2 targeting on the Hippo signaling pathway. According to the previous study, SH-SY5Y cells were treated with 5 μM XMU-MP-1 dissolved in DMSO for 24 h [16].

All in all, after infection and transfection, visualizing GFP or m-Cherry expression and collecting the extracted RNA and protein from cellular samples for RT-qPCR and Western blot analysis were used to verify the efficiency of experiments. Then constructed cells were collected for subsequent experiments.

### Cell viability and CCK8 assay

The Cell Counting Kit-8 (CCK-8) method was used to determine the changes in cell viability (Dojindo, Kumamoto, Japan). Briefly, 100 μL cell suspensions were inoculated into 96-well plates (1 × 10^5^ cells/well) and cultured for 24 h. Then, 10 μL of CCK-8 reagent was added and incubated for 3 h. The absorbance values were read at 450 nm using an automatic microplate spectrophotometer (Liuyi, Beijing, China).

### TUNEL staining

Terminal deoxynucleotidyl transferase dUTP nick end labeling (TUNEL) staining was performed to detect cell death with DNA fragmentation (Vazyme, Nanjing, China). Frozen tissue slides or adherent cells were fixed with 4% paraformaldehyde (Servicebio, Wuhan, China) and permeabilized with 0.3% Triton X-100 (Solarbio, Beijing, China). Afterward, slides or adherent cells were incubated in the TUNEL reaction mixture in a wet box at 37 °C, and nuclei were counterstained with DAPI (SouthernBiotech, Birmingham, USA). Images were observed by the Pannoramic MIDI digital slicing scanner (3DHISTECH, Budapest, Hungary) and analyzed using ImageJ software.

### Proteomic analysis

Protein extraction and BCA assay were implemented to calculate the protein concentration of the brain tissues. For each sample, 500 ng of lysed peptides were harvested and analyzed with a nano­UPLC system coupled to an Astral instrument (Thermo Fisher Scientific, MA, USA) with a nano­electrospray ion source. The separation of peptides was performed by using a reversed-phase column (EASY-Spray™ HPLC, Thermo Fisher Scientific, MA, USA). Separation of the sample was executed with a 6.9 min gradient. Data-independent acquisition (DIA) was acquired in profile and positive mode with the Orbitrap analyzer. Notably, differentially expressed proteins (DEPs) were considered in our differential expression if they exhibited average fold change (FC) values greater than 1.2 with a *p*-value less than 0.05 or FC values less than 0.83 with a *p*-value less than 0.05. Data analyses were performed using the UniProt Mus musculus database.

### RNA-seq data analysis

RNA from brain tissues was isolated and purified using TRIzol (Thermo Fisher Scientific, MA, USA) according to the manufacturer’s instructions. The quantity and purity of total RNA were assessed using NanoDrop (Thermo Fisher Scientific, MA, USA), and RNA integrity was analyzed with a Bioanalyzer 2100 (Agilent, CA, USA). The RNA concentration was required to be >50 ng/μL, the RIN value >7.0, and the total RNA >1 μg to meet the requirements for downstream experiments. mRNA with poly(A) tails was specifically captured using oligo(dT) magnetic beads (Thermo Fisher Scientific, MA, USA) through two rounds of purification. The captured mRNA was fragmented using a Magnesium RNA Fragmentation kit (New England Biolabs, MA, USA). Then fragmented RNA was reverse-transcribed into cDNA, and double-stranded cDNA was connected to an adapter. Finally, purification and fragment sorting were performed, and the final gene libraries obtained by PCR amplification were subjected to paired-end sequencing (PE150) on the Illumina NovaSeq™ 6000 platform following standard procedures. Specifically, only those differentially expressed genes (DEGs) meeting an average normalized expression value ≥1 with a *p*-value less than 0.05 were included in subsequent bioinformatics analysis.

### Statistical analyses

The threshold for statistical significance was set at *P* < 0.05 (two-tailed). Data were analyzed with SPSS software (version 21.0), and graphs were created by GraphPad Prism software (version 9.0.0). Additionally, comparisons between the two groups were analyzed using the Mann–Whitney U test or unpaired Student’s *t* test, depending on whether the variables had non-normal or normal distributions. Three-group comparisons were performed by using one-way ANOVA (parametric distribution) or the Kruskal–Wallis test (non-parametric distribution). For pairwise comparisons among three groups, Tukey’s multiple comparisons test or Dunn’s multiple comparisons test was utilized for variables with parametric or non-parametric distribution, respectively.

## Results

### Reduced IDE in aging A53T α-syn transgenic PD mice

**Fig. 1:**
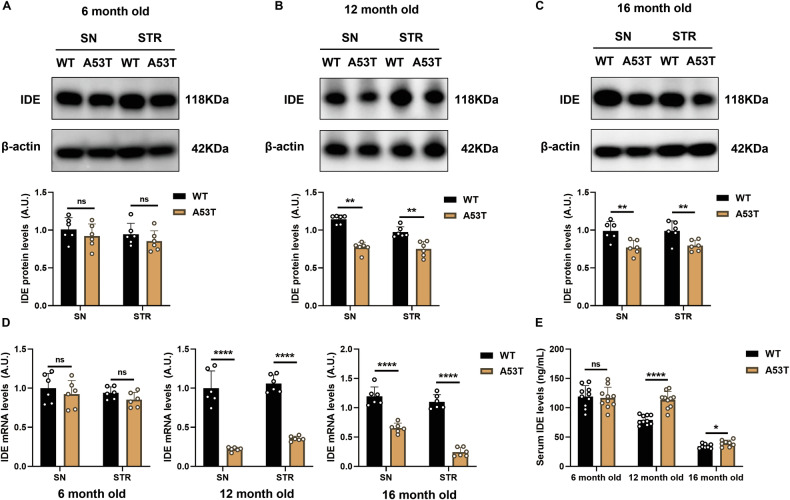
Decreased nigrostriatal IDE levels and elevated serum IDE levels in A53T α-syn mice. **A**–**C** IDE protein levels were measured in SN and STR of 6, 12, 16-month-old A53T α-syn mice (*n* = 6) and WT control mice (*n* = 6). Quantified results of the expression of IDE were also displayed. **P < 0.01, ns not significant; Student’s *t* test. **D** Quantitative transcription analyses exhibiting the expression levels of IDE mRNA in 6, 12, 16-month-old A53T α-syn mice (*n* = 6) and WT control mice (*n* = 6). ****P < 0.0001, ns not significant; Student’s *t* test. **E** The total fasting serum IDE levels of 6, 12, 16-month-old A53T α-syn mice (*n* = 8-10) and WT control mice (*n* = 8–10) were measured in ELISA kits. ****P < 0.0001, *P < 0.05, ns not significant; Student’s *t* test.

Pronounced dopaminergic degeneration—evidenced by reduced tyrosine hydroxylase (TH)—emerged in 12- and 16-month-old, but not 6-month-old, A53T α-syn mice (Supplementary Fig. S1A–C). To determine whether IDE is involved in this progression, we assessed its expression and activity across ages. At the protein and mRNA level, IDE in substantia nigra (SN) and striatum (STR) was unchanged at 6 months but declined sharply at 12 and 16 months (Fig. 1A–D), whereas cerebral cortex (CC) and hippocampus (HP) remained unaffected (Supplementary Fig. S1D–G). Despite preserved expression in CC and HP, IDE catalytic activity was significantly reduced across all four regions (SN, STR, CC, HP) in A53T α-syn mice (Supplementary Fig. S1H). In contrast, fasting serum IDE rose at 12 and 16 months (Fig. 1E). Together, these data indicate a regionally selective loss of IDE expression within the nigrostriatal system accompanied by a broader brain-wide reduction in IDE activity, coincident with the onset of TH depletion and α-syn accumulation; the peripheral increase may reflect a compensatory redistribution.

### Reduced IDE in SH-SY5Y PD model cells

**Fig. 2:**
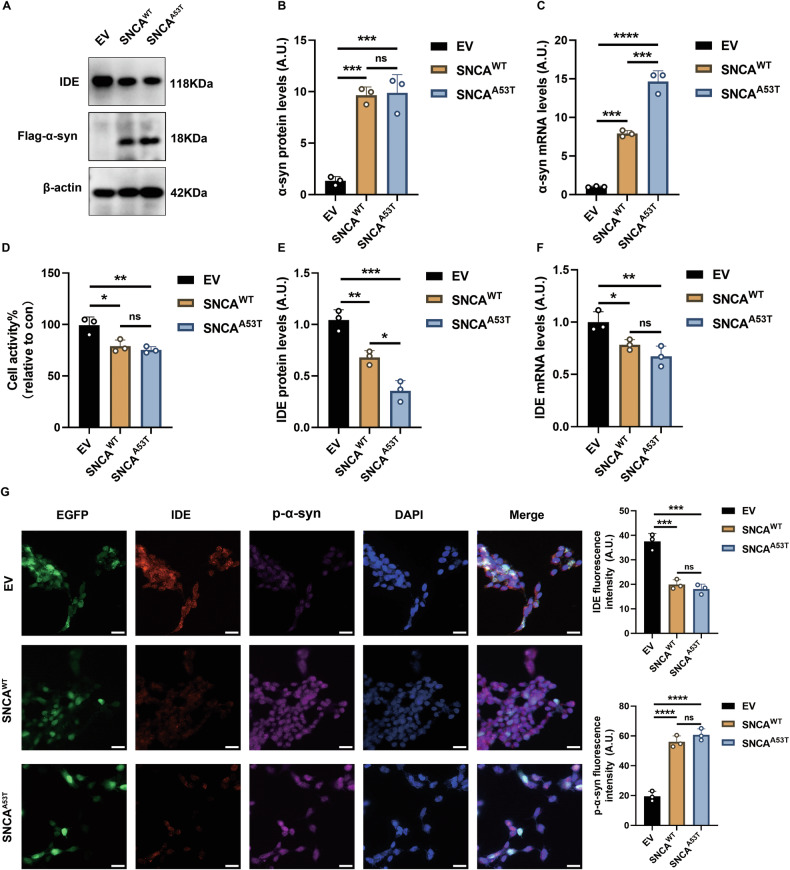
Reduced expression of IDE in SH-SY5Y PD model cells. **A** Representative western blotting of IDE and α-syn in the stably overexpressed α-syn of SH-SY5Y cells, which served as PD model cells. **B**, **C** Quantitative protein and transcription analyses of α-syn in PD model cells (*n* = 3, representing three independent experiments). ****P < 0.0001, ***P < 0.001, ns not significant; ANOVA analyses and Tukey’s test for post hoc comparisons. **D** Comparative analyses of the cytotoxicity in PD model cells via the cell viability of CCK-8 assays (*n* = 3, representing three independent experiments). **P < 0.01, *P < 0.05, ns not significant; ANOVA analyses and Tukey’s test for post hoc comparisons. **E**, **F** Quantitative protein and transcription analyses of IDE in PD model cells (*n* = 3, representing three independent experiments). ***P < 0.001, **P < 0.01, *P < 0.05, ns not significant; ANOVA analyses and Tukey’s test for post hoc comparisons. **G** Representative immunostaining images displayed the distribution of IDE and p-α-syn in PD model cells (*n* = 3, representing three independent experiments). The quantification of the fluorescence intensity was also calculated. Scale bar: 20 μm. ****P < 0.0001, ***P < 0.001, ns not significant; ANOVA analyses and Tukey’s test for post hoc comparisons.

To corroborate these in vivo findings, we next evaluated IDE levels in in vitro models of PD. In SH-SY5Y cells overexpressing WT (SNCA^WT^) or mutant (SNCA^A53T^) α-syn (Fig. 2A–C), SNCA^A53T^ expression significantly decreased cell viability, consistent with increased neurotoxicity (Fig. 2D). In α-syn–overexpressing cells, IDE protein and mRNA were reduced relative to empty vector (EV) controls (Fig. 2E–F). Phosphorylated α-synuclein (p-α-syn)—a PD-relevant post-translational modification [17]—was elevated, and immunofluorescence confirmed attenuated IDE staining coincident with increased p-α-syn signal (Fig. 2G). Together with the in vivo data, these results support an inverse relationship between α-synuclein load and IDE expression.

### Co-localization of IDE with α-syn in vivo, but limited direct interaction in vitro

**Fig. 3:**
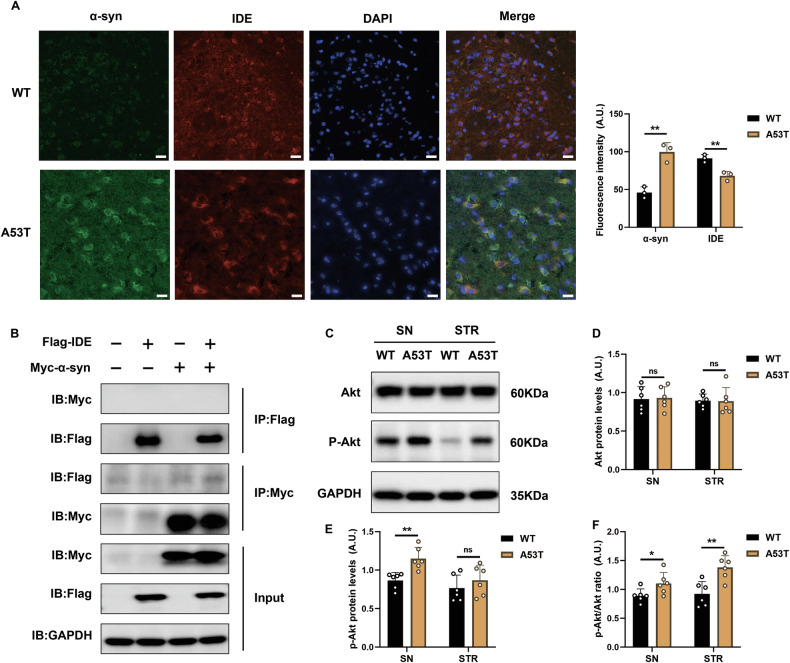
IDE co-localized with α-syn in vivo without direct interaction in vitro, and potentially activated the insulin signaling pathway. **A** Representative immunostaining images displayed the distribution of IDE and α-syn in the SN of 12-month-old A53T α-syn mice and WT mice (*n* = 3 per group). The quantification of the fluorescence intensity was calculated. Scale bar: 20 μm. **P < 0.01; Student’s *t* test. **B** The CO-IP assay with Flag-IDE and Myc-α-syn co-transfected into HEK-293T cells to uncover the binding between the IDE and α-syn. **C** The representative western blot bands of Akt and p-Akt about the SN and STR of 12-month-old A53T α-syn mice (*n* = 6) and WT control mice (*n* = 6). **D**–**F** Quantitative analyses including the Akt, p-Akt, and the ratio of p-Akt/Akt were performed (*n* = 6 per group). **P < 0.01, *P < 0.05, ns not significant; Student’s *t* test.

To investigate the association between the IDE and α-syn, we performed immunofluorescence colocalization in the SN of 12-month-old A53T α-syn mice and WT controls. Immunofluorometric assays in 12-month-old A53T α-syn mice illustrated reduced IDE fluorescence and partial co-localization of IDE and α-syn in the SN, consistent with their reported in vivo association (Fig. 3A). However, co-immunoprecipitation in HEK-293T cells transfected with Flag-IDE and Myc-α-syn did not detect a robust direct interaction (Fig. 3B). These data suggest that IDE-α-syn interactions may be more nuanced, possibly cell-type-dependent or mediated through additional co-factors in the PD-affected brain.

Moreover, IDE is crucial for insulin signaling by facilitating the clearance of aggregated toxic proteins. Notably, dysregulation of this pathway in the brains of PD patients can occur before the loss of dopaminergic neurons [9, 18]. Therefore, we observed elevated p-Akt levels in the SN and STR of A53T α-syn mice (Fig. 3C–F), indicating hyperactivation of insulin signaling, which may limit autophagy and promote α-syn accumulation [19]. These findings support the notion that dysregulated insulin signaling exacerbates PD-related pathology, and suggest that IDE deficiency could be part of this metabolic-protein aggregation cycle.

### AAV-mediated IDE overexpression restores IDE levels and suppresses α-syn in A53T α-syn mice

**Fig. 4:**
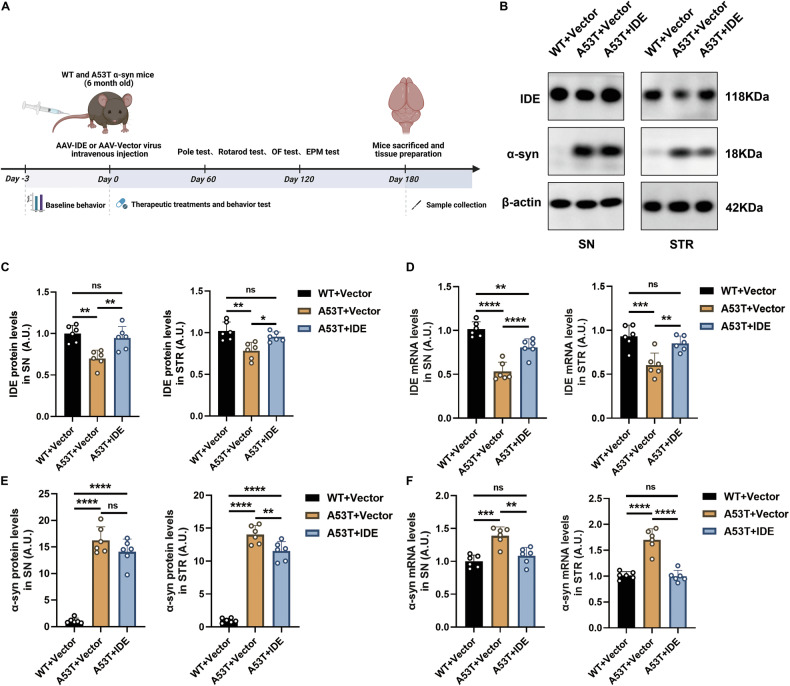
AAV‑mediated restoration of IDE reduces α‑syn deposition in A53T α‑syn mice. **A** Experimental timeline showing model establishment, AAV-IDE or control AAV-Vector administration, and behavioral testing. **B** Representative immunoblots of IDE and α-syn in SN and STR (*n* = 6 per group). **C**, **D** Quantification of IDE protein and mRNA in SN and STR tissues of WT + Vector, A53T + Vector, and A53T + IDE mice (*n* = 6 per group). ****P < 0.0001, ***P < 0.001, **P < 0.01, *P < 0.05, ns not significant; ANOVA analyses and Tukey’s test for post hoc comparisons. **E**, **F** Quantification of α-syn protein and mRNA in SN and STR across the same groups (*n* = 6 per group). ****P < 0.0001, ***P < 0.001, **P < 0.01, ns not significant; ANOVA analyses and Tukey’s test for post hoc comparisons.

To evaluate IDE’s therapeutic potential, we administered the AAV-IDE systemically via tail-vein injection to 6-month-old A53T α-syn mice and age-matched WT controls, creating three groups: WT + Vector, A53T + Vector, and A53T + IDE (Fig. 4A). At 180 days post-injection (*dpi*), IDE protein and mRNA levels were significantly elevated in the SN and STR of A53T + IDE mice compared with A53T + Vector mice (Fig. 4B–D). Consistent with previous findings, IDE expression remained lower in the SN and STR of A53T + Vector mice than in WT + Vector mice (Fig. 4B–D). Correspondingly, α-syn protein and mRNA levels were markedly reduced in the STR-and to a lesser extent, in the SN of A53T + IDE mice versus A53T + Vector mice (Fig. 4B, E, F), suggesting that IDE overexpression inhibits α-syn pathology.

### AAV-mediated IDE overexpression preserves behavior and dopaminergic neurons in A53T α-syn mice

Previous studies report that A53T α-syn mice develop age-dependent motor impairment and show reduced anxiety-/depression-like behaviors [20–22]. Consistent with these data, at baseline (6 months), WT and A53T α-syn mice did not differ in pole, rotarod, open field (OF), or elevated plus maze (EPM) tests (Supplementary Fig. S2A). Tail vein injection of AAV-IDE or AAV-Vector was followed by longitudinal assessment at 60, 120, and 180 day post injection (*dpi*). A53T+ Vector mice progressively deteriorated, showing slower pole test descent, reduced rotarod latency, hyperlocomotion in the OF, and longer open-arm time of EPM by 12 months (Fig. S2B–D). For A53T + IDE mice, IDE overexpression prevented these behavioral abnormalities (Supplementary Fig. S2B–D).

**Fig. 5:**
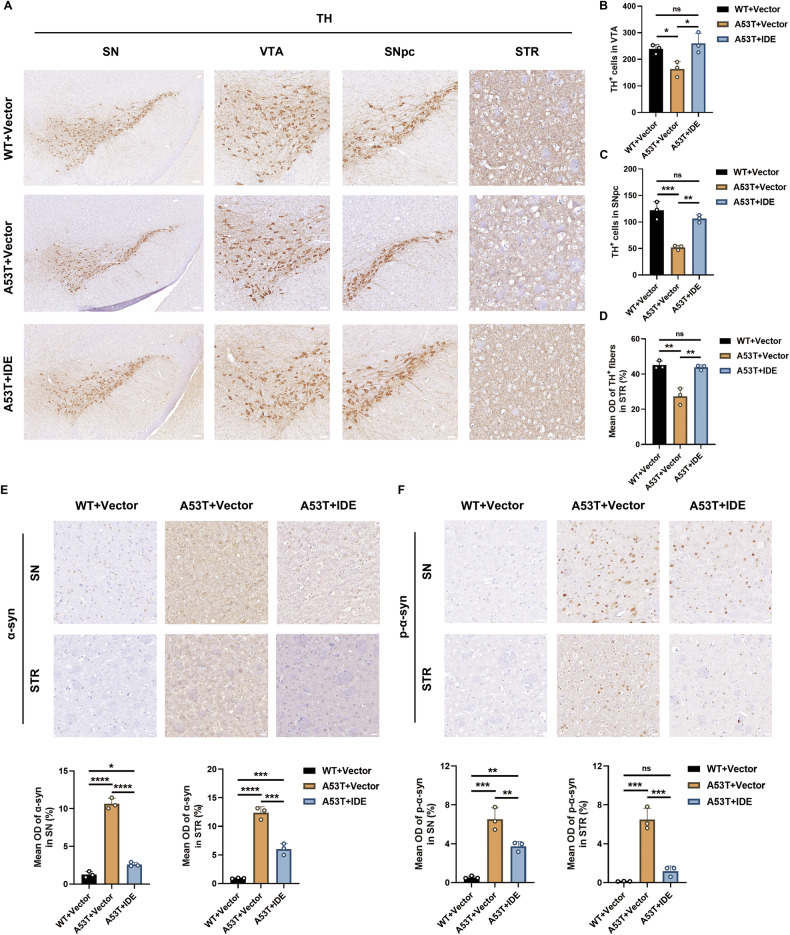
IDE restored dopaminergic neurons and decreased α-syn and p-α-syn accumulation in A53T α-syn mice. **A** Representative immunohistochemistry figures of TH in the SN and STR of WT + Vector, A53T + Vector, and A53T + IDE mice (*n* = 3 per group). Scale bar for SN: 100 μm, for ventral tegmental area (VTA) and substantia nigra pars compacta (SNpc): 50 μm, for STR: 20 μm. **B**, **C** TH immunohistochemical staining of the SN were separately tested in the zone of VTA and SNpc in WT + Vector, A53T + Vector, and A53T + IDE mice. The quantifications of the TH-positive cells were calculated in succession (*n* = 3 per group). ***P < 0.001, **P < 0.01, *P < 0.05, ns not significant; ANOVA analyses and Tukey’s test for post hoc comparisons. **D** Quantification of TH in the STR of the WT + Vector, A53T + Vector, and A53T + IDE groups (*n* = 3 per group). **P < 0.01, ns not significant; ANOVA analyses and Tukey’s test for post hoc comparisons. **E**, **F** α-syn and p-α-syn immunostaining in the SN and STR of WT + Vector, A53T + Vector, and A53T + IDE mice were determined and quantitatively analyzed (*n* = 3 per group). Scale bar, 20 μm.****P < 0.0001, ***P < 0.001, **P < 0.01, *P < 0.05, ns not significant; ANOVA analyses and Tukey’s test for post hoc comparisons.

The hallmark pathological feature of PD is the selective loss of dopaminergic neurons, commonly identified by TH expression [23]. TH immunostaining revealed a loss of midbrain dopaminergic neurons in A53T + Vector mice at 12 months. IDE restored TH-positive cell density and normalized striatal TH-fiber density (Fig. 5A–D). Total and phosphorylated α-syn immunoreactivity fell in A53T + IDE versus A53T + Vector mice (Fig. 5E, F). Therefore, IDE gene delivery maintains nigrostriatal integrity and prevents α-syn–driven behavioral decline in A53T α-syn mice.

### IDE overexpression mitigates α-syn-induced toxicity in PD model cells, while IDE knockdown exacerbates neurodegeneration

To determine IDE’s protective capacity in vitro, we manipulated IDE expression (overexpression or knockdown) in SH-SY5Y cells stably expressing wild-type (SNCA^WT^) or mutant (SNCA^A53T^) α-syn (PD model cells). Compared with LV-Con infection, LV-IDE robustly elevated IDE expression, reduced α-syn levels, improved cell viability (CCK-8 assay), and diminished cytotoxicity in both SNCA^WT^ and SNCA^A53T^ cells (Supplementary Fig. S3A–E). Immunofluorescence further confirmed less p-α-syn intensity in IDE-overexpressing PD cells (Supplementary Fig. S3F).

Conversely, compared with negative control (NC), IDE-siRNA markedly depleted IDE protein and mRNA, increased α-syn expression, exacerbated cell death, and aggravated neurotoxicity in PD model cells (Supplementary Fig. S3G–K). Notably, IDE downregulation also markedly increased the p-α-syn intensity in PD model cells (Supplementary Fig. S3L). Representative immunofluorescence images for IDE gain- and loss-of-function conditions are provided in Supplementary Figs. S4 and S5. Collectively, these data underscore that IDE modulates α-syn accumulation and is essential for neuronal survival in vitro.

### Multi-omics analyses reveal Hippo signaling as a key downstream pathway of IDE-mediated neuroprotection

**Fig. 6:**
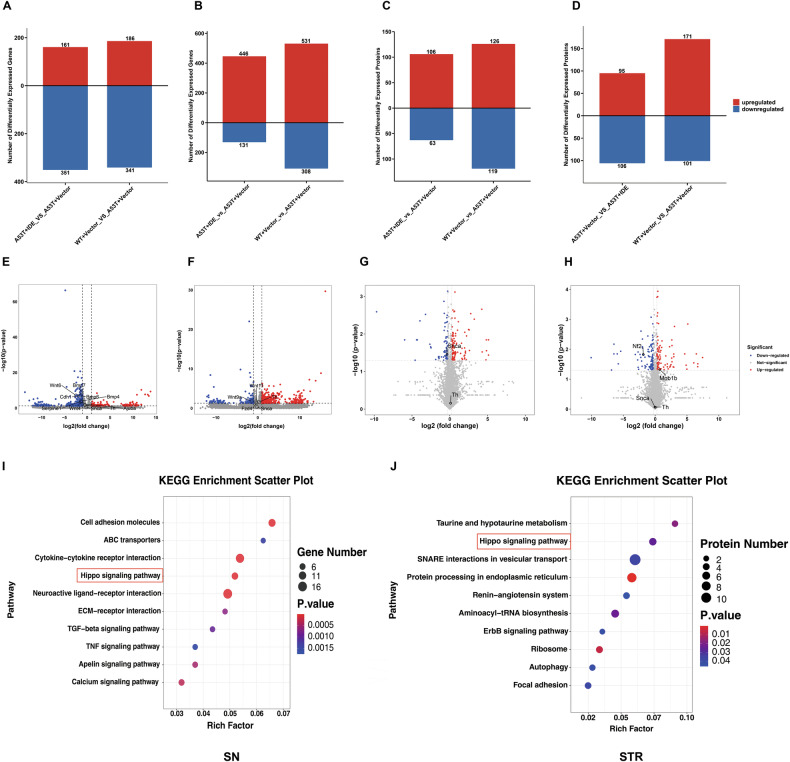
Proteome and transcriptome analyses revealing the Hippo signaling pathway mediating the neuroprotective effects of IDE. Comparative statistical histogram of DEGs in the SN (**A**) and STR (**B**) of each group of mice (*n* = 3 each group). Quantitative results of DEPs in the SN (**C**) and STR (**D**) of each group of mice (*n* = 3 per group). The volcano plots between A53T + IDE/A53T + Vector groups revealed the up (red) or downregulated (blue) DEGs in SN (**E**) and STR (**F**) in mice (*n* = 3 per group). Volcano plot of DEPs associated with the SN (**G**) and STR (**H**) in A53T + IDE/A53T + Vector groups, with significant upregulations as red dots and significant downregulations as blue dots (*n* = 3 per group). Considering all DEGs and DEPs in A53T + IDE/A53T + Vector groups, Kyoto Encyclopedia of Genes and Genomes (KEGG) analyses identified the top 10 pathways in the transcriptome in SN (**I**), along with the proteomics in STR (**J**), respectively (*n* = 3 per group).

To elucidate IDE’s molecular mechanism, we conducted RNA sequencing (RNA-seq) and data-independent acquisition (DIA) proteomics on the SN and STR of A53T + Vector and A53T + IDE mice. We then mapped differentially expressed genes (DEGs) and differentially expressed proteins (DEPs) in the nigrostriatal pathway to Kyoto Encyclopedia of Genes and Genomes (KEGG) pathways for subsequent bioinformatic analyses. The related data were summarized in Supplementary Tables S3 and S4. In the SN, IDE treatment led to 161 upregulated and 351 downregulated DEGs (Fig. 6A), whereas 446 upregulated and 131 downregulated DEGs were identified in the STR (Fig. 6B). Simultaneously, we detected 106 upregulated and 63 downregulated DEPs in the SN (Fig. 6C) and 95 upregulated and 106 downregulated DEPs in the STR (Fig. 6D).

Representative volcano plots of these DEGs (Fig. 6E, F) and DEPs (Fig. 6G, H) highlighted enriched gene sets in the SN and STR. We subsequently subjected the DEGs from the SN and the DEPs from the STR to KEGG pathway analyses. Notably, the Hippo signaling pathway ranked among the top enriched pathways in both the SN transcriptome and the STR proteome (Fig. 6I, J). These results further illuminate IDE’s protective role in PD, partly through modulating the Hippo signaling pathway.

### IDE gene delivery suppresses Hippo/insulin signaling and limits neuronal apoptosis

Multi-omics enrichment flagged the Hippo cascade as a major dysregulated pathway in A53T α-syn mice. Its core kinases MST1/2 and co-activator Yes-associated protein (YAP), together with their phosphorylated forms, regulate neuronal apoptosis in PD [24–26]. Accordingly, we measured MST1/2, p-MST1/2, MOB kinase activator 1b (Mob1b), YAP, and p-YAP to assess Hippo activity. Western blot confirmed the increases in MST1/2, p-MST1/2, Mob1b, and p-YAP, with a reduction in total YAP in A53T + Vector versus WT + Vector mice (Supplementary Fig. S6). AAV-IDE normalized these proteins and partially restored YAP levels (Supplementary Fig. S6). Insulin signaling was likewise hyper-activated, as shown by elevated phosphorylation of insulin receptor substrate 1 (p-IRS-1) and p-Akt. IDE treatment reversed both changes (Supplementary Fig. S7).

**Fig. 7:**
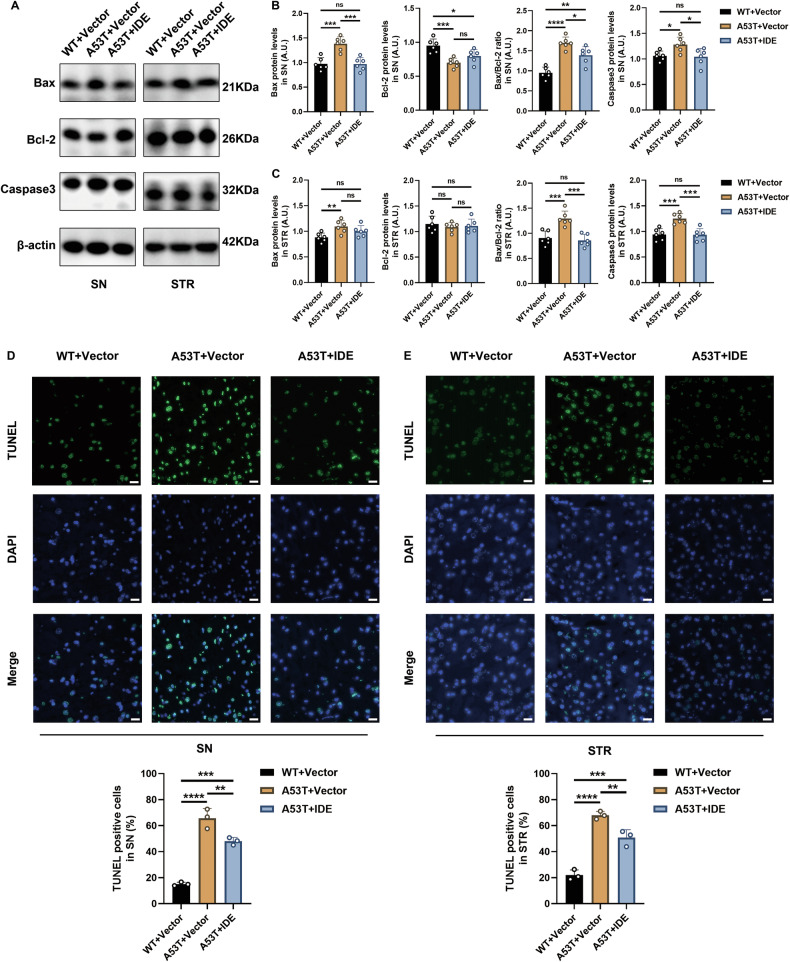
Protective effects of IDE on neuronal apoptosis of Hippo signaling pathways in A53T α-syn mice. **A** Representative western blot bands of Bax, Bcl-2, and Caspase 3 in the SN and STR of WT + Vector, A53T + Vector, and A53T + IDE mice (*n* = 6 per group). **B**, **C** The corresponding quantitative analyses of Bax, Bcl-2, the ratio of Bax/Bcl-2, and Caspase 3 were performed (*n* = 6 per group). ****P < 0.0001, ***P < 0.001, **P < 0.01, *P < 0.05, ns not significant; ANOVA analyses and Tukey’s test for post hoc comparisons. **D**, **E** Representative TUNEL staining (green) images and DAPI (blue) and statistical quantification of TUNEL^+^ cells in the SN and STR of WT + Vector, A53T^+^Vector, and A53T + IDE mice (*n* = 3 per group). Scale bar, 20 μm. ****P < 0.0001, ***P < 0.001, **P < 0.01; ANOVA analyses and Tukey’s test for post hoc comparisons.

Consistent with Hippo-mediated apoptosis [27–29], A53T+Vector mice displayed higher Bax, an increased Bax/Bcl-2 ratio, and greater Caspase 3 in the SN and STR (Fig. 7A–C). IDE overexpression reduced these markers and TUNEL-positive neurons (Fig. 7D, E). These data demonstrate that IDE mitigates MST1/2-driven Hippo activation, corrects downstream insulin signaling, and limits dopaminergic neuron apoptosis in A53T α-syn mice.

### MST1/2 inhibition (XMU-MP-1) synergizes with IDE to reduce α-syn toxicity in PD model cells

**Fig. 8:**
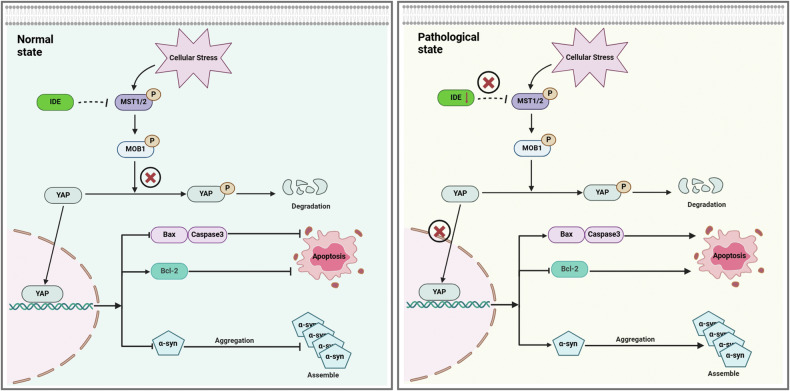
Schematic summary of mechanism underlying the neuroprotective effects of IDE in PD. Based on our findings, IDE exerts anti-PD effects by inhibiting the Hippo signaling pathway via MST1/2 targets. In the normal state, the constitutively expressed IDE inhibited the decline of p-MST1/2, p-Mob1b, and p-YAP, along with the decline of MST1/2, Mob1b, and the induction of YAP (precluded from the degradation of phosphorylated proteins), maintaining the homeostasis of the Hippo signaling pathways. Thereby, IDE decreased the assembly of α-syn and the neurotoxicity by apoptosis of the IDE-Hippo axis (left panel). In contrast, under pathological conditions of PD, decreased IDE levels lost the ability to cope well with intracellular stress, and the dysregulated Hippo signaling pathways increased the p-MST1/2, p-Mob1b, and p-YAP, along with the increase of MST1/2 and Mob1b, and lowered the YAP levels. Jointly, the series of alterations finally aggravated the accumulation of α-syn and the neuronal apoptosis via increasing Bax and Caspase 3 level, and decreasing the level of Bcl-2, leading to the progression of PD (right panel).

Finally, we tested whether pharmacological MST1/2 inhibition by XMU-MP-1 could further enhance cell viability in PD model cells. Pharmacokinetic analysis showed that XMU-MP-1 binds MST1/2 on-target and blocks their kinase activity, thereby activating the downstream effector YAP and inhibiting the Hippo signaling pathway [30]. According to a previous report [16], we used the indicated dose of XMU-MP-1 for 24 h. Indeed, XMU-MP-1 decreased p-MST1/2 levels, which displayed the inhibition of the Hippo pathway (Supplementary Fig. S8A–D). Interestingly, XMU-MP-1 effectively rescued neuronal apoptosis in PD model cells by reducing pro-apoptotic markers (Bax, Bax/Bcl-2 ratio, and Caspase 3) and increasing Bcl-2 levels (Supplementary Fig. S8A, E–H). Furthermore, CCK-8 assays, transcription analyses, and immunoblot both confirmed reduced α-syn levels and improved viability in α-syn-overexpressing PD cells (Supplementary Fig. S8A, I–M). Collectively, these results underscore the IDE-Hippo axis as a pivotal checkpoint for neuroprotection in PD (Fig. 8).

## Discussion

The intricate connection between PD and T2DM underscores the need to identify shared pathogenic factors. IDE has emerged as a promising therapeutic target by degrading islet amyloid, α-synuclein, and other substrates, thereby hindering disease progression [31, 32]. In this study, we observed that IDE levels were diminished in the brains of homozygous A53T α-syn transgenic PD mice. Overexpressing IDE in these mice alleviated motor deficits and neuropathology, in part by suppressing the Hippo signaling pathway. Moreover, IDE attenuated α-syn-induced neurotoxicity and neuronal apoptosis in SH-SY5Y PD model cells, partly by inhibiting MST1/2 in the Hippo signaling pathway. By integrating in vivo and in vitro PD models, multi-omics analyses, and pharmacological MST1/2 inhibition, our results highlight the IDE-Hippo axis as a central checkpoint for neuroprotection in PD (Fig. 8).

IDE, implicated in both T2DM and PD, serves as a vital factor in clearing aggregated substrates such as islet amyloid and α-syn, underscoring its important role in PD [33]. In addition to its degradative function, IDE is also proposed to act as a “dead-end” chaperone, a heat shock protein, and an E1 ubiquitin-activating enzyme, highlighting additional non-degradative roles in neurodegenerative and metabolic diseases [9, 34]. Consistently, reduced IDE levels have been reported in IDE-associated conditions like T2DM and AD [35, 36]. We therefore hypothesized that IDE is critical in PD pathogenesis, consistent with our preliminary data linking IDE variants to sporadic PD (unpublished). However, a proteomic study of SN tissues from 15 PD patients and 15 controls did not detect significant alterations in IDE expression [15]. In this study, we found that IDE expression was diminished in 12- and 16-month-old homozygous A53T α-syn PD mice, but not at 6 months of age. Consistently, IDE was also reduced in SH-SY5Y PD model cells overexpressing wild-type or A53T α-syn. Interestingly, 12-month-old A53T α-syn mice exhibited significantly elevated fasting serum IDE compared with WT controls, suggesting a possible compensatory mechanism. These findings imply that IDE’s role in PD may vary across different disease stages. Moreover, the discrepancy between brain and serum IDE levels might reflect a compensatory response, wherein decreased IDE in the brain prompts elevated peripheral IDE. The specific role of IDE in PD pathogenesis has been less explored, but several mechanisms have been proposed. First, IDE may directly inhibit α-syn fibrillization by binding its C-terminal domain via a non-proteolytic mechanism [11, 12]. Second, defective IDE may interfere with α-syn clearance through autophagy and proteasome systems, as per the “dead-end” chaperone hypothesis [9]. Finally, lower IDE levels could disrupt central insulin signaling and metabolic homeostasis, thereby increasing neuronal vulnerability and dopaminergic cell loss [37]. In our study, we assessed IDE’s protective effects on motor deficits, neuropathology, and cytotoxicity in both in vivo and in vitro PD models. Specifically, 6-month-old A53T α-syn mice received AAV-IDE or AAV-Vector, and at 180 days post-injection, IDE overexpression improved motor function and alleviated pathology, including reductions in α-syn/p-α-syn deposition and dopaminergic neuron loss. Likewise, IDE overexpression suppressed α-syn levels and attenuated neurotoxicity, whereas IDE knockdown heightened α-syn pathology and cell death in SH-SY5Y PD model cells. Together, these findings identify IDE as a promising disease-modifying factor in PD.

Previous studies have shown that IDE is involved in T2DM and AD [14, 38, 39]. Meanwhile, α-syn aggregation and insulin signaling dysfunction are recognized as major contributors to PD pathogenesis [9, 40]. Our findings extend these observations by indicating that insulin pathway perturbations and α-syn accumulation may converge more extensively than previously appreciated. In this study, we observed hyperactivation of the insulin signaling pathway in A53T α-syn mice, evidenced by elevated p-Akt levels. Heightened mTOR signaling, secondary to this insulin pathway activation, may suppress autophagy, potentially explaining the observed phenotype in PD mice [19]. Notably, IDE overexpression rescued this aberrant insulin signaling pathway in A53T α-syn mice. The partial restoration of p-Akt and p-IRS-1 in A53T + IDE mice suggests that IDE may normalize insulin signaling, although further functional assays are required to confirm potential synergy between IDE’s enzymatic activity and insulin pathways.

Beyond its canonical role in growth control, the Hippo signaling pathway has emerged as a key regulator of neuronal survival. In mammals, pathway activation triggers MST1/2-mediated phosphorylation of LATS1/2, which in turn phosphorylates and destabilizes YAP, driving mitochondrial fragmentation, neuro-inflammation, and apoptosis—processes documented in both Parkinson’s and Alzheimer’s disease models [24, 28, 41, 42]. Pharmacological or genetic suppression of MST1/2 interrupts this cascade—lowering Bax and Caspase 3, raising Bcl-2 and preserving dopaminergic neurons; the ATP-competitive inhibitor XMU-MP-1 is a prototypical compound that accomplishes these effects in vivo [16, 24, 41]. Notably, MST1/2 inhibition also frees WWC1/KIBRA from LATS1/2, enriching AMPAR complexes and enhancing synaptic transmission, a mechanism linked to cognitive improvement in neurodegenerative settings [43, 44]. Our transcriptomic-proteomic screens place MST1/2, Mob1b, and YAP at the center of IDE-mediated protection. Reduced IDE amplifies Hippo activity, increasing Bax, Caspase activation, and TUNEL-positive cells, whereas IDE overexpression—or XMU-MP-1 administration—suppresses MST1/2 signaling and mitigates α-synuclein toxicity. We note, however, that MST1/2 can facilitate mitophagy under certain stresses [45], underscoring the context-dependent nature of Hippo components. Our data reinforce the view that an IDE–Hippo axis governs neuronal apoptosis, inflammation, and mitochondrial integrity in PD, and highlight MST1/2 modulation as a nuanced yet promising therapeutic target.

Our study identifies a previously unrecognized IDE-Hippo signaling axis: raising IDE activity or suppressing the Hippo kinases MST1/2 converges to curb α-synuclein aggregation and dopaminergic cell loss. Although the work is still proof-of-concept, it points to a translational strategy that couples metabolic/insulin-based approaches (to restore IDE) with brain-penetrant MST1/2 inhibitors for advanced Parkinson’s disease. However, the current evidence is confined to rodents and immortalized cell lines. Measuring IDE activity in human PD tissue across disease stages—and in patient-derived midbrain organoids now under investigation—will be critical for clinical validation. While in-vitro co-immunoprecipitation showed minimal IDE-α-syn binding, in-vivo co-localization and prior reports hint at a context-dependent interaction. Structural or cross-linking studies should clarify whether IDE’s C-terminal domain engages α-syn monomers or oligomers in an ATP- or insulin-dependent manner. Our data suggest that IDE augmentation and MST1/2 inhibition act synergistically. Future experiments combining these interventions in PD models could confirm additive neuroprotection and lay the groundwork for multi-target therapies.

In conclusion, IDE appears to be a potent neuroprotective factor in PD, mitigating α-syn-mediated pathology partly by suppressing the Hippo signaling pathway (Fig. 8). These findings enhance our understanding of how metabolism-linked enzymes and apoptotic signaling intersect in PD progression, providing a strong rationale for IDE- and MST1/2-targeted interventions in future translational studies.

## Supplementary information

Supplementary material

Original data

Reproducibility checklist

## Data Availability

The datasets in this article are available from the corresponding author upon reasonable request.
