# Supplementary material for: Insulin-degrading enzyme confers neuroprotection in Parkinson’s disease by inhibiting the Hippo signaling pathway
Source: Cell Death Dis. 2025 Oct 24;16(1):758. doi: 10.1038/s41419-025-08055-4 (PMC12552454; doi:10.1038/s41419-025-08055-4)
Supplement: Supplementary file 1 — Supplementary material [file 41419_2025_8055_MOESM1_ESM.pdf]

Supplementary Material

Insulin-Degrading Enzyme Confers Neuroprotection in Parkinson's Disease by Inhibiting the Hippo Signaling Pathway

Huimin Zheng [1, 2, 3, 4 †], MD; Yu Guo [2, 5†], MD; Shuyu Zhang [1†], MD; Yun Su [1, 2, 3, 4], MD; Xin Cui [1, 2, 3, 4], MD; Zhengwei Hu [1, 2, 3, 4], MD, PhD; Xiaoyan Hao [1, 2, 3, 4], MD; Mengjie Li [1, 2, 3, 4], MD; Changhe Shi [1, 2, 3, 4], MD, PhD; Yuming Xu [1, 2, 3, 4*], MD, PhD; Chengyuan Mao [1, 2, 3, 4*], MD, PhD

***Correspondence:**

Chengyuan Mao: maochengyuan2015@126.com

Yuming Xu: xuyuming@zzu.edu.cn

List of contents for Supplementary Material:

Supplementary Figures

Fig. S1: Assessments of TH levels, IDE levels, and IDE enzyme activity in A53T α-syn mice.

Fig. S2: Assessments of the effects of AAV-mediated IDE overexpression on the motor and non-motor behaviors in A53T α-syn mice.

Fig. S3: Assessments of the modulations of IDE overexpression and knockdown in PD model cells.

Fig. S4: Immunostaining figures of p-α-syn in SH-SY5Y PD model cells with the infection of LV-Con and LV-IDE.

Fig. S5: Immunostaining figures of p-α-syn in SH-SY5Y PD model cells with the transfection of NC and IDE-siRNA.

Fig. S6: Western blot bands of MST1/2, p-MST1/2, Mob1b, YAP, and p-YAP in the SN and STR of WT+Vector, A53T+Vector, and A53T+IDE mice.

Fig. S7: Western blot bands of IRS-1, p-IRS-1, Akt, and p-Akt in the SN and STR of WT+Vector, A53T+Vector, and A53T+IDE mice.

Fig. S8: Assessments of the critical role of MST1/2 for inhibiting Hippo signaling in PD model cells.

Supplementary Tables

Table S1: Antibodies

Table S2: Primer and siRNA

Table S3: Differentially expressed genes in the proteomes of the SN and STR of A53T+IDE/A53T+Vector mice.

Table S4: Differentially expressed proteins in the proteomes of the SN and STR of A53T+IDE/A53T+Vector mice.

Supplementary Figures

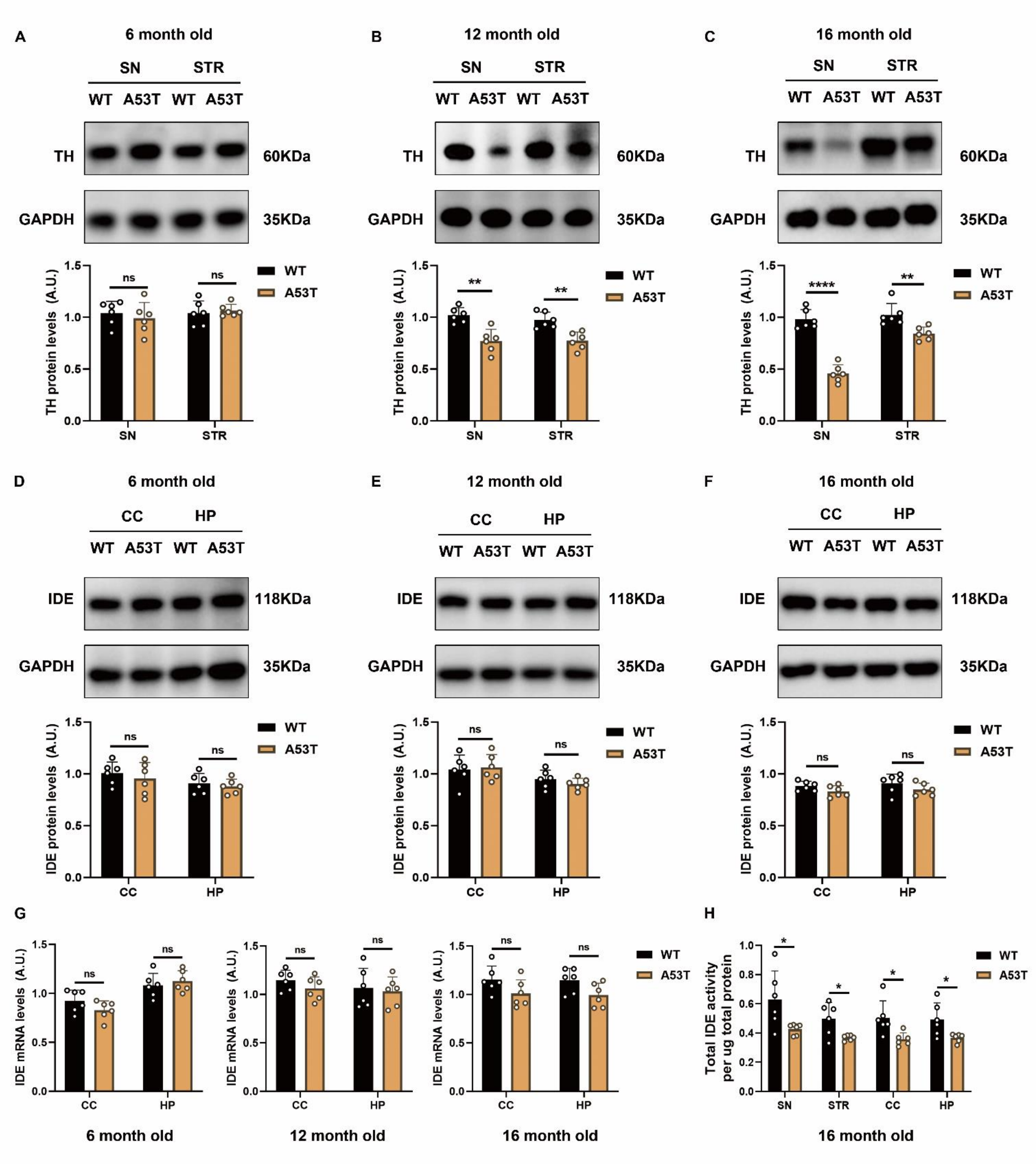

Fig. S1 Decreased IDE enzyme activity in A53T α-syn mice with dopaminergic degeneration. (A-C) TH protein levels were assessed in SN and STR of 6, 12, 16-month-old A53T α-syn mice (n=6) and WT control mice (n=6). Corresponding quantified results were also shown. **** *$P < 0.0001$, ** $P < 0.01$, ns, not significant; Student's t-test.* (D-F) IDE protein levels were quantified in cerebral cortex (CC) and hippocampus (HP) from of 6, 12, 16-month-old A53T α-syn mice and WT control mice (n=6 per group). Related statistical analysis results were also represented. *ns, not significant; Student's t-test.* (G) Comprehensive transcription analyses for CC and HP exhibiting the expression levels of IDE mRNA in 6, 12, 16-month-old A53T α-syn mice (n=6) and WT control mice (n=6). *ns, not significant; Student's t-test.* (H) Representing the IDE enzyme activity of 16-month-old A53T α-syn mice (n=6) and WT control mice (n=6) were evaluated in SN, STR, CC, and HP. * *$P < 0.05$; Student's t-test.*

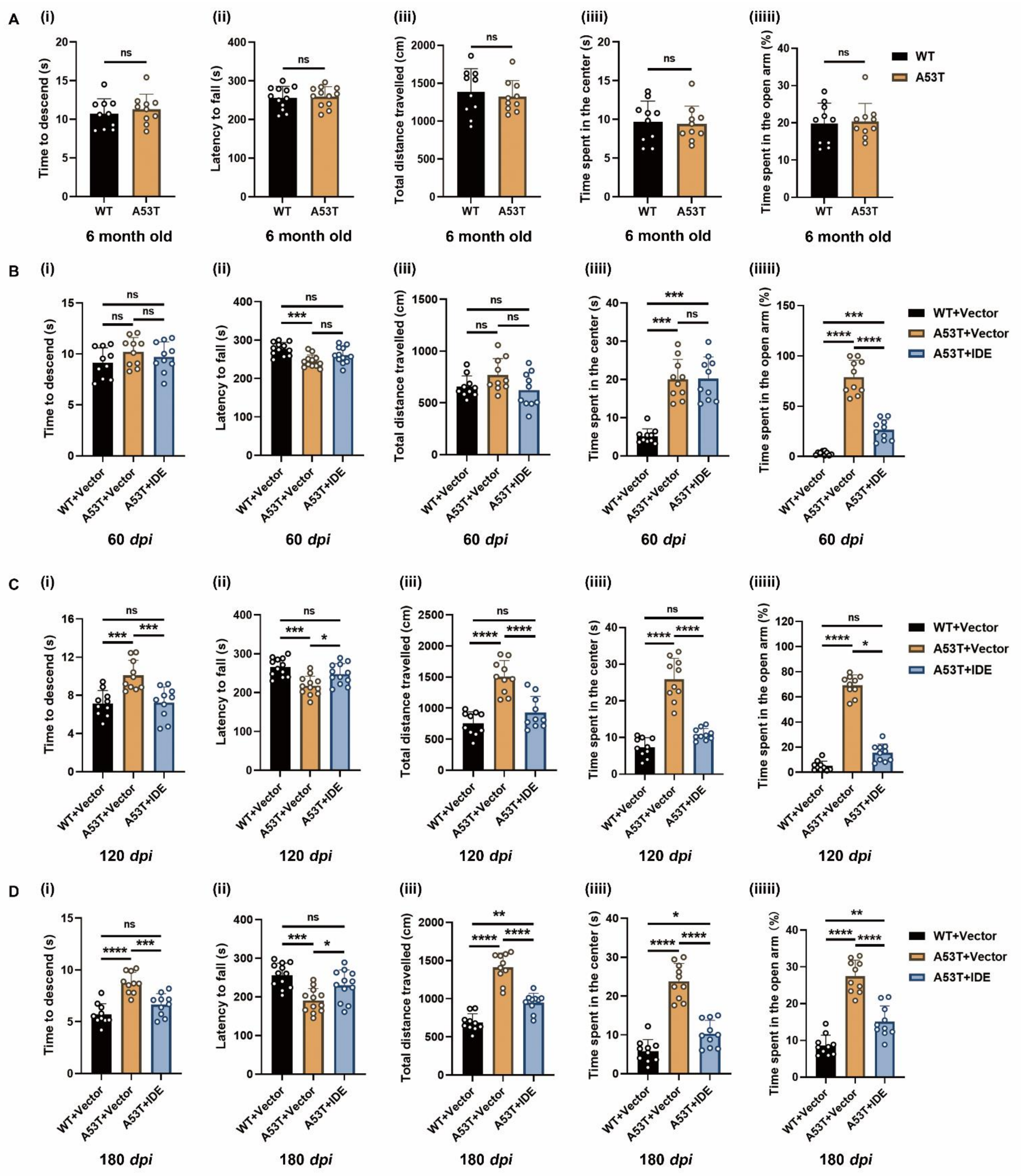

A
(i)
Time to descend (s)
WT
A53T
6 month old
(ii)
Latency to fall (s)
(iii)
Total distance travelled (cm)
(iiii)
Time spent in the center (s)
(iiiii)
Time spent in the open arm (%)
ns
B
C
D
WT+Vector
A53T+Vector
A53T+IDE
60 dpi
120 dpi
180 dpi

Fig. S2 IDE attenuated motor deficits and reversed the anti-anxiety-like behaviors in A53T α-syn mice. All (i) images represented the pole test, (ii) images represented the rotarod test, and (iii) and (iiii) images represented the OF test. Also, overall (iiiii) images represented the EPM test. (A) Baseline behavioral data including the pole test, the rotarod test, the OF test, and the EPM test to explore the motor and non-motor symptoms (n=10-12 per group). *ns, not significant; Student's t-test.* (B) 60 *dpi* of AAV-IDE or AAV-Vector in mice, their motor and non-motor abilities were tested (n=10-12 per group). ***** $P < 0.0001$, *** $P < 0.001$, ns, not significant; ANOVA analyses and Tukey's test for post hoc comparisons.* (C) 120 *dpi* of AAV-IDE or AAV-Vector in mice, their motor and non-motor functions were evaluated (n=10-12 per group). ***** $P < 0.0001$, *** $P < 0.001$, * $P < 0.05$, ns, not significant; ANOVA analyses and Tukey's test for post hoc comparisons.* (D) 180 *dpi* of AAV-IDE or AAV-Vector in mice, their motor and non-motor phenotypes were assessed (n=10-12 per group). ***** $P < 0.0001$, *** $P < 0.001$, ** $P < 0.01$, * $P < 0.05$, ns, not significant; ANOVA analyses and Tukey's test for post hoc comparisons.*

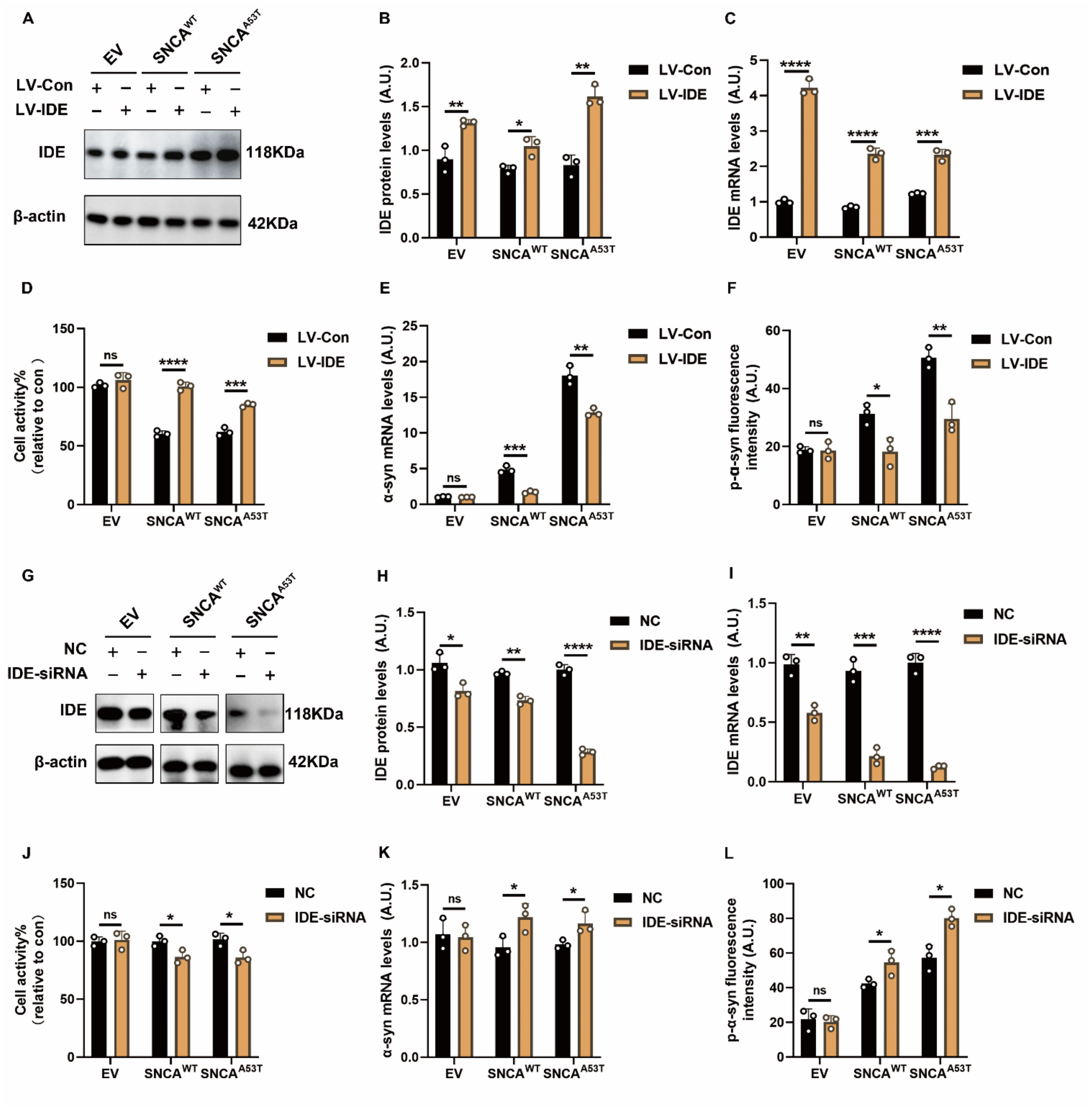
A
EV
SNCA^WT
SNCA^A53T
LV-Con
LV-IDE
IDE
118KDa
β-actin
42KDa
B
IDE protein levels (A.U.)
LV-Con
LV-IDE
C
IDE mRNA levels (A.U.)
D
Cell activity% (relative to con)
E
α-syn mRNA levels (A.U.)
F
p-α-syn fluorescence intensity (A.U.)
G
NC
IDE-siRNA
H
I
J
K
L
ns

Fig. S3 IDE protected PD model cells with decreased α-syn, while IDE knockdown aggravated the neurotoxicity. (A, B) Representative western blot bands and the quantitative analyses of IDE after the infection of LV-Con and LV-IDE in PD model cells. (n=3, representing three independent experiments). ** *$P < 0.01$, * $P < 0.05$; Student's t-test.* (C) Quantitative transcription analyses of IDE after the infection of LV-Con and LV-IDE in PD model cells (n=3, representing three independent experiments). **** *$P < 0.0001$, *** $P < 0.001$; Student's t-test.* (D) Cell viability of PD model cells with the infection of LV-Con and LV-IDE were identified by CCK-8 assays. (n=3, representing three independent experiments). **** *$P < 0.0001$, *** $P < 0.001$, ns, not significant; Student's t-test.* (E) Quantitative transcription analyses of α-syn after the infection of LV-Con and LV-IDE in PD model cells (n=3, representing three independent experiments). *** *$P < 0.001$, ** $P < 0.01$, ns, not significant; Student's t-test.* (F) The relevant quantification of the fluorescent intensity of p-α-syn after the infection of LV-Con and LV-IDE in PD model cells (n=3, representing three independent experiments). ** *$P < 0.01$, * $P < 0.05$, ns, not significant; Student's t-test.* (G, H) Representative western blotting and related densitometry analyses of IDE in PD model cells treated with NC and IDE-siRNA. (n=3, representing three independent experiments). **** *$P < 0.0001$, ** $P < 0.01$, * $P < 0.05$; Student's t-test.* (I) Relative mRNA levels of IDE were quantified after the NC and IDE-siRNA transfection in PD model cells (n=3, representing three independent experiments). **** *$P < 0.0001$, *** $P < 0.001$, ** $P < 0.01$; Student's t-test.* (J) The effect of NC and IDE-siRNA on the cell viability of PD model cells were measured via CCK-8 assays. (n=3, representing three independent experiments). * *$P < 0.05$, ns, not significant; Student's t-test.* (K) Relative mRNA levels of α-syn were quantified after the NC and IDE-siRNA transfection in PD model cells (n=3, representing three independent experiments). * *$P < 0.05$, ns, not significant; Student's t-test.* (L) The fluorescent intensity of p-α-syn were separately quantified in PD model cells with the transfection of NC and IDE-siRNA. (n=3, representing three independent experiments). * *$P < 0.05$, ns, not significant; Student's t-test.*

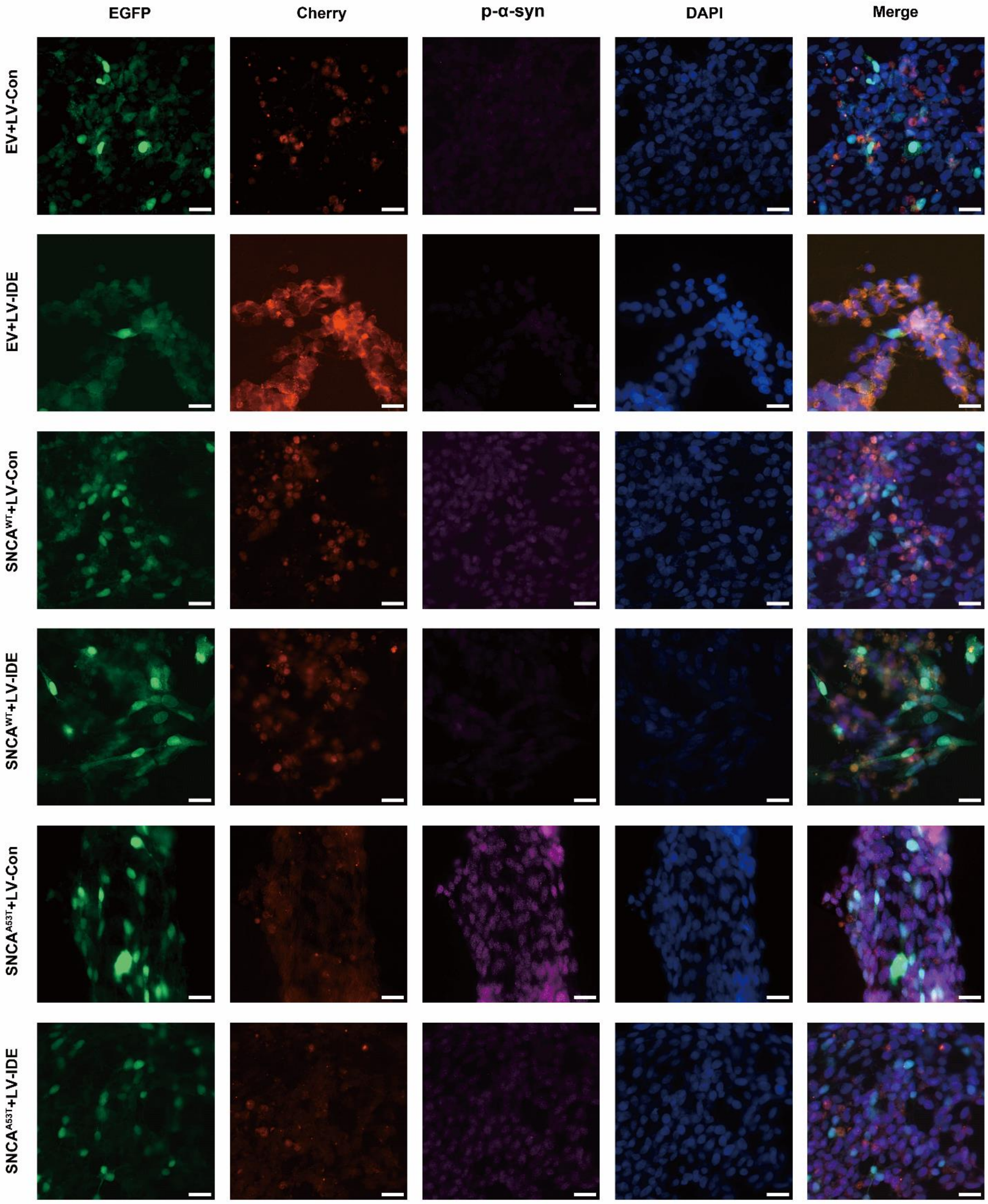
EGFP
Cherry
p-α-syn
DAPI
Merge
EV+LV-Con
EV+LV-IDE
SNCAWT+LV-Con
SNCAWT+LV-IDE
SNCAA53T+LV-Con
SNCAA53T+LV-IDE

Fig. S4 Overexpressed IDE alleviated the levels of p-α-syn in SH-SY5Y PD model cells. Representative immunostaining figures of p-α-syn in SH-SY5Y PD model cells with the infection of LV-Con and LV-IDE (n=3, representing three independent experiments). Scale bar: 20 μm**.**

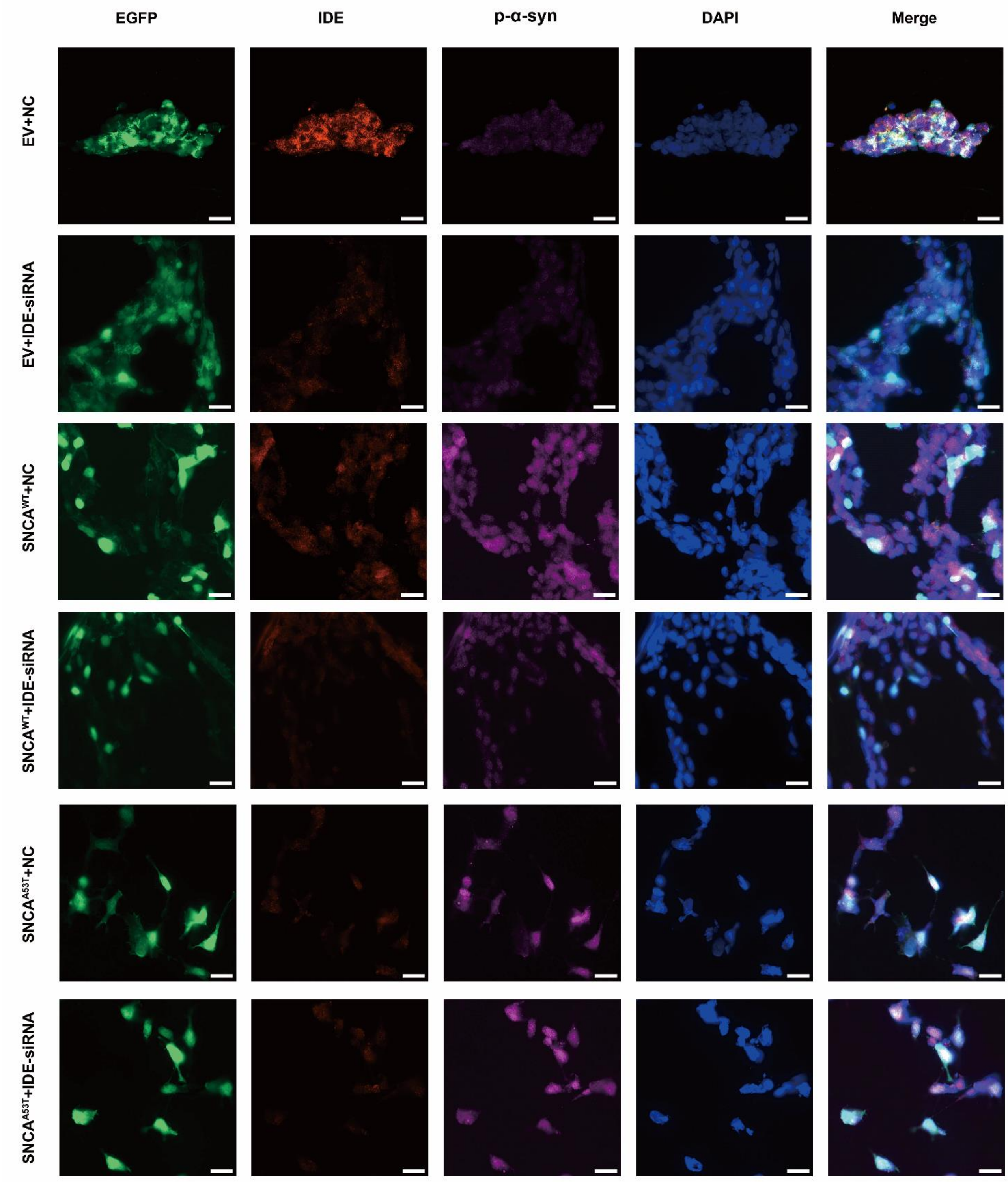
EGFP
IDE
p-α-syn
DAPI
Merge
EV+NC
EV+IDE-siRNA
SNCAWT+NC
SNCAWT+IDE-siRNA
SNCAA53T+NC
SNCAA53T+IDE-siRNA

Fig. S5 Knock-down IDE aggravated the levels of p-α-syn in SH-SY5Y PD model cells. Representative immunostaining figures of p-α-syn in SH-SY5Y PD model cells with the transfection of NC and IDE-siRNA (n=3, representing three independent experiments). Scale bar: 20 μm**.**

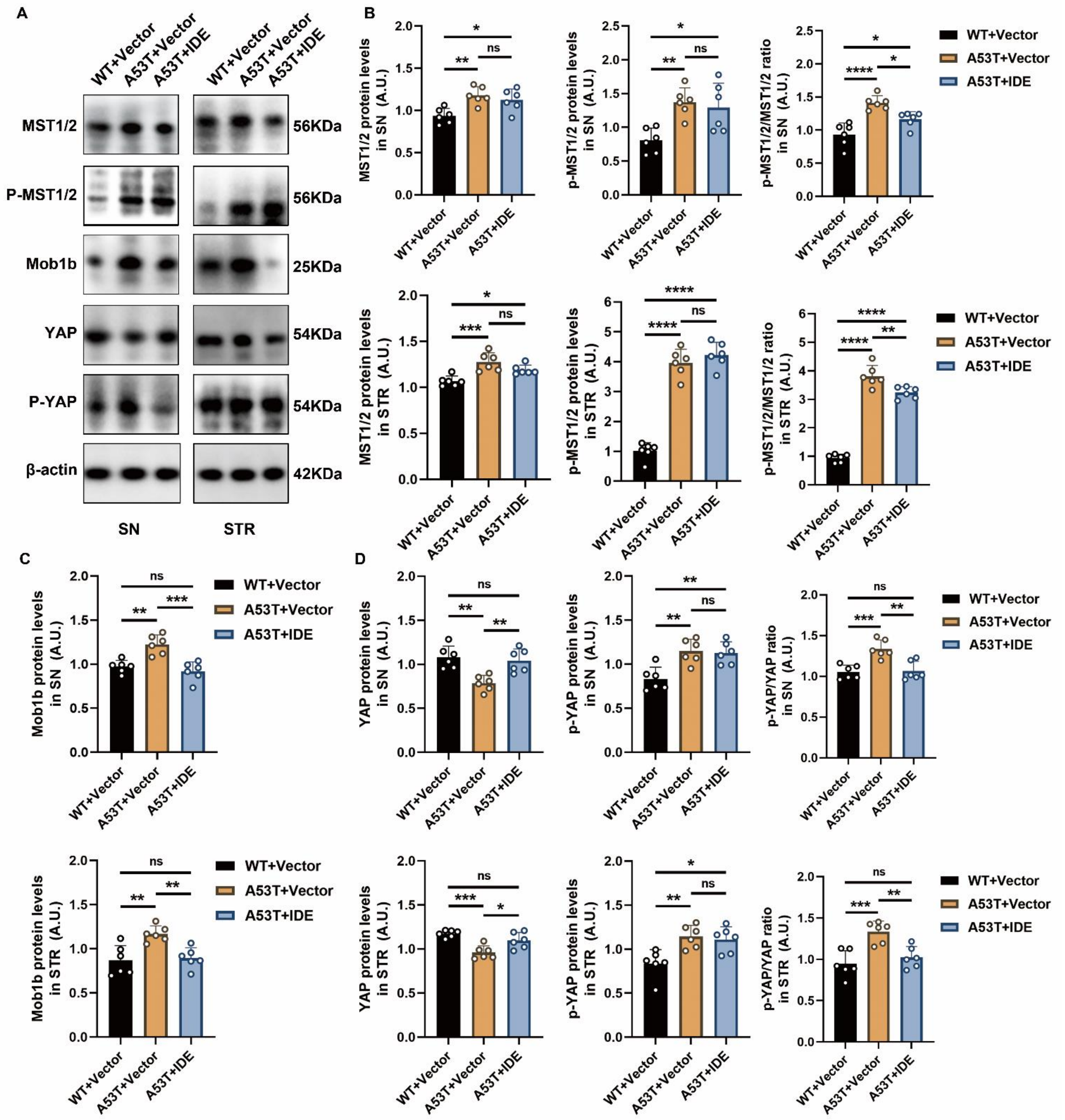
A
WT+Vector
A53T+Vector
A53T+IDE
MST1/2
P-MST1/2
Mob1b
YAP
P-YAP
β-actin
56KDa
56KDa
25KDa
54KDa
54KDa
42KDa
SN
STR
B
MST1/2 protein levels in SN (A.U.)
p-MST1/2 protein levels in SN (A.U.)
p-MST1/2/MST1/2 ratio in SN (A.U.)
MST1/2 protein levels in STR (A.U.)
p-MST1/2 protein levels in STR (A.U.)
p-MST1/2/MST1/2 ratio in STR (A.U.)
C
Mob1b protein levels in SN (A.U.)
Mob1b protein levels in STR (A.U.)
D
YAP protein levels in SN (A.U.)
p-YAP protein levels in SN (A.U.)
p-YAP/YAP ratio in SN (A.U.)
YAP protein levels in STR (A.U.)
p-YAP protein levels in STR (A.U.)
p-YAP/YAP ratio in STR (A.U.)
WT+Vector
A53T+Vector
A53T+IDE
ns
*
**
***
****

Fig. S6 Suppressive effects of IDE on the Hippo signaling pathways in A53T α-syn mice. (A) Representative western blot bands of MST1/2, p-MST1/2, Mob1b, YAP, and p-YAP in the SN and STR of WT+Vector, A53T+Vector, and A53T+IDE mice (n=6 per group). (B-D) The corresponding quantitative analyses of target proteins were performed (n=6 per group). ***** $P < 0.0001$, *** $P < 0.001$, ** $P < 0.01$, * $P < 0.05$, ns, not significant; ANOVA analyses and Tukey's test for post hoc comparisons.*

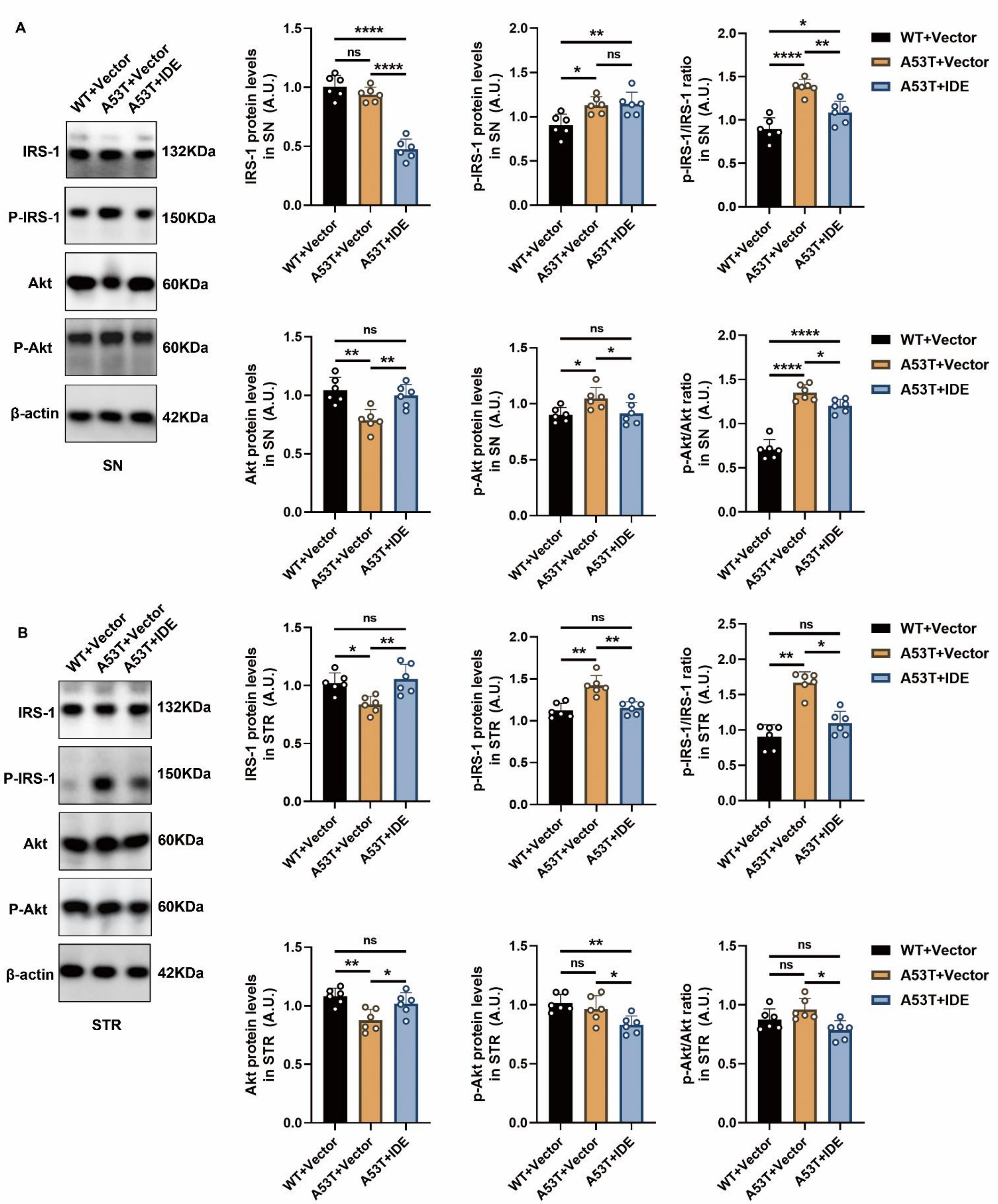
A
WT+Vector
A53T+Vector
A53T+IDE
IRS-1
132KDa
P-IRS-1
150KDa
Akt
60KDa
P-Akt
60KDa
β-actin
42KDa
SN
IRS-1 protein levels in SN (A.U.)
p-IRS-1 protein levels in SN (A.U.)
p-IRS-1/IRS-1 ratio in SN (A.U.)
Akt protein levels in SN (A.U.)
p-Akt protein levels in SN (A.U.)
p-Akt/Akt ratio in SN (A.U.)
B
STR
IRS-1 protein levels in STR (A.U.)
p-IRS-1 protein levels in STR (A.U.)
p-IRS-1/IRS-1 ratio in STR (A.U.)
Akt protein levels in STR (A.U.)
p-Akt protein levels in STR (A.U.)
p-Akt/Akt ratio in STR (A.U.)

Fig. S7 Inhibitive effects of IDE on the insulin signaling pathways in A53T α-syn mice. (A) The representative western blot bands and the corresponding quantitative analyses of IRS-1, p-IRS-1, Akt, and p-Akt about the SN of WT+Vector, A53T+Vector, and A53T+IDE mice (n=6 per group). ***** $P < 0.0001$, ** $P < 0.01$, * $P < 0.05$, ns, not significant; ANOVA analyses and Tukey's test for post hoc comparisons.* (B) The representative western blot bands and the corresponding quantitative analyses of IRS-1, p-IRS-1, Akt, and p-Akt in the STR of WT+Vector, A53T+Vector, and A53T+IDE mice (n=6 per group). *** $P < 0.01$, * $P < 0.05$, ns, not significant; ANOVA analyses and Tukey's test for post hoc comparisons.*

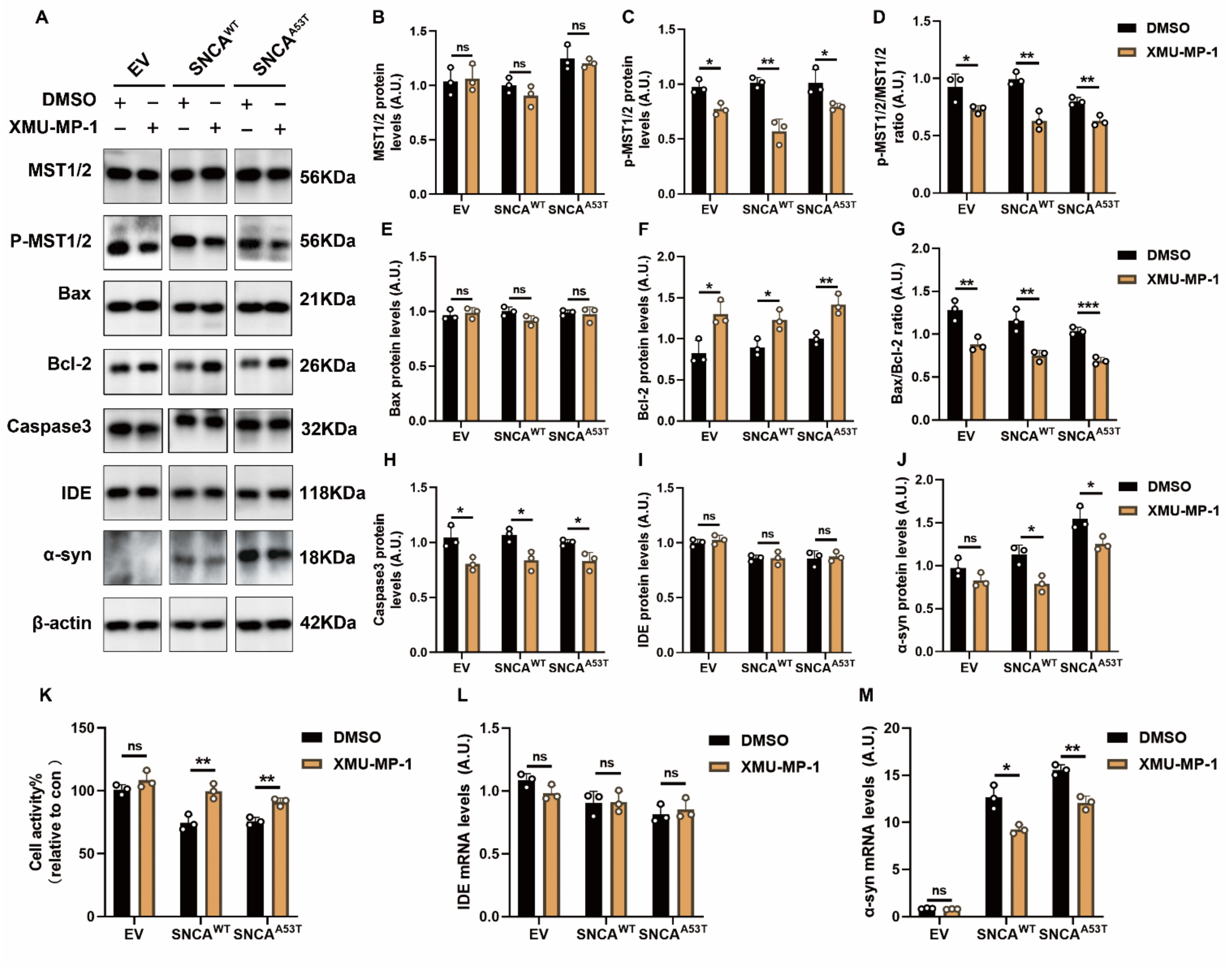

A
EV
SNCA WT
SNCA A53T
DMSO
XMU-MP-1
MST1/2
P-MST1/2
Bax
Bcl-2
Caspase3
IDE
α-syn
β-actin
56KDa
56KDa
21KDa
26KDa
32KDa
118KDa
18KDa
42KDa
B
MST1/2 protein levels (A.U.)
C
p-MST1/2 protein levels (A.U.)
D
p-MST1/2/MST1/2 ratio (A.U.)
E
Bax protein levels (A.U.)
F
Bcl-2 protein levels (A.U.)
G
Bax/Bcl-2 ratio (A.U.)
H
Caspase3 protein levels (A.U.)
I
IDE protein levels (A.U.)
J
α-syn protein levels (A.U.)
K
Cell activity% （relative to con ）
L
IDE mRNA levels (A.U.)
M
α-syn mRNA levels (A.U.)

Fig. S8 Critical role of MST1/2 for inhibiting Hippo signaling pathways in response to increased α-syn and neuronal apoptosis. PD model cells were pretreated with XMU-MP-1 (5 μM) or DMSO (Vehicle) for 24h to implement the assay. (A-D) Representative western blot bands of MST1/2 and p-MST1/2. The corresponding quantitative analyses of target proteins were performed (n=3, representing three independent experiments). ** *P < 0.01,* * *P < 0.05, ns, not significant; Student's t-test.* (A and E-H) Representative western blot bands of Bax, Bcl-2, and Caspase 3 and the corresponding quantitative analyses of target proteins were displayed (n=3, representing three independent experiments). *** *P < 0.001,* ** *P < 0.01,* * *P < 0.05, ns, not significant; Student's t-test.* (A, I, and J) Representative western blot bands and the quantitative analyses of IDE and α-syn (n=3, representing three independent experiments). * *P < 0.05, ns, not significant; Student's t-test.* (K) Cell viability was identified by CCK-8 assays (n=3, representing three independent experiments). ** *P < 0.01, ns, not significant; Student's t-test.* (L, M) Quantitative transcription analyses of IDE and α-syn (n=3, representing three independent experiments). ** *P < 0.01,* * *P < 0.05, ns, not significant; Student's t-test.*

Supplementary Tables

Table S1. Antibodies used in this study.

Target	Host	Supplier	Catalog number	WB Dilution	IF/IHC Dilution
IDE	Rabbit polyclonal	Abclonal	A11190	1:1000	-
IDE	Mouse monoclonal	Santa Cruz	sc-393887	-	1:500
α-synuclein	Rabbit polyclonal	Genetex	GTX112799	1:1500	1:1000
Myc Tag	Mouse monoclonal	Cell Signaling	2276S	1:1000	-
DYKDDDDK Tag	Rabbit monoclonal	Cell Signaling	14793S	1:1000	-
Akt	Rabbit monoclonal	Cell Signaling	4691S	1:1000	-
p-Akt (Ser473)	Rabbit monoclonal	Cell Signaling	4060S	1:2000	-
TH	Rabbit polyclonal	Proteintech	25859-1-AP	1:5000	1:2500
α-synuclein	Rabbit monoclonal	Abcam	ab138501	-	1:500
p-α-synuclein	Rabbit monoclonal	Abcam	ab51253	-	1:2500
MST1/2	Rabbit polyclonal	Proteintech	22245-1-AP	1:2000	-
p-MST1 (Thr183)/MST2 (Thr180)	Rabbit polyclonal	Proteintech	80093-1-RR	1:2000	-

Mob1a/b	Rabbit polyclonal	Abclonal	A18246	1:500	-
YAP	Rabbit polyclonal	Affinity	AF6328	1:500	-
p-YAP (Ser127)	Rabbit polyclonal	Affinity	AF3328	1:500	-
IRS-1	Rabbit polyclonal	Proteintech	17509-1-AP	1:500	-
p-IRS-1 (Ser636)	Rabbit polyclonal	ZenBio	251484	1:500	-
Bax	Rabbit polyclonal	Proteintech	50599-2-Ig	1:2000	-
Bcl-2	Mouse monoclonal	Proteintech	66799-1-Ig	1:1000	-
Caspase 3	Rabbit polyclonal	Proteintech	19677-1-AP	1:1000	-
β-actin	Mouse monoclonal	Proteintech	66009-1-Ig	1:10000	-
GAPDH	Rabbit monoclonal	Cell Signaling	5174S	1:1000	-

Table S2. Primer and siRNA used in this study.

Application	Primer/ siRNA name	Primer sequence
qRT-PCR in mice	IDE-F	5’-AATCCGGCCATCCAGAGAATA-3’
	IDE-R	5’-GGGTCTGACAGTGAACCTATGT-3’
	SNCA-F	5’-GGGAGTCCTCTATGTAGGTTCC-3’
	SNCA-R	5’-TTCATTGGCTACACCTTGACCTAA-3’
	GAPDH-F	5’-TGGAAAGCTGTGGCGTGATG-3’
	GAPDH-R	5’-TACTTGGCAGGTTTCTCCAGG-3’
qRT-PCR in SH-SY5Ycells	IDE-F	5’-TTTTCAGCCCATTTGCTTATGTG-3’
	IDE-R	5’-TGCATACTCGTTGAGTGAGTCTT-3’
	SNCA-F	5’-AAGAGGGTGTTCTCTATGTAGGC-3’
	SNCA-R	5’-GCTCCTCCAACATTTGTCACTT-3’
	GAPDH-F	5’-GGTGTGAACCATGAGAAGTATGA-3’
	GAPDH-R	5’-GAGTCCTTCCACGATACCAAAG-3’
	β-actin-F	5’-GAGAAAATCTGGCACCACACC-3’

	β-actin-R	5’-GGATAGCACAGCCTGGATAGCAA-3’
siRNA in SH-SY5Ycells	IDE-siRNA-F	5’-CAGAAAUACUACAAAUCAAAUTT-3’
	IDE-siRNA-R	5’-AUUUGAUUUGUAGUAUUUCUGTT-3’
	NC-F	5’-UUCUCCGAACGUGUCACGUTT-3’
	NC-R	5’-ACGUGACACGUUCGGAGAATT-3’

Table S3. Differentially expressed genes in the transcriptome of the SN and STR of A53T+IDE/A53T+Vector mice.

NO.	Gene symbol	Log2(FC)	p-value	SN Type	Gene symbol	Log2(FC)	p-value	STR Type
1	Gm45062	13.868	0.000000	Up	Gm45062	16.130	0.000000	Up
2	Try4	13.502	0.000000	Up	Gm20521	15.084	0.000000	Up
3	Gm20390	13.271	0.000000	Up	Igkv3-10	13.818	0.000000	Up
4	Cpa1	12.689	0.000000	Up	Sult1c1	13.308	0.019747	Up
5	S100a5	11.879	0.012522	Up	S100a8	12.513	0.000047	Up
6	Lep	11.870	0.000000	Up	Gm20422	12.321	0.000000	Up
7	Gm3030	11.826	0.007810	Up	Gm48827	11.957	0.013275	Up
8	Gm12308	11.269	0.023377	Up	Dpep1	11.940	0.031477	Up
9	Gm13184	10.950	0.027250	Up	Gpihbp1	11.850	0.031928	Up
10	Gm49368	10.903	0.000000	Up	Frmpd1os	11.781	0.015594	Up
11	Lipf	10.892	0.008872	Up	Wfdc6a	11.344	0.003710	Up

12	1700042O10Rik	10.863	0.000745	Up	Gm46560	11.019	0.006396	Up
13	4930545L08Rik	10.778	0.047815	Up	Hao1	10.986	0.000024	Up
14	Gm45842	10.642	0.010838	Up	H1f5	10.810	0.006704	Up
15	Gm50008	10.593	0.004263	Up	Gm11734	10.733	0.030373	Up
16	1700102H20Rik	10.578	0.038084	Up	Gm16150	10.686	0.015644	Up
17	Gm29570	10.555	0.021269	Up	4931431B13Rik	10.622	0.000754	Up
18	Gm8010	10.424	0.031433	Up	Spata46	10.575	0.009818	Up
19	Gm21983	10.150	0.006998	Up	Serpina3i	10.481	0.000121	Up
20	4930408O17Rik	10.137	0.003824	Up	Gm12267	10.476	0.045852	Up
21	Mroh6	9.966	0.000504	Up	Gm1848	10.466	0.011656	Up
22	Gm10165	9.964	0.000205	Up	Wfdc12	10.431	0.045982	Up
23	4931406G06Rik	9.812	0.041177	Up	Gm33707	10.424	0.047175	Up
24	Gm37710	9.768	0.015087	Up	Bcl2a1d	10.406	0.037193	Up
25	Gm9067	9.571	0.023688	Up	Cyp3a57	10.375	0.008386	Up

26	Rgs13	9.559	0.027778	Up	Chil3	10.365	0.004919	Up
27	Gm37023	9.559	0.023924	Up	Tex35	10.350	0.017665	Up
28	Gm6377	9.528	0.000604	Up	Gm15810	10.316	0.028723	Up
29	Slco6c1	9.486	0.001204	Up	Gm31107	10.273	0.041819	Up
30	E330034G19Rik	9.450	0.007611	Up	Gm29460	10.224	0.031850	Up
31	Gsg1	9.391	0.037754	Up	Ren1	10.211	0.005731	Up
32	Gm45336	9.383	0.000809	Up	Gm26943	10.207	0.035170	Up
33	Krt6a	9.369	0.003114	Up	4930551O13Rik	10.185	0.026728	Up
34	Klk1b5	9.153	0.049416	Up	Asgr1	10.182	0.001542	Up
35	Nxf2	9.137	0.015800	Up	Adad2	10.172	0.000658	Up
36	C86187	9.112	0.022222	Up	Lmntd2	10.138	0.000225	Up
37	Mpig6b	8.979	0.011596	Up	Slc5a10	10.041	0.003442	Up
38	Gm26911	8.806	0.013456	Up	1700034G24Rik	9.988	0.013468	Up
39	Rhag	8.805	0.043048	Up	4933412L11Rik	9.953	0.018145	Up

40	Gm16223	8.797	0.041501	Up	Gm26793	9.925	0.000555	Up
41	Platr6	8.782	0.042799	Up	Gm47715	9.912	0.039646	Up
42	A230028O05Rik	8.781	0.009330	Up	Soat2	9.890	0.017842	Up
43	Gm43670	8.773	0.046041	Up	Gm32219	9.874	0.001552	Up
44	Gm37606	8.742	0.006504	Up	Gm6525	9.864	0.024233	Up
45	Gm37109	8.558	0.033486	Up	1700020N01Rik	9.855	0.046226	Up
46	Gm49069	8.405	0.007175	Up	8430419K02Rik	9.855	0.012491	Up
47	9630028H03Rik	8.383	0.010579	Up	4931413K12Rik	9.845	0.029992	Up
48	Gm7697	8.355	0.039875	Up	Mroh3	9.818	0.000044	Up
49	Gm30524	8.352	0.049257	Up	Oit1	9.690	0.025763	Up
50	Gm28651	8.263	0.032241	Up	Tcf21	9.679	0.006994	Up
51	4921516A02Rik	8.258	0.048097	Up	D530018E20Rik	9.674	0.038501	Up
52	4921528I07Rik	8.139	0.012381	Up	Gm32369	9.637	0.030959	Up
53	4930455M05Rik	8.101	0.015804	Up	Gm19142	9.608	0.030739	Up

54	Dcstamp	8.040	0.041322	Up	Fam243	9.547	0.006518	Up
55	Slc24a1	7.830	0.014372	Up	Sytl3	9.532	0.000061	Up
56	Ctrb1	7.488	0.002997	Up	Gm45129	9.415	0.004637	Up
57	Ugt1a6b	5.808	0.000290	Up	Phox2a	9.413	0.007895	Up
58	Gm7867	4.872	0.035193	Up	Gm45117	9.261	0.017328	Up
59	Csf3	4.646	0.010534	Up	Ces2e	9.204	0.040171	Up
60	Gm29050	4.630	0.025660	Up	Gm38319	9.201	0.048466	Up
61	Amy2a3	4.440	0.002304	Up	4930471M09Rik	9.189	0.000555	Up
62	Amy2a5	4.329	0.009673	Up	C2cd4a	9.117	0.047302	Up
63	Uts2	4.314	0.000273	Up	Gm5608	9.111	0.008099	Up
64	Spata48	3.964	0.010049	Up	2310015D24Rik	9.099	0.001814	Up
65	Cxcr5	3.892	0.042398	Up	Gm7697	9.081	0.022782	Up
66	Amy2a4	3.860	0.013403	Up	Gm48878	9.067	0.005699	Up
67	Trem1	3.765	0.038658	Up	Olfr1507	9.015	0.012589	Up

68	Plekhs1	3.665	0.030286	Up	Tspan1	8.999	0.030524	Up
69	Gm19585	3.632	0.045071	Up	Cyp1a1	8.980	0.023212	Up
70	Amy2a2	3.617	0.000024	Up	Lhx3	8.965	0.004280	Up
71	Gm12996	3.546	0.049859	Up	Gm37079	8.949	0.017984	Up
72	Selp	3.469	0.018353	Up	Gm4952	8.944	0.033855	Up
73	Tspo2	3.347	0.034331	Up	9130227L01Rik	8.896	0.034874	Up
74	Gm49749	3.241	0.020456	Up	1300017J02Rik	8.889	0.005716	Up
75	Aire	3.208	0.008604	Up	Gm44260	8.864	0.026096	Up
76	Gm9292	3.157	0.012664	Up	Vmn2r-ps45	8.790	0.046029	Up
77	Gprc5d	3.008	0.000448	Up	Gm43320	8.770	0.008986	Up
78	Gm45717	2.989	0.000237	Up	Gm33148	8.685	0.000332	Up
79	Clec4d	2.961	0.048369	Up	Adam5	8.684	0.027602	Up
80	Myl3	2.930	0.003359	Up	Gm5087	8.683	0.031674	Up
81	Gm10384	2.907	0.043255	Up	Synb	8.550	0.003777	Up

82	Uts2b	2.761	0.008602	Up	Gm16291	8.510	0.026547	Up
83	Emilin3	2.589	0.041379	Up	Olfr221	8.447	0.017731	Up
84	Spef1l	2.453	0.020744	Up	Bglap3	8.405	0.034468	Up
85	Adipoq	2.412	0.012122	Up	Snhg7os	8.257	0.029523	Up
86	Slc10a6	2.394	0.008194	Up	Gm27179	8.225	0.026414	Up
87	Sele	2.386	0.000635	Up	Lrrc17	8.168	0.014852	Up
88	Gm12840	2.376	0.022772	Up	Gm16041	8.098	0.018160	Up
89	Scgn	2.363	0.019198	Up	Slfn4	8.090	0.046575	Up
90	Lta	2.362	0.035819	Up	Gm36199	8.087	0.029639	Up
91	Gm21982	2.350	0.032663	Up	Samd7	8.019	0.016365	Up
92	Gm11738	2.304	0.028143	Up	Slc12a1	7.998	0.017678	Up
93	Cd14	2.295	0.040057	Up	Csf3	7.728	0.003704	Up
94	Bpifb1	2.289	0.008841	Up	Krt7	7.668	0.004849	Up
95	Gp9	2.249	0.029992	Up	Gm36640	7.529	0.044628	Up

96	Adgrg3	2.158	0.018238	Up	Speer4a	7.512	0.003000	Up
97	Gm49527	2.149	0.000000	Up	Veph1	6.956	0.033588	Up
98	Adamts9	2.139	0.030389	Up	Adamts12	6.612	0.019507	Up
99	Gm15533	2.120	0.044520	Up	Gm884	6.610	0.019337	Up
100	Fcer2a	1.968	0.016875	Up	Gm12904	6.554	0.004956	Up
101	Gm20692	1.942	0.022755	Up	Cltrn	5.814	0.000700	Up
102	Rpl26	1.836	0.029719	Up	Gm49932	5.670	0.001239	Up
103	Skida1	1.824	0.014807	Up	Slc4a5	5.563	0.000064	Up
104	Pilrb2	1.790	0.047620	Up	Aqp1	5.384	0.000001	Up
105	Hcar2	1.702	0.034642	Up	Gm49774	5.252	0.005174	Up
106	Gm19951	1.666	0.020544	Up	Gm12840	5.201	0.000299	Up
107	Itgb1bp2	1.609	0.021657	Up	Serpina3f	5.168	0.046504	Up
108	Nkx6-3	1.586	0.022502	Up	Ttr	5.166	0.000173	Up
109	Tph2	1.578	0.027839	Up	Dntt	5.079	0.032976	Up

110	Gm49179	1.571	0.000908	Up	Emilin3	5.058	0.040741	Up
111	Cldn18	1.548	0.037073	Up	Fam24a	5.005	0.017612	Up
112	Gm48045	1.546	0.026680	Up	Wfikkn2	4.983	0.000413	Up
113	Gm9169	1.543	0.033743	Up	Tmem72	4.982	0.000280	Up
114	Slc6a4	1.540	0.027077	Up	Cdh3	4.878	0.000185	Up
115	Dmrta1	1.491	0.006654	Up	Tex45	4.849	0.002580	Up
116	Gm43442	1.483	0.018743	Up	Gm31036	4.748	0.037693	Up
117	Gm49395	1.467	0.004115	Up	Lmx1a	4.686	0.000126	Up
118	S100a9	1.466	0.008570	Up	Kcnj13	4.678	0.000147	Up
119	Cidec	1.412	0.026487	Up	Folr1	4.450	0.000646	Up
120	Gm47135	1.383	0.045970	Up	Gm21284	4.450	0.002087	Up
121	Tnfrsf4	1.371	0.021355	Up	Zscan10	4.411	0.010678	Up
122	Vsig8	1.365	0.017348	Up	Clcnka	4.340	0.012017	Up
123	Marchf10	1.359	0.042705	Up	Gm26783	4.313	0.002796	Up

124	Tnnt2	1.355	0.019835	Up	Cldn2	4.244	0.001225	Up
125	Fhl4	1.347	0.031999	Up	Slc24a1	4.237	0.016133	Up
126	Cd300lf	1.341	0.002959	Up	Mfrp	4.227	0.000613	Up
127	Gm49392	1.313	0.049825	Up	CT868690	4.227	0.044279	Up
128	Psca	1.288	0.010278	Up	Gm42957	4.210	0.003431	Up
129	Cblif	1.278	0.026876	Up	Kcne2	4.204	0.003472	Up
130	Atp4b	1.263	0.031938	Up	Steap1	4.187	0.001956	Up
131	Trip13	1.255	0.015245	Up	Gm37407	3.963	0.027757	Up
132	Gm42303	1.247	0.017399	Up	H2-T-ps	3.897	0.033186	Up
133	Cxcl1	1.245	0.013566	Up	Cym	3.894	0.007929	Up
134	Gm13068	1.226	0.030737	Up	F5	3.880	0.001814	Up
135	Gm44850	1.206	0.024542	Up	Btnl9	3.874	0.041602	Up
136	1810020O05Rik	1.198	0.003351	Up	Igfals	3.866	0.013195	Up
137	Tgm4	1.186	0.038056	Up	Ecrg4	3.832	0.000463	Up

138	Gchfr	1.169	0.000148	Up	2900040C04Rik	3.832	0.000002	Up
139	Gm35025	1.168	0.035322	Up	Oca2	3.803	0.003961	Up
140	Gm38560	1.156	0.037273	Up	Hnrnpa3	3.793	0.000178	Up
141	Fev	1.155	0.009430	Up	Atp6v0a4	3.743	0.032814	Up
142	Itgad	1.146	0.036519	Up	Prr15l	3.721	0.040486	Up
143	Pcdhgb8	1.141	0.010890	Up	Col8a1	3.663	0.000573	Up
144	Shld3	1.139	0.003116	Up	Gm21982	3.657	0.000125	Up
145	Apol6	1.138	0.014725	Up	Wdr86	3.643	0.004517	Up
146	Mucl3	1.130	0.045757	Up	Krt18	3.557	0.000823	Up
147	Phox2b	1.125	0.044962	Up	Tmprss11a	3.462	0.005656	Up
148	Gm4675	1.121	0.040185	Up	Cplx4	3.451	0.038146	Up
149	Gm26783	1.121	0.038377	Up	Gm14120	3.408	0.006408	Up
150	Slc18a3	1.118	0.001895	Up	Kl	3.402	0.004088	Up
151	Gm44386	1.115	0.031300	Up	Itgb6	3.390	0.037209	Up

152	Npb	1.110	0.031604	Up	Gm28040	3.375	0.010122	Up
153	Ncaph	1.092	0.035259	Up	Spdef	3.362	0.000252	Up
154	Gm10109	1.087	0.048654	Up	Prlr	3.318	0.000524	Up
155	Ap3s1-ps1	1.077	0.003386	Up	1700093K21Rik	3.313	0.002629	Up
156	Tgm1	1.065	0.015207	Up	Sostdc1	3.299	0.010262	Up
157	Evx2	1.060	0.003334	Up	Gm9316	3.252	0.039566	Up
158	Pycr1	1.055	0.020448	Up	Rrh	3.250	0.006336	Up
159	Olfr1344	1.039	0.009712	Up	Gm534	3.250	0.001453	Up
160	Duxbl1	1.031	0.025718	Up	Gm38220	3.233	0.020543	Up
161	Slc5a7	1.023	0.012959	Up	Slc13a4	3.141	0.003349	Up
162	Zfp185	-1.002	0.029242	Down	Slc10a6	3.131	0.009533	Up
163	Ptger3	-1.002	0.011342	Down	Slc2a12	3.103	0.001806	Up
164	Glt8d2	-1.005	0.028598	Down	Gm49686	3.092	0.020741	Up
165	Prelp	-1.009	0.000000	Down	Gm26813	3.082	0.002919	Up

166	Pla2r1	-1.009	0.037004	Down	A2m	3.045	0.004788	Up
167	Loxl2	-1.010	0.005017	Down	Il17re	3.045	0.001233	Up
168	Sphk1	-1.012	0.000580	Down	Six1	3.012	0.048712	Up
169	Phldb2	-1.012	0.000014	Down	Sapcd1	2.988	0.004397	Up
170	Slfn9	-1.013	0.032880	Down	Amy2a2	2.982	0.029330	Up
171	Anxa4	-1.013	0.000916	Down	Abca4	2.977	0.003270	Up
172	Gm10676	-1.014	0.024771	Down	Gm9495	2.948	0.035303	Up
173	Ppp1r3b	-1.019	0.000987	Down	Gm11681	2.948	0.041537	Up
174	Ajuba	-1.023	0.002965	Down	Lcn2	2.944	0.025327	Up
175	Gm10243	-1.028	0.024929	Down	Slc16a8	2.921	0.004478	Up
176	Pawr	-1.029	0.001752	Down	Gm42596	2.920	0.038555	Up
177	Isyna1	-1.030	0.000000	Down	Socs3	2.884	0.021900	Up
178	Gm44168	-1.030	0.002696	Down	Gm50367	2.871	0.041660	Up
179	Gbp6	-1.031	0.005227	Down	Il1b	2.870	0.017862	Up

180	Kcnj4	-1.034	0.037039	Down	Adamts9	2.841	0.020781	Up
181	Slco2a1	-1.034	0.006314	Down	B4galnt3	2.835	0.001000	Up
182	Barhl2	-1.035	0.023887	Down	Prr32	2.806	0.041389	Up
183	Gm5345	-1.037	0.039716	Down	Msx3	2.803	0.040447	Up
184	Ccdc8	-1.040	0.001717	Down	Krt23	2.760	0.014258	Up
185	Pde5a	-1.042	0.000001	Down	Gm16201	2.723	0.001285	Up
186	Gpx8	-1.047	0.001442	Down	Col4a4	2.705	0.000002	Up
187	Plekha4	-1.049	0.026219	Down	Ccl12	2.687	0.008058	Up
188	Col13a1	-1.051	0.024556	Down	Dmrt3	2.685	0.022802	Up
189	Gm28042	-1.060	0.015408	Down	Ackr4	2.679	0.000975	Up
190	Cavin1	-1.060	0.000000	Down	Slc9b1	2.665	0.031001	Up
191	Gm28551	-1.061	0.010700	Down	1600029O15Rik	2.633	0.000348	Up
192	Hoxaas2	-1.062	0.035825	Down	Thbs1	2.625	0.016224	Up
193	Tmem200b	-1.068	0.000088	Down	Epn3	2.622	0.008513	Up

194	Ifi203	-1.069	0.041872	Down	Dmgdh	2.597	0.000088	Up
195	Serpind1	-1.073	0.000003	Down	Cldn1	2.592	0.001032	Up
196	Gm15675	-1.075	0.043341	Down	Wdr72	2.588	0.018483	Up
197	Jchain	-1.081	0.000936	Down	Sema3b	2.588	0.001456	Up
198	Rab7b	-1.083	0.013079	Down	Tmco5b	2.579	0.025611	Up
199	Foxl2os	-1.083	0.030198	Down	Lbp	2.564	0.014010	Up
200	Sned1	-1.085	0.000000	Down	Gm47370	2.548	0.007259	Up
201	Pkn3	-1.085	0.000741	Down	Ephx3	2.541	0.025640	Up
202	Itga11	-1.094	0.007161	Down	Atp2c2	2.512	0.004615	Up
203	Cbr2	-1.099	0.002116	Down	P2ry2	2.509	0.001922	Up
204	Gm48678	-1.100	0.004568	Down	Gm29674	2.508	0.035584	Up
205	Abca4	-1.101	0.012294	Down	Slc6a5	2.471	0.048594	Up
206	Gypc	-1.104	0.000533	Down	Exoc3l2	2.467	0.037024	Up
207	Pamr1	-1.105	0.000333	Down	Gm31406	2.440	0.044334	Up

208	Tert	-1.105	0.041604	Down	Enpp2	2.439	0.001073	Up
209	Tgtp2	-1.106	0.036145	Down	Rab20	2.419	0.002274	Up
210	Clec3b	-1.107	0.000921	Down	Gm7972	2.396	0.005786	Up
211	Gm7666	-1.108	0.000878	Down	Mymk	2.394	0.042454	Up
212	Stra6	-1.114	0.000000	Down	Zfp185	2.391	0.000104	Up
213	Gjb2	-1.115	0.000007	Down	Col8a2	2.376	0.029472	Up
214	F13a1	-1.115	0.000001	Down	Gmnc	2.372	0.002302	Up
215	Gstm2	-1.116	0.001523	Down	Gm15904	2.365	0.021273	Up
216	Foxc1	-1.116	0.000043	Down	Tmem252	2.353	0.039135	Up
217	Cenpu	-1.127	0.040105	Down	Sele	2.339	0.018517	Up
218	H2-Aa	-1.127	0.001445	Down	Gm43412	2.334	0.016730	Up
219	Iigp1	-1.130	0.009395	Down	Otx2	2.325	0.043440	Up
220	Arhgap28	-1.132	0.010918	Down	Gm11738	2.318	0.046855	Up
221	Col6a2	-1.135	0.000000	Down	Rps18-ps6	2.313	0.013262	Up

222	Serping1	-1.136	0.000006	Down	Adamts14	2.307	0.001267	Up
223	Sec16b	-1.143	0.017113	Down	Lrat	2.307	0.012856	Up
224	Efemp1	-1.143	0.000000	Down	Gm6402	2.301	0.000013	Up
225	Colec12	-1.146	0.000000	Down	Krt8	2.279	0.004217	Up
226	Msx1	-1.148	0.012627	Down	Gm47173	2.269	0.001231	Up
227	Cytl1	-1.155	0.019508	Down	Cldn3	2.268	0.027817	Up
228	Fgl2	-1.161	0.000002	Down	Gm13344	2.268	0.028966	Up
229	Emp3	-1.165	0.000142	Down	Tent5b	2.244	0.001914	Up
230	Bmp4	-1.166	0.000084	Down	Fap	2.240	0.014984	Up
231	Ddr2	-1.170	0.000000	Down	Ch25h	2.211	0.026195	Up
232	Aspn	-1.174	0.001066	Down	Steap4	2.210	0.002021	Up
233	Cyp1b1	-1.174	0.000015	Down	H2-Q5	2.209	0.025421	Up
234	Rbp1	-1.177	0.000000	Down	Col9a3	2.202	0.004687	Up
235	Frk	-1.180	0.040341	Down	Gm43636	2.158	0.044170	Up

236	Hic1	-1.185	0.000328	Down	Ndor1	2.149	0.014864	Up
237	Insrr	-1.188	0.008377	Down	Scara5	2.129	0.001521	Up
238	Angptl1	-1.193	0.022334	Down	Gm44836	2.128	0.043122	Up
239	Lum	-1.195	0.000003	Down	Trpv4	2.114	0.007995	Up
240	Slitrk6	-1.205	0.007073	Down	Gm47202	2.099	0.023370	Up
241	Adam12	-1.206	0.000030	Down	Pon3	2.077	0.000031	Up
242	Slc22a8	-1.207	0.000003	Down	Wnt11	2.062	0.017204	Up
243	Cps1	-1.208	0.020313	Down	Serpina3g	2.058	0.007298	Up
244	Slc13a3	-1.209	0.000000	Down	Synpo2l	2.056	0.028459	Up
245	Slc7a11	-1.209	0.000000	Down	Rdh5	2.056	0.004915	Up
246	Rcn3	-1.211	0.000000	Down	Ceacam10	2.053	0.036565	Up
247	C2	-1.217	0.000002	Down	Gm44250	2.035	0.013847	Up
248	Aoc3	-1.218	0.000058	Down	Frem1	2.033	0.017768	Up
249	Stap2	-1.220	0.001180	Down	Gm20687	2.014	0.000107	Up

250	Zfp385c	-1.225	0.011866	Down	Cxcl1	1.996	0.020754	Up
251	Gm17200	-1.231	0.031043	Down	Omp	1.992	0.034544	Up
252	C1qtnf2	-1.232	0.010584	Down	Tfap2a	1.970	0.027386	Up
253	Mrc1	-1.238	0.000020	Down	Igfbp2	1.951	0.008462	Up
254	Clec4a3	-1.242	0.029185	Down	Angptl4	1.943	0.019167	Up
255	Twist1	-1.258	0.000480	Down	2600014E21Rik	1.941	0.012804	Up
256	Ptgfr	-1.265	0.005206	Down	Gm42462	1.934	0.036318	Up
257	Pcdha9	-1.268	0.000012	Down	Cd300lf	1.933	0.003363	Up
258	Lmod1	-1.268	0.040120	Down	Glb1l2	1.922	0.008870	Up
259	Itga10	-1.271	0.000021	Down	Slc37a2	1.912	0.008243	Up
260	Irf6	-1.278	0.005220	Down	Tnfrsf10b	1.907	0.005708	Up
261	Bmp7	-1.281	0.000000	Down	Col4a3	1.873	0.001139	Up
262	Mgp	-1.285	0.037356	Down	Rbm47	1.867	0.012592	Up
263	Wnt4	-1.302	0.000272	Down	Gm44117	1.867	0.009362	Up

264	Gpr20	-1.306	0.005262	Down	Ace	1.861	0.003212	Up
265	Col8a2	-1.307	0.000054	Down	Gm47096	1.853	0.006791	Up
266	Cybb	-1.309	0.000242	Down	1700112J16Rik	1.842	0.016331	Up
267	Il17re	-1.315	0.028094	Down	Tcf23	1.828	0.015140	Up
268	Emilin1	-1.327	0.000063	Down	Akr1c18	1.814	0.002660	Up
269	Dapl1	-1.332	0.031333	Down	Gm4544	1.814	0.013207	Up
270	Crocc2	-1.335	0.026127	Down	Rad51b	1.814	0.023350	Up
271	Gbgt1	-1.340	0.018641	Down	Pycard	1.794	0.003540	Up
272	Pi15	-1.344	0.015885	Down	Gpx8	1.789	0.000208	Up
273	BE692007	-1.346	0.027399	Down	Slc39a4	1.786	0.002405	Up
274	Slc9a2	-1.349	0.000000	Down	Gm10705	1.785	0.003387	Up
275	Serpine1	-1.354	0.000146	Down	Large2	1.778	0.027310	Up
276	Thbd	-1.354	0.000000	Down	Ifi204	1.757	0.019521	Up
277	Gm26779	-1.359	0.029071	Down	Ada	1.757	0.002344	Up

278	Calhm5	-1.361	0.000012	Down	Bcl3	1.752	0.011109	Up
279	Bmp6	-1.367	0.000000	Down	Trpm3	1.727	0.017126	Up
280	Mrc2	-1.370	0.000000	Down	Scn10a	1.725	0.004675	Up
281	Ripor3	-1.374	0.011265	Down	Lepr	1.724	0.001976	Up
282	Foxl2	-1.375	0.000406	Down	Ccl17	1.719	0.029690	Up
283	Mrvi1	-1.383	0.000000	Down	Olfr920	1.719	0.045154	Up
284	Tgtp1	-1.385	0.009405	Down	Rps18-ps3	1.711	0.002069	Up
285	Elf3	-1.395	0.016337	Down	Gm29609	1.704	0.022617	Up
286	Sstr4	-1.396	0.031000	Down	Tmem37	1.699	0.013046	Up
287	Adamtsl3	-1.396	0.000000	Down	Gm43305	1.693	0.000014	Up
288	9630028B13Rik	-1.437	0.017087	Down	Acsm3	1.658	0.046286	Up
289	Wfikkn2	-1.438	0.000938	Down	Treml2	1.654	0.024564	Up
290	Fendrr	-1.440	0.016294	Down	Pla2g5	1.649	0.003697	Up
291	Rprm	-1.445	0.019842	Down	Gm17907	1.647	0.042008	Up

292	Plac8	-1.449	0.010617	Down	Gm26902	1.642	0.003982	Up
293	Cldn15	-1.457	0.049392	Down	Pla2g4e	1.637	0.000275	Up
294	Tspan11	-1.470	0.000450	Down	Ppp1r3b	1.636	0.022232	Up
295	Cped1	-1.482	0.000000	Down	Slc2a9	1.634	0.006861	Up
296	Prrx2	-1.482	0.001277	Down	Hyal3	1.632	0.036380	Up
297	Ccl5	-1.485	0.025132	Down	Gm20751	1.627	0.019252	Up
298	Slc6a20a	-1.486	0.016372	Down	Trim72	1.618	0.021807	Up
299	Igf2	-1.500	0.001881	Down	Gm38593	1.614	0.048568	Up
300	Cd209f	-1.500	0.021023	Down	Gm49864	1.606	0.049209	Up
301	Aebp1	-1.500	0.000000	Down	Gm26648	1.605	0.000691	Up
302	Six5	-1.510	0.000032	Down	Lrg1	1.590	0.002198	Up
303	Tbx18	-1.527	0.000004	Down	Cebpd	1.590	0.020018	Up
304	Smim5	-1.529	0.015695	Down	Gm47171	1.579	0.044544	Up
305	Igfbp6	-1.532	0.000000	Down	Col13a1	1.578	0.022113	Up

306	Gpr182	-1.541	0.000000	Down	Spag16	1.570	0.008290	Up
307	Chrm5	-1.549	0.000034	Down	Irx3	1.570	0.048793	Up
308	Lilra5	-1.554	0.036820	Down	Slc43a3	1.552	0.007269	Up
309	Gm6685	-1.557	0.007404	Down	Gm43720	1.550	0.044062	Up
310	Foxd1	-1.559	0.000007	Down	St6galnac2	1.550	0.012578	Up
311	Olfml2a	-1.564	0.000000	Down	Gm44770	1.549	0.041588	Up
312	Dusp27	-1.565	0.000713	Down	Gm35853	1.541	0.006875	Up
313	Gm43089	-1.571	0.030843	Down	Gm41361	1.539	0.044955	Up
314	Aldh1a2	-1.574	0.005460	Down	Fbn1	1.538	0.001227	Up
315	Card14	-1.575	0.004446	Down	Icam1	1.534	0.018976	Up
316	Foxd2	-1.582	0.003934	Down	Gm45774	1.531	0.005437	Up
317	Lhx6	-1.588	0.023341	Down	Odf4	1.526	0.011849	Up
318	Brms1	-1.591	0.047607	Down	Duxbl2	1.523	0.030744	Up
319	Moxd1	-1.607	0.000118	Down	Kif23	1.517	0.003951	Up

320	Zic4	-1.616	0.000000	Down	2700046A07Rik	1.513	0.003334	Up
321	Cdh1	-1.621	0.000000	Down	Rdh16f1	1.510	0.018029	Up
322	Krt80	-1.623	0.006978	Down	Il12rb1	1.504	0.001588	Up
323	Myo7b	-1.624	0.040941	Down	Cpxm2	1.496	0.005698	Up
324	Fbxw23	-1.624	0.000707	Down	Gm9768	1.489	0.008729	Up
325	Aoc1	-1.631	0.019731	Down	Calml4	1.489	0.010115	Up
326	Ly6m	-1.636	0.044798	Down	Crb3	1.483	0.019928	Up
327	H2-Q1	-1.637	0.000006	Down	Gm26684	1.481	0.004468	Up
328	Fbln1	-1.639	0.000000	Down	Slc29a4	1.474	0.046575	Up
329	Asgr1	-1.639	0.015226	Down	Slc6a20a	1.472	0.011763	Up
330	Omd	-1.648	0.001805	Down	Gm16534	1.471	0.008466	Up
331	Sim1	-1.659	0.001803	Down	Clic6	1.460	0.041317	Up
332	Nrk	-1.665	0.044750	Down	Tmem52	1.455	0.006117	Up
333	C1qtnf7	-1.665	0.000337	Down	Tmem232	1.454	0.033584	Up

334	Podn	-1.684	0.000015	Down	Steap2	1.443	0.031718	Up
335	Slc6a12	-1.695	0.032621	Down	Loxl2	1.443	0.018010	Up
336	Siglec1	-1.703	0.000360	Down	Myl4	1.438	0.000506	Up
337	Gm14824	-1.704	0.013040	Down	Gm38391	1.435	0.049845	Up
338	Mrgprf	-1.716	0.000055	Down	Tmem147os	1.422	0.026667	Up
339	Dpp4	-1.722	0.000100	Down	Gm10591	1.414	0.000652	Up
340	B230323A14Rik	-1.724	0.000216	Down	Edar	1.402	0.037084	Up
341	Tac2	-1.740	0.005479	Down	Gm36899	1.386	0.033199	Up
342	Crabp2	-1.746	0.000000	Down	Nxf7	1.373	0.012874	Up
343	Slc6a13	-1.767	0.000000	Down	Snhg3	1.367	0.008523	Up
344	Gm47173	-1.773	0.001881	Down	Tmem215	1.366	0.032579	Up
345	Aox3	-1.791	0.000077	Down	Tcaf2	1.357	0.024147	Up
346	Gabra6	-1.821	0.046028	Down	Igf2	1.350	0.009367	Up
347	Fmod	-1.831	0.001707	Down	Emb	1.337	0.023994	Up

348	Clrn3	-1.835	0.038018	Down	Ggn	1.333	0.028197	Up
349	Tnfsf13	-1.849	0.002334	Down	Ucp2	1.326	0.038335	Up
350	Gm16023	-1.869	0.010471	Down	Hkdc1	1.325	0.040314	Up
351	5830408C22Rik	-1.892	0.044325	Down	Ntn5	1.322	0.049024	Up
352	Gm8979	-1.892	0.015052	Down	9530018F02Rik	1.318	0.024727	Up
353	Vsx1	-1.897	0.004124	Down	Tbx3os1	1.316	0.017856	Up
354	Gm17120	-1.898	0.010681	Down	Pnlip	1.308	0.005034	Up
355	Cilp2	-1.903	0.000001	Down	Sdc1	1.306	0.013001	Up
356	Pklr	-1.907	0.035055	Down	C1ra	1.303	0.004982	Up
357	Ifi27l2b	-1.913	0.024906	Down	Olfr78	1.290	0.032330	Up
358	Ugt2b34	-1.951	0.010355	Down	Ceacam2	1.289	0.004731	Up
359	Plekhg6	-1.955	0.000683	Down	Sec1	1.288	0.030824	Up
360	Gata6	-1.977	0.041182	Down	Capsl	1.280	0.038890	Up
361	Gm26621	-1.980	0.049406	Down	Gm28455	1.262	0.038508	Up

362	Il1rl1	-1.982	0.016533	Down	Acr	1.259	0.025190	Up
363	Foxd2os	-1.989	0.003447	Down	C730002L08Rik	1.259	0.011850	Up
364	Wdr86	-1.996	0.001238	Down	Rn7s1	1.251	0.009635	Up
365	Islr	-1.997	0.000000	Down	Plvap	1.246	0.000458	Up
366	Lyve1	-2.014	0.000000	Down	Gm13110	1.237	0.011243	Up
367	Mpz	-2.021	0.003526	Down	Cdsn	1.236	0.046176	Up
368	Ogn	-2.057	0.000008	Down	Glp2r	1.231	0.010166	Up
369	Osr1	-2.068	0.000000	Down	Gsto2	1.229	0.036185	Up
370	Folr2	-2.075	0.014899	Down	Dsp	1.227	0.046705	Up
371	Gbp8	-2.088	0.027629	Down	Tedc1	1.225	0.001313	Up
372	Hoxc6	-2.115	0.002010	Down	Adam33	1.219	0.042485	Up
373	Xpnpep2	-2.122	0.035497	Down	Gm10570	1.211	0.049281	Up
374	Apol7a	-2.123	0.010260	Down	Sgms2	1.208	0.001635	Up
375	Six2	-2.149	0.000113	Down	Mybpc1	1.206	0.011812	Up

376	Pcdha11	-2.153	0.000012	Down	Ifitm2	1.205	0.000480	Up
377	Mrpl27-ps	-2.156	0.001305	Down	Pcolce	1.201	0.004225	Up
378	Gm15764	-2.170	0.048678	Down	A730011C13Rik	1.200	0.008214	Up
379	Igkv8-30	-2.172	0.038232	Down	Hbb-bs	1.192	0.020430	Up
380	Mpzl2	-2.177	0.001590	Down	Rn7s2	1.191	0.012001	Up
381	Gm50471	-2.178	0.044686	Down	Gm10654	1.191	0.020345	Up
382	Abcg8	-2.189	0.029820	Down	Gabrr2	1.189	0.006745	Up
383	Soat2	-2.191	0.040578	Down	Ccl9	1.186	0.009146	Up
384	Hcar1	-2.198	0.000000	Down	Krt12	1.186	0.002600	Up
385	Slc13a4	-2.209	0.001490	Down	Spint2	1.186	0.025393	Up
386	Hnf4g	-2.227	0.044794	Down	Nid2	1.186	0.000382	Up
387	Slc26a7	-2.253	0.000000	Down	BC024139	1.186	0.031539	Up
388	Igfbp1	-2.284	0.015912	Down	Slc24a5	1.183	0.000616	Up
389	Gxylt2	-2.303	0.000006	Down	Gm15675	1.176	0.025515	Up

390	Tmem221	-2.312	0.010160	Down	Mr1	1.167	0.000087	Up
391	Fam180a	-2.353	0.000000	Down	Acss3	1.164	0.044164	Up
392	Pagr1a	-2.357	0.000182	Down	Plac9a	1.161	0.049949	Up
393	Ranbp3l	-2.376	0.000000	Down	Tspan11	1.160	0.021093	Up
394	Mogat2	-2.388	0.004652	Down	Hba-a1	1.159	0.037323	Up
395	Npc1l1	-2.415	0.000185	Down	Tmem98	1.147	0.000002	Up
396	Wnt6	-2.442	0.000000	Down	Gm7327	1.146	0.042310	Up
397	Il13ra2	-2.489	0.000232	Down	Gprc5c	1.143	0.016450	Up
398	Igsf23	-2.498	0.047020	Down	Rilp	1.141	0.030075	Up
399	Naaladl1	-2.532	0.000171	Down	Angpt2	1.139	0.009696	Up
400	Tm4sf20	-2.550	0.031991	Down	E230016M11Rik	1.136	0.000211	Up
401	Agtr1b	-2.563	0.010785	Down	Klhl40	1.132	0.006200	Up
402	Alx4	-2.571	0.000000	Down	Dnah14	1.132	0.014096	Up
403	Clca4a	-2.585	0.041336	Down	Gm15265	1.131	0.034641	Up

404	Kcnj13	-2.604	0.000001	Down	Inca1	1.124	0.034118	Up
405	Anpep	-2.611	0.010460	Down	D430040D24Rik	1.123	0.040435	Up
406	Trpv1	-2.639	0.017330	Down	Got2-ps1	1.123	0.000396	Up
407	Gm17147	-2.666	0.047645	Down	Fhad1	1.119	0.014994	Up
408	Gm20721	-2.717	0.006405	Down	Ovol2	1.114	0.011046	Up
409	Gm49416	-2.740	0.045401	Down	Msx1	1.113	0.029868	Up
410	4631405J19Rik	-2.761	0.013257	Down	4833418N02Rik	1.111	0.042461	Up
411	Foxb1	-2.767	0.001770	Down	Ncaph	1.110	0.034592	Up
412	Foxc2	-2.771	0.000000	Down	Oas1c	1.109	0.030488	Up
413	Eps8l3	-2.772	0.013203	Down	Serpina3n	1.107	0.039453	Up
414	Gal3st2	-2.778	0.040573	Down	Lox	1.102	0.047040	Up
415	Cyp3a25	-2.861	0.044960	Down	Klhl14	1.100	0.014745	Up
416	Gm33858	-2.901	0.035592	Down	Pla2g3	1.098	0.007100	Up
417	Cd69	-3.056	0.027753	Down	Gm10132	1.094	0.000001	Up

418	Foxl1	-3.078	0.019603	Down	Cela1	1.092	0.008033	Up
419	Gm42957	-3.082	0.020879	Down	Gm38451	1.090	0.031004	Up
420	Hspb3	-3.113	0.004717	Down	Mdfic	1.089	0.000880	Up
421	Drd3	-3.160	0.011321	Down	4932438H23Rik	1.087	0.025576	Up
422	Abcg5	-3.198	0.005210	Down	Lgals3bp	1.086	0.000177	Up
423	Alox15	-3.217	0.048743	Down	Ido2	1.079	0.011492	Up
424	Ugt1a1	-3.295	0.003954	Down	Wnt9a	1.079	0.014581	Up
425	1700073E17Rik	-3.359	0.037677	Down	Cd6	1.078	0.034047	Up
426	Mgam	-3.517	0.011118	Down	Islr	1.075	0.000085	Up
427	L1td1	-3.530	0.036656	Down	Bmx	1.072	0.019419	Up
428	Btnl5-ps	-3.530	0.011613	Down	Aard	1.071	0.025814	Up
429	Apof	-3.575	0.000203	Down	Fzd4	1.067	0.044112	Up
430	Gm12829	-3.639	0.001026	Down	Fas	1.066	0.009292	Up
431	Slc34a2	-3.672	0.001607	Down	Ak8	1.059	0.023958	Up

432	Ighv1-71	-3.726	0.008084	Down	Tmem220	1.054	0.034361	Up
433	Slc5a1	-3.751	0.012497	Down	Acacb	1.048	0.001463	Up
434	Ighv3-6	-3.757	0.000000	Down	Crhr2	1.045	0.021634	Up
435	Ppp1r14d	-3.793	0.045811	Down	Mst1r	1.043	0.025758	Up
436	H2-T3	-3.807	0.009101	Down	2810049E08Rik	1.040	0.018349	Up
437	Cyp4f40	-3.857	0.027819	Down	Car14	1.036	0.000078	Up
438	Nkx2-1	-4.017	0.000911	Down	Gm26672	1.035	0.046143	Up
439	AC167036	-4.029	0.037072	Down	St6galnac2	1.029	0.002219	Up
440	Zfp784	-4.096	0.014498	Down	Angptl2	1.025	0.002228	Up
441	Gm3127	-4.106	0.016204	Down	Stard6	1.016	0.044180	Up
442	2810433D01Rik	-4.164	0.004807	Down	Gm2004	1.013	0.015139	Up
443	Slc15a1	-4.299	0.011039	Down	Ccdc173	1.008	0.041240	Up
444	Ighv1-62-2	-4.329	0.011048	Down	Itpripl1	1.007	0.007591	Up
445	Sfta3-ps	-4.334	0.002916	Down	Birc3	1.006	0.037468	Up

446	Timd4	-4.360	0.004038	Down	Tgfbi	1.001	0.002043	Up
447	Igkv17-127	-4.470	0.000000	Down	Gm45380	-1.005	0.027626	Down
448	Sis	-4.521	0.017518	Down	Nrp2	-1.008	0.000000	Down
449	Adamts13	-4.585	0.036573	Down	Frmpd2	-1.022	0.027764	Down
450	Fgf2	-4.766	0.000000	Down	AW551984	-1.028	0.007992	Down
451	1600029O15Rik	-5.608	0.016535	Down	Tubd1	-1.029	0.029526	Down
452	Defa33	-5.641	0.042206	Down	Rps13-ps1	-1.043	0.001595	Down
453	Gm42489	-6.193	0.004690	Down	Gm12696	-1.056	0.019831	Down
454	Igkv12-41	-6.873	0.000007	Down	Cdhr4	-1.060	0.040241	Down
455	Alx1	-7.120	0.044281	Down	Npas4	-1.074	0.003915	Down
456	Igkv4-50	-7.221	0.035526	Down	Cartpt	-1.078	0.011540	Down
457	Gm10825	-7.850	0.021279	Down	Fbxw23	-1.099	0.032477	Down
458	Gm15482	-8.138	0.032235	Down	Lgals12	-1.102	0.041240	Down
459	Gm12784	-8.271	0.040063	Down	Fgf3	-1.105	0.038631	Down

460	A830019L24Rik	-8.357	0.042012	Down	Ptp4a1	-1.110	0.002597	Down
461	Hal	-8.408	0.010258	Down	1700020D05Rik	-1.118	0.049715	Down
462	Zbbx	-8.450	0.017989	Down	C3	-1.139	0.036712	Down
463	Tcp10c	-8.462	0.036469	Down	Notum	-1.168	0.007936	Down
464	Gm48405	-8.556	0.048264	Down	P2ry1	-1.180	0.000179	Down
465	Ldlrad2	-8.567	0.026480	Down	3632454L22Rik	-1.223	0.040456	Down
466	Gm42894	-8.639	0.031436	Down	Mirt1	-1.224	0.032619	Down
467	Gm28177	-8.814	0.014544	Down	Ccdc110	-1.233	0.049445	Down
468	Kcng3	-8.856	0.005580	Down	Gm6685	-1.235	0.008831	Down
469	Gm45041	-8.881	0.005069	Down	2310034G01Rik	-1.241	0.025257	Down
470	Samd13	-8.917	0.036001	Down	Crh	-1.270	0.000535	Down
471	Gbp2b	-8.956	0.009356	Down	Odf3b	-1.275	0.005208	Down
472	Gm34388	-8.973	0.045052	Down	Ndst4	-1.309	0.006058	Down
473	Gm39214	-9.231	0.033900	Down	Zfp618	-1.318	0.000002	Down

474	Gm10933	-9.243	0.019843	Down	Aspm	-1.337	0.020971	Down
475	Kng1	-9.330	0.008293	Down	Peak1	-1.373	0.006726	Down
476	Cmtm1	-9.407	0.004207	Down	Sap30bpos	-1.382	0.044472	Down
477	Gm34376	-9.410	0.021903	Down	Trav9d-4	-1.382	0.031571	Down
478	Chst13	-9.621	0.028855	Down	Ccdc121	-1.401	0.025442	Down
479	Slc2a7	-9.788	0.037284	Down	Mmp23	-1.427	0.012613	Down
480	Osr2	-9.793	0.000224	Down	Gm5176	-1.433	0.046788	Down
481	Slc5a4a	-9.794	0.010654	Down	Zfp968-ps	-1.441	0.014612	Down
482	C630004L07Rik	-9.799	0.016932	Down	AL731706	-1.461	0.000483	Down
483	Cyp21a1	-9.799	0.013190	Down	Gm6829	-1.473	0.044395	Down
484	Gm28703	-9.896	0.019526	Down	Prlhr	-1.510	0.014455	Down
485	Bdkrb1	-9.943	0.009316	Down	Gm43375	-1.521	0.044864	Down
486	Gm15684	-9.986	0.009213	Down	Chst5	-1.533	0.003022	Down
487	Phf11a	-10.052	0.006380	Down	Rps13-ps2	-1.564	0.000002	Down

488	B930018H19Rik	-10.147	0.000290	Down	Gm49027	-1.569	0.000562	Down
489	Gm11532	-10.174	0.048959	Down	Glp1r	-1.575	0.016170	Down
490	Selenok-ps1	-10.335	0.023388	Down	Zfp474	-1.596	0.035463	Down
491	Ugt2a3	-10.398	0.000570	Down	Cckar	-1.638	0.028212	Down
492	Gm32341	-10.555	0.000434	Down	Pla2g4b	-1.645	0.000560	Down
493	H60b	-10.632	0.007907	Down	Dsc3	-1.655	0.003397	Down
494	Kcne3	-10.635	0.000357	Down	Gm7666	-1.667	0.000002	Down
495	Gm39929	-10.659	0.045989	Down	Gpr101	-1.706	0.000000	Down
496	Gm20458	-10.748	0.015675	Down	Dkk2	-1.723	0.041915	Down
497	Gm16073	-10.808	0.030872	Down	Calr4	-1.731	0.030584	Down
498	Cfhr2	-10.833	0.000003	Down	Npsr1	-1.826	0.003356	Down
499	Pla2g2a	-10.881	0.034714	Down	Gm46603	-1.835	0.013872	Down
500	Gm39653	-10.948	0.005068	Down	Gm4895	-1.836	0.007576	Down
501	Gm11379	-11.117	0.040033	Down	Gm5540	-1.848	0.030494	Down

502	Gm27027	-11.212	0.010411	Down	Dlk1	-1.862	0.000000	Down
503	Apcs	-11.223	0.016610	Down	Gm43333	-1.862	0.034233	Down
504	Lypd2	-11.244	0.003496	Down	Nme9	-1.884	0.035078	Down
505	Gm12932	-11.281	0.004936	Down	Rnf138rt1	-1.990	0.022237	Down
506	Gm47655	-11.353	0.029000	Down	Dgkk	-1.993	0.000167	Down
507	Gm17546	-11.380	0.001374	Down	Glcci1	-2.007	0.000000	Down
508	Gzma	-11.390	0.001963	Down	Gm45353	-2.021	0.041543	Down
509	Serpina1e	-11.718	0.000008	Down	4930579K19Rik	-2.127	0.009882	Down
510	Ighv5-6	-11.820	0.033784	Down	Daw1	-2.146	0.020447	Down
511	Hbb-y	-12.641	0.000045	Down	Mrpl27-ps	-2.212	0.012472	Down
512	Gm20431	-13.598	0.000000	Down	Gm8010	-2.246	0.010966	Down
513					Pagr1a	-2.298	0.002078	Down
514					Gm609	-2.303	0.033465	Down
515					Kif14	-2.449	0.017221	Down

516	Ccdc105	-2.541	0.042736	Down
517	Gm50270	-2.681	0.024528	Down
518	Gm45890	-2.690	0.029928	Down
519	Gm15455	-2.694	0.045316	Down
520	Dusp27	-2.708	0.006783	Down
521	Alb	-2.909	0.000053	Down
522	Foxc2	-2.921	0.022713	Down
523	Slc2a2	-3.428	0.045046	Down
524	Gm3654	-3.490	0.030565	Down
525	Gm14205	-3.648	0.030628	Down
526	E230020D15Rik	-3.852	0.025205	Down
527	H3c14	-3.923	0.002419	Down
528	Serpina1b	-4.085	0.045758	Down
529	Skida1	-4.088	0.000000	Down

530	Gm43573	-4.090	0.007249	Down
531	Gm50105	-4.140	0.011288	Down
532	Ush2a	-5.695	0.043452	Down
533	Gm29154	-6.811	0.041001	Down
534	Gm43327	-7.716	0.046023	Down
535	Hpse2	-7.984	0.011878	Down
536	Vmn1r88	-8.106	0.012195	Down
537	Bhlha15	-8.210	0.025244	Down
538	Lncenc1	-8.282	0.021740	Down
539	Gm26696	-8.340	0.035082	Down
540	Nphs2	-8.449	0.048978	Down
541	6430553K19Rik	-8.759	0.024054	Down
542	Kng1	-8.785	0.021723	Down
543	Spata20	-8.864	0.029247	Down

544	Gm30504	-8.872	0.009102	Down
545	Gm28187	-8.922	0.025244	Down
546	Gm50269	-9.032	0.012271	Down
547	Gm48843	-9.113	0.028549	Down
548	Gm44553	-9.211	0.012532	Down
549	Gm20539	-9.266	0.033713	Down
550	Gm35154	-9.291	0.042096	Down
551	Asb9	-9.374	0.049537	Down
552	Gm41556	-9.398	0.010715	Down
553	Gm5779	-9.439	0.030242	Down
554	Gm29480	-9.536	0.010312	Down
555	Gm50100	-9.572	0.034373	Down
556	3110062G12Rik	-9.578	0.021609	Down
557	Ube2u	-9.623	0.009335	Down

558	Gm11855	-9.681	0.003593	Down
559	Gm17409	-10.123	0.017032	Down
560	Gm35611	-10.220	0.028675	Down
561	Gm13610	-10.420	0.006545	Down
562	Cfap221	-10.439	0.000004	Down
563	Lyz1	-10.512	0.008262	Down
564	Gm15367	-10.574	0.015382	Down
565	Gm20458	-10.720	0.016232	Down
566	Rap1a-ps2	-10.766	0.025456	Down
567	Gm10702	-10.801	0.016871	Down
568	Gm32688	-10.820	0.016944	Down
569	Orm1	-11.031	0.039594	Down
570	Rps18-ps4	-11.103	0.014328	Down
571	Calcr	-11.253	0.000000	Down

572	Retnla	-11.332	0.016433	Down
573	Fga	-11.452	0.037461	Down
574	Hbb-y	-11.667	0.001590	Down
575	Gm36495	-11.700	0.000480	Down
576	Ccl8	-12.033	0.000985	Down
577	Igkv4-74	-12.184	0.013464	Down

Abbreviation: FC, fold change.

Table S4. Differentially expressed proteins in the proteomes of the SN and STR of A53T+IDE/A53T+Vector mice.

NO.	Gene symbol	FC	p-value	SN Type	Gene symbol	Fold chang	p-value	STR Type
1	Ppp1r16a	34.689	0.014306*	Up	Bcl7a	175.186	0.018978	Up
2	Jhy	34.664	0.036377	Up	Fkbpl	104.114	0.030710	Up
3	Thoc5	28.901	0.027914	Up	Dohh	103.424	0.038290	Up
4	Bbs9	28.352	0.014306*	Up	Fchsd2	88.670	0.014306*	Up
5	Prmt9	27.561	0.048861	Up	Fmo2	67.157	0.028468	Up
6	Fbxw5	19.490	0.030025	Up	Agt	35.023	0.001439	Up
7	Nhsl2	18.767	0.002189	Up	Wdr43	31.678	0.046403	Up
8	Plxna2	9.435	0.004025	Up	Edem3	29.131	0.034111	Up
9	Inpp5b	6.825	0.002875	Up	Tax1bp3	25.145	0.014306*	Up
10	Zftraf1	6.063	0.027468	Up	Dnaaf1	8.220	0.001727	Up
11	Qsox1	5.920	0.007780	Up	Taok3	7.615	0.017731	Up

12	Unk	5.581	0.007599	Up	Clgn	7.595	0.039593	Up
13	Exosc4	4.642	0.011264	Up	Exosc9	7.567	0.014457	Up
14	Ptgis	4.539	0.048250	Up	Hecw1	7.143	0.038852	Up
15	Mrto4	4.211	0.032124	Up	Ankrd49	6.271	0.026237	Up
16	Lingo3	4.210	0.028728	Up	Gbp2	6.260	0.007547	Up
17	Omp	4.159	0.031390	Up	Caskin2	6.137	0.004877	Up
18	Lrrc8b	3.962	0.008979	Up	S100a11	6.089	0.019744	Up
19	Slc22a8	3.956	0.036637	Up	Plxdc1	5.593	0.032707	Up
20	Exosc7	3.928	0.043456	Up	Unc45a	5.191	0.036514	Up
21	Morf4l1	3.890	0.023547	Up	Rimbp3	4.936	0.039477	Up
22	Spry4	3.837	0.045967	Up	Pum2	4.810	0.008252	Up
23	Csnk1g2	3.706	0.038560	Up	Pak4	4.132	0.045350	Up
24	Stom	3.182	0.047891	Up	Sowahc	3.766	0.038758	Up
25	Ca3	3.060	0.024023	Up	Mkrn2	3.761	0.030962	Up

26	Tpcn1	3.043	0.044601	Up	Eif5a2	3.187	0.016716	Up
27	Alg13	2.941	0.017577	Up	Ctsl	2.972	0.033480	Up
28	Kank2	2.695	0.032888	Up	Coq10b	2.658	0.048138	Up
29	Bbox1	2.193	0.004711	Up	Fars2	2.516	0.039400	Up
30	Myl9	2.062	0.030483	Up	Pabpn1	2.098	0.005276	Up
31	Mrpl52	2.038	0.007907	Up	Bace1	2.035	0.008713	Up
32	Cnot10	1.866	0.017624	Up	Ppm1k	1.879	0.029342	Up
33	Hdac1	1.829	0.019342	Up	Gcat	1.872	0.044635	Up
34	Sdhaf4	1.751	0.031810	Up	Cdc26	1.857	0.010923	Up
35	Smarca2	1.707	0.011205	Up	Cnot11	1.844	0.045145	Up
36	Poldip3	1.704	0.009714	Up	Slc35g2	1.755	0.020967	Up
37	Map3k7	1.674	0.033759	Up	Cnnm3	1.742	0.018132	Up
38	Cp	1.655	0.029619	Up	Ppig	1.735	0.012448	Up
39	Slc25a44	1.646	0.035040	Up	Pxn	1.711	0.007528	Up

40	Slc7a10	1.625	0.015540	Up	Ttyh2	1.657	0.015567	Up
41	Ccdc88a	1.606	0.003775	Up	Mob1b	1.626	0.047702	Up
42	Tmem41b	1.604	0.000763	Up	Ergic3	1.612	0.022768	Up
43	Sdk2	1.595	0.038340	Up	Rab28	1.578	0.009092	Up
44	Slc25a21	1.565	0.015545	Up	Mmgt1	1.558	0.028674	Up
45	Ldb3	1.558	0.049557	Up	Haghl	1.555	0.021791	Up
46	Wdr45	1.550	0.002463	Up	Elp4	1.519	0.008381	Up
47	Ptgds	1.543	0.046940	Up	Gar1	1.514	0.010565	Up
48	Fbxo45	1.542	0.041846	Up	Vkorc1l1	1.500	0.012292	Up
49	Cnst	1.531	0.030855	Up	Crip1	1.491	0.040471	Up
50	Plaat3	1.517	0.023370	Up	Sdhaf1	1.456	0.031646	Up
51	Strip2	1.516	0.017500	Up	Mpi	1.450	0.001390	Up
52	Kdsr	1.494	0.033349	Up	Rps28	1.445	0.026966	Up
53	Slc18a3	1.492	0.031023	Up	Cdh6	1.431	0.039838	Up

54	Tdrp	1.490	0.046328	Up	Celf3	1.412	0.018096	Up
55	Tmem201	1.485	0.023725	Up	Ccdc90b	1.394	0.033080	Up
56	Mavs	1.469	0.013201	Up	Pcbd1	1.385	0.033268	Up
57	Dennd1a	1.467	0.002548	Up	Sqstm1	1.385	0.010522	Up
58	Nars2	1.462	0.022740	Up	Rragc	1.364	0.019525	Up
59	Bnip3l	1.453	0.045232	Up	Commd5	1.360	0.004958	Up
60	Slc25a14	1.435	0.021242	Up	Tspyl4	1.357	0.040276	Up
61	Acsl5	1.429	0.032872	Up	Dtx3	1.349	0.019783	Up
62	Pnn	1.412	0.011231	Up	Clptm1l	1.347	0.019427	Up
63	Rab28	1.412	0.021291	Up	Rngtt	1.340	0.000116	Up
64	Spred1	1.374	0.009440	Up	Rars2	1.340	0.034462	Up
65	Ppip5k1	1.373	0.028044	Up	Clpb	1.340	0.017015	Up
66	Spcs1	1.360	0.021655	Up	Ly6h	1.339	0.040823	Up
67	Ctsa	1.359	0.043481	Up	Ublcp1	1.335	0.011779	Up

68	Ndufaf7	1.357	0.016801	Up	Ddx21	1.318	0.000174	Up
69	Capg	1.343	0.031220	Up	Mogs	1.317	0.033810	Up
70	Dkc1	1.340	0.049680	Up	Srsf10	1.316	0.009276	Up
71	Tinagl1	1.330	0.018395	Up	Anapc4	1.314	0.000609	Up
72	Rab36	1.327	0.002296	Up	Rab23	1.305	0.003756	Up
73	Itch	1.325	0.014723	Up	Arl1	1.301	0.037073	Up
74	Gjb1	1.323	0.010185	Up	Mfsd6	1.298	0.046015	Up
75	Mettl13	1.317	0.009088	Up	Rps6	1.287	0.006326	Up
76	Prune2	1.312	0.046146	Up	Rmc1	1.280	0.037788	Up
77	Tm9sf4	1.312	0.011448	Up	Mcee	1.279	0.028096	Up
78	Poldip2	1.311	0.046515	Up	Enpep	1.269	0.029806	Up
79	Ppp1r8	1.311	0.039860	Up	Abl2	1.267	0.020961	Up
80	Tesc	1.306	0.019969	Up	Grm7	1.260	0.009737	Up
81	Tdrd7	1.300	0.032360	Up	Rer1	1.253	0.016195	Up

82	Ubac2	1.298	0.035253	Up	Erlin1	1.253	0.000775	Up
83	Ylpm1	1.279	0.043246	Up	Galk1	1.245	0.014523	Up
84	Adhfe1	1.275	0.022422	Up	Itm2c	1.243	0.017087	Up
85	Cd2ap	1.275	0.034207	Up	Mrpl39	1.241	0.045166	Up
86	Grin2b	1.275	0.048311	Up	Tmem35a	1.235	0.015628	Up
87	Rbm14	1.274	0.035442	Up	Cnih2	1.228	0.001324	Up
88	Fam234b	1.263	0.011245	Up	Bltp2	1.223	0.007267	Up
89	Slmap	1.256	0.026312	Up	Cluh	1.223	0.022811	Up
90	Ufl1	1.247	0.006438	Up	Pycr3	1.220	0.014311	Up
91	Naga	1.244	0.014998	Up	Glrx3	1.219	0.014954	Up
92	Tpbg	1.239	0.047273	Up	Neto2	1.214	0.049941	Up
93	Fam120b	1.233	0.011039	Up	Sms	1.212	0.000183	Up
94	Smarcc2	1.232	0.045577	Up	Mrpl9	1.205	0.016679	Up
95	Abcb1a	1.232	0.039824	Up	Map9	1.205	0.029826	Up

96	Txndc5	1.230	0.044005	Up	Ggps1	0.827	0.042716	Down
97	Map6d1	1.228	0.018915	Up	Ccdc22	0.826	0.045960	Down
98	Faah	1.228	0.015727	Up	Map6d1	0.819	0.014195	Down
99	Rragc	1.227	0.026573	Up	Chd4	0.815	0.024799	Down
100	Galk1	1.227	0.023902	Up	Zbtb8os	0.814	0.027976	Down
101	Col4a2	1.223	0.003756	Up	Ubqln4	0.812	0.043136	Down
102	Ntrk3	1.217	0.021345	Up	Dnajb1	0.812	0.006550	Down
103	Atl3	1.215	0.026724	Up	Tmem109	0.808	0.010894	Down
104	Stt3a	1.212	0.021642	Up	Rufy2	0.803	0.019893	Down
105	Scfd2	1.209	0.043187	Up	Parva	0.801	0.004713	Down
106	Plekha6	1.202	0.013647	Up	Dock7	0.799	0.032970	Down
107	Opa3	0.826	0.027467	Down	Lrrc4b	0.798	0.035437	Down
108	Mios	0.825	0.043496	Down	Stx4	0.798	0.038342	Down
109	Vmp1	0.825	0.040941	Down	Eif2b5	0.797	0.023968	Down

110	Nova2	0.825	0.000720	Down	Flywch1	0.794	0.024046	Down
111	Bnip1	0.822	0.020299	Down	Amfr	0.789	0.033138	Down
112	Grm4	0.821	0.025438	Down	Chmp1b2	0.788	0.003961	Down
113	Poglut1	0.820	0.026899	Down	Cd2ap	0.787	0.046497	Down
114	Kank4	0.818	0.008316	Down	Ppp1r14a	0.786	0.044169	Down
115	Cspg5	0.817	0.036983	Down	Svip	0.782	0.019312	Down
116	Septin9	0.806	0.004721	Down	Smchd1	0.776	0.001413	Down
117	Psmg1	0.805	0.021107	Down	Wars2	0.776	0.046188	Down
118	Acot13	0.802	0.011981	Down	Kctd9	0.772	0.029249	Down
119	Araf	0.790	0.003161	Down	Camk2n1	0.766	0.044644	Down
120	Mrps5	0.788	0.046626	Down	Myo1c	0.763	0.019575	Down
121	Calm1	0.787	0.047354	Down	Agbl4	0.762	0.019098	Down
122	Fxr2	0.787	0.016401	Down	Top1	0.761	0.015329	Down
123	Snca	0.775	0.034768	Down	Cryab	0.761	0.031411	Down

124	Gnai3	0.767	0.029966	Down	Mapk8	0.760	0.019326	Down
125	Cert1	0.764	0.043422	Down	Otud7b	0.759	0.009263	Down
126	Atp5f1e	0.758	0.003690	Down	Rrm2b	0.755	0.003393	Down
127	Dhrs7b	0.753	0.024124	Down	Plscr3	0.755	0.049971	Down
128	Eras	0.744	0.017822	Down	Kit	0.749	0.032021	Down
129	Cox7c	0.732	0.027118	Down	Lrrtm3	0.748	0.006622	Down
130	Pex5	0.731	0.004890	Down	Yme1l1	0.743	0.025740	Down
131	Lsm3	0.722	0.023331	Down	Csad	0.742	0.017026	Down
132	Enho	0.720	0.040351	Down	Ebp	0.742	0.005058	Down
133	Kbtbd2	0.719	0.011857	Down	Selenof	0.741	0.041743	Down
134	Cplx2	0.708	0.037292	Down	Sirt3	0.738	0.023424	Down
135	Ptk7	0.701	0.012854	Down	Mindy2	0.729	0.024810	Down
136	Spock1	0.701	0.015365	Down	Dhx29	0.729	0.020755	Down
137	Vps9d1	0.697	0.045168	Down	Eif2b1	0.726	0.046619	Down

138	C4b	0.693	0.044312	Down	Cd99l2	0.721	0.049686	Down
139	Tmeff1	0.673	0.030365	Down	Dennd6a	0.708	0.033673	Down
140	Insr	0.653	0.015425	Down	Las1l	0.707	0.006757	Down
141	Bace1	0.643	0.005067	Down	Adam9	0.706	0.021195	Down
142	N4bp1	0.642	0.006407	Down	Fbxl20	0.703	0.018532	Down
143	Eif4e2	0.641	0.037600	Down	Scn8a	0.699	0.037397	Down
144	Armh3	0.638	0.038476	Down	Snap23	0.699	0.010583	Down
145	Arhgef17	0.632	0.027967	Down	Gosr1	0.696	0.024075	Down
146	Dop1a	0.606	0.030626	Down	Guf1	0.693	0.034903	Down
147	Shisa9	0.601	0.016187	Down	Aif1l	0.682	0.024380	Down
148	Snx24	0.601	0.002826	Down	Golga2	0.681	0.022122	Down
149	Rnf141	0.596	0.008017	Down	Nkain4	0.679	0.007169	Down
150	Tmem230	0.584	0.001350	Down	Mrpl30	0.670	0.008293	Down
151	Cpped1	0.584	0.029704	Down	Adap2	0.662	0.011692	Down

152	Ccdc50	0.571	0.049765	Down	Trappc2	0.659	0.031101	Down
153	Rhof	0.562	0.041190	Down	Commd7	0.651	0.047159	Down
154	Gnpat	0.466	0.040796	Down	Sec13	0.644	0.006914	Down
155	Lhfpl3	0.371	0.045045	Down	Gng10	0.643	0.000850	Down
156	Ptprm	0.370	0.039661	Down	Rgs3	0.642	0.035463	Down
157	Tubgcp4	0.370	0.042086	Down	Pip4p1	0.638	0.025360	Down
158	Dnajc12	0.352	0.040943	Down	Hmgb2	0.633	0.039546	Down
159	Kifc2	0.275	0.047874	Down	Wdr18	0.632	0.032673	Down
160	Ablim3	0.179	0.023346	Down	Elfn1	0.625	0.044959	Down
161	Gnpnat1	0.173	0.022066	Down	Glmn	0.614	0.007424	Down
162	Lmtk2	0.161	0.018706	Down	Hmgn5	0.602	0.036944	Down
163	Uimc1	0.108	0.003005	Down	Wdr20	0.585	0.039274	Down
164	Clcn7	0.065	0.004465	Down	Mrps17	0.581	0.042274	Down
165	Otub2	0.051	0.014306*	Down	Copz1	0.564	0.017794	Down

166	Ssbp3	0.048	0.014306*	Down	Rundc3b	0.543	0.039688	Down
167	Hdac8	0.042	0.049902	Down	Ca10	0.539	0.008248	Down
168	Metap1d	0.016	0.014306*	Down	Mpp7	0.522	0.038111	Down
169	Has1	0.001	0.002556	Down	Rpf2	0.513	0.012305	Down
170					Sidt1	0.510	0.017520	Down
171					Septin10	0.477	0.016506	Down
172					Derpc	0.474	0.048232	Down
173					S100a1	0.468	0.010111	Down
174					Adck5	0.462	0.011850	Down
175					Ube2d1	0.451	0.003614	Down
176					Hdac3	0.416	0.031956	Down
177					Sat2	0.365	0.038035	Down
178					Krt1	0.334	0.042300	Down
179					Pecam1	0.330	0.035552	Down

180	Asphd1	0.330	0.018458	Down
181	Nf2	0.275	0.015015	Down
182	Ciao3	0.254	0.009147	Down
183	Camkmt	0.249	0.004565	Down
184	Ppp3cc	0.246	0.008215	Down
185	Phospho2	0.241	0.037014	Down
186	Egr1	0.229	0.024798	Down
187	Elac1	0.213	0.002475	Down
188	Flnc	0.210	0.018743	Down
189	Hexd	0.192	0.014868	Down
190	Cdk13	0.164	0.046751	Down
191	Bap18	0.156	0.040170	Down
192	Nfyc	0.155	0.012363	Down
193	Rab3b	0.149	0.022742	Down

194		Sh3rf2	0.115	0.014974	Down
195		Igdcc4	0.023	0.011661	Down
196		Rab34	0.018	0.030736	Down
197		Inppl1	0.016	0.014306*	Down
198		Sptlc2	0.011	0.006762	Down
199		Tspan31	0.011	0.014306*	Down
200		Rai1	0.009	0.049489	Down
201		Rps29	0.001	0.019127	Down

* Differentially expression proteins were displayed with a Chi-square test p-value (p-value-chitest) < 0.05.

Abbreviation: FC, fold change.
