# Supplementary material for: Insulin-degrading enzyme confers neuroprotection in Parkinson’s disease by inhibiting the Hippo signaling pathway
Source: Cell Death Dis. 2025 Oct 24;16(1):758. doi: 10.1038/s41419-025-08055-4 (PMC12552454; doi:10.1038/s41419-025-08055-4)
Supplement: Supplementary file 2 — Original data [file 41419_2025_8055_MOESM2_ESM.pdf]

Original western blots

Insulin-Degrading Enzyme Confers Neuroprotection in Parkinson's Disease by Inhibiting the Hippo Signaling Pathway

Huimin Zheng [1, 2, 3, 4 †], MD; Yu Guo [2, 5†], MD; Shuyu Zhang [1†], MD; Yun Su [1, 2, 3, 4], MD; Xin Cui [1, 2, 3, 4], MD; Zhengwei Hu [1, 2, 3, 4], MD, PhD; Xiaoyan Hao [1, 2, 3, 4], MD; Mengjie Li [1, 2, 3, 4], MD; Changhe Shi [1, 2, 3, 4], MD, PhD; Yuming Xu [1, 2, 3, 4*], MD, PhD; Chengyuan Mao [1, 2, 3, 4*], MD, PhD

[†]: These authors have contributed equally to this work and share first authorship

***Correspondence:**

Chengyuan Mao: maochengyuan2015@126.com

Yuming Xu: xuyuming@zzu.edu.cn

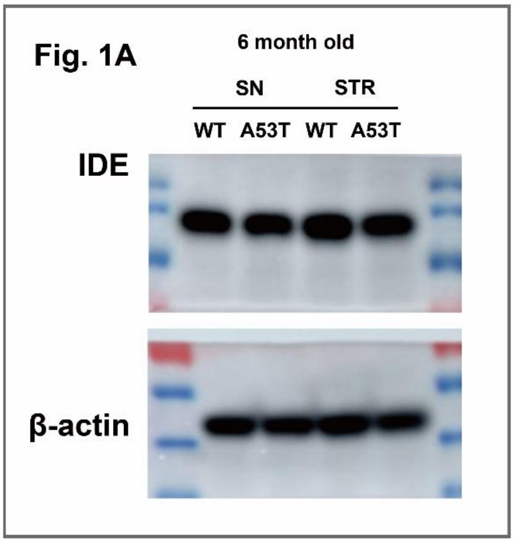
Fig. 1A
6 month old
SN STR
WT A53T WT A53T
IDE
β-actin

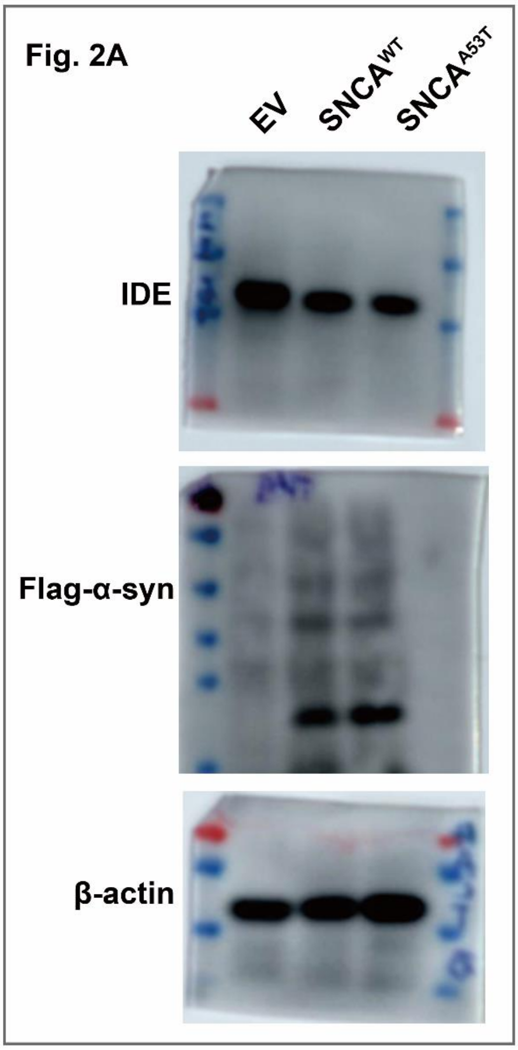
Fig. 2A
EV
SNCA^WT
SNCA^A53T
IDE
Flag-α-syn
β-actin

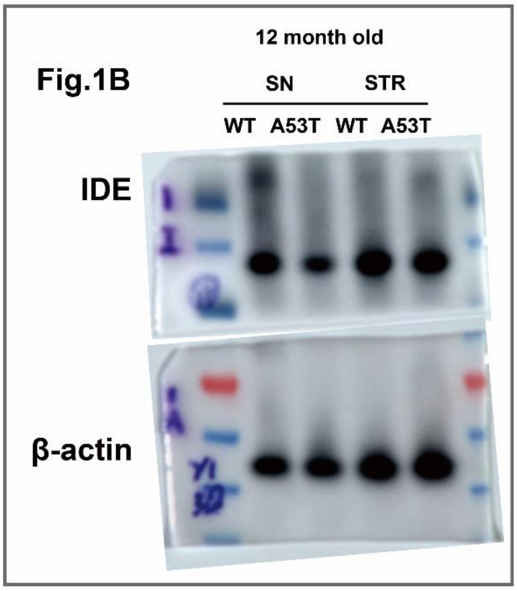
Fig.1B
12 month old
SN STR
WT A53T WT A53T
IDE
β-actin

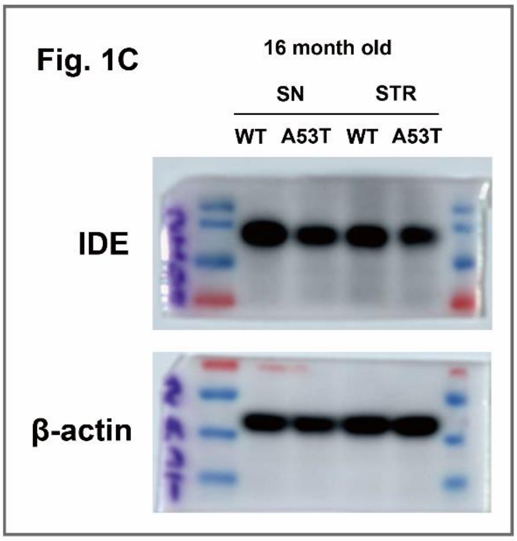
Fig. 1C
16 month old
SN STR
WT A53T WT A53T
IDE
β-actin

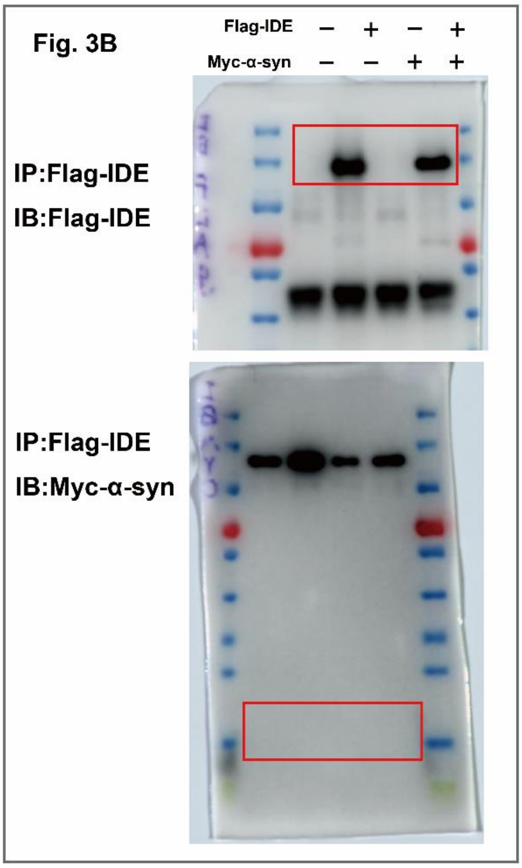
Fig. 3B
Flag-IDE – + – +
Myc-α-syn – – + +
IP:Flag-IDE
IB:Flag-IDE
IP:Flag-IDE
IB:Myc-α-syn

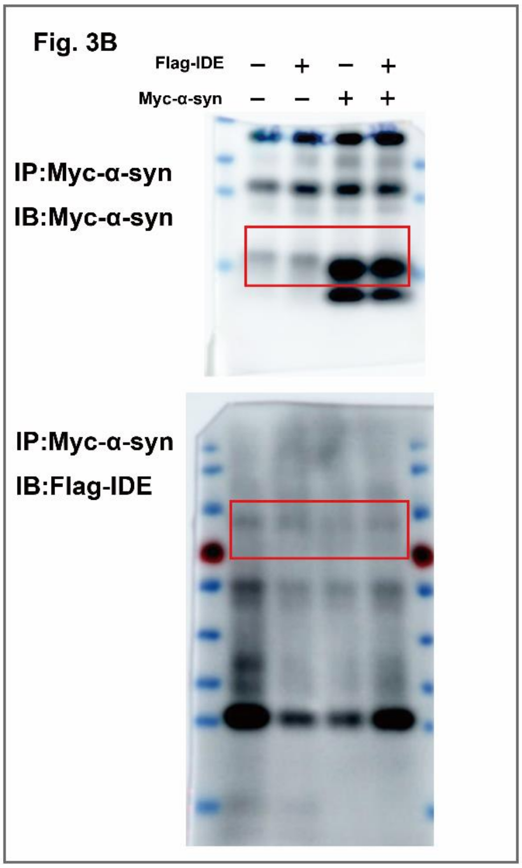
Fig. 3B
Flag-IDE – + – +
Myc-α-syn – – + +
IP:Myc-α-syn
IB:Myc-α-syn
IP:Myc-α-syn
IB:Flag-IDE

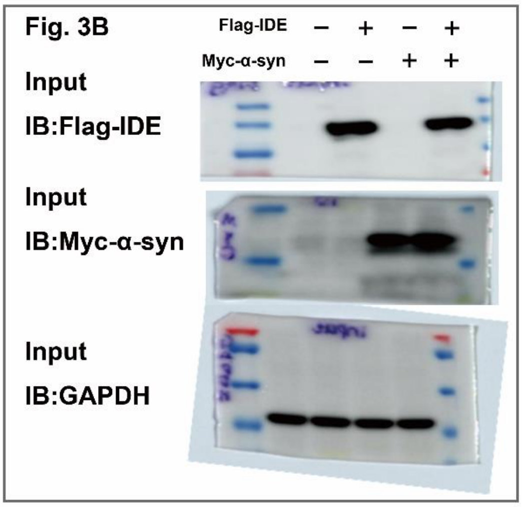
Fig. 3B
Flag-IDE – + – +
Myc-α-syn – – + +
Input
IB:Flag-IDE
Input
IB:Myc-α-syn
Input
IB:GAPDH

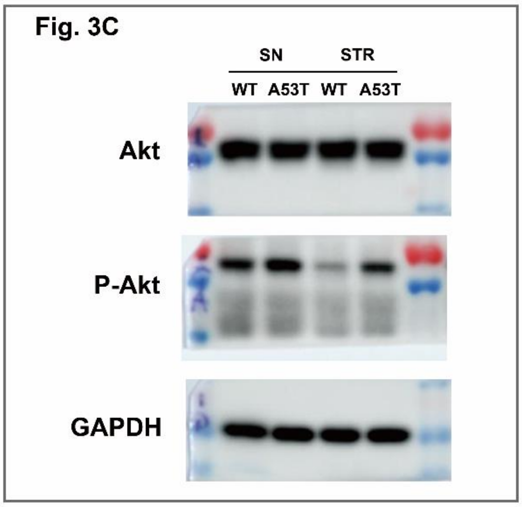
Fig. 3C
SN
STR
WT A53T WT A53T
Akt
P-Akt
GAPDH

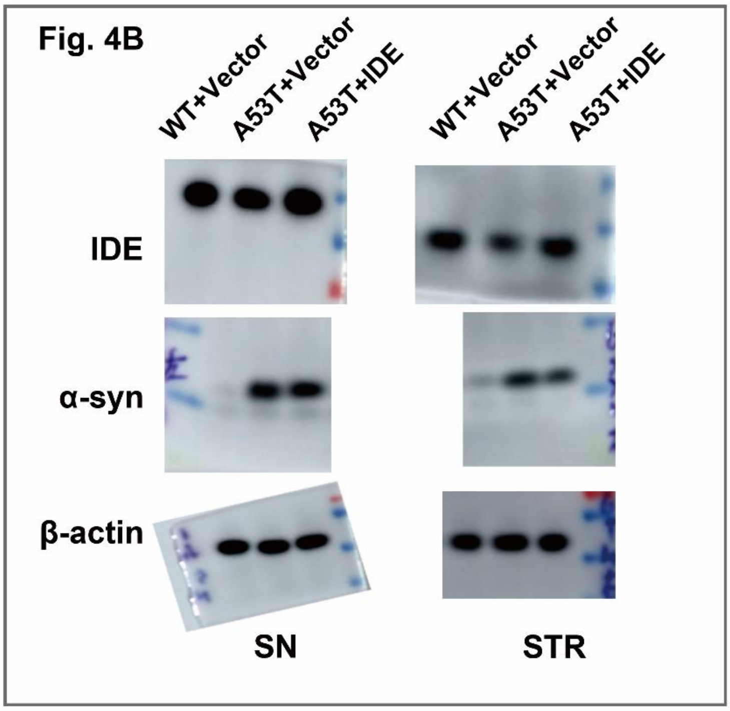
Fig. 4B
WT+Vector
A53T+Vector
A53T+IDE
WT+Vector
A53T+Vector
A53T+IDE
IDE
α-syn
β-actin
SN
STR

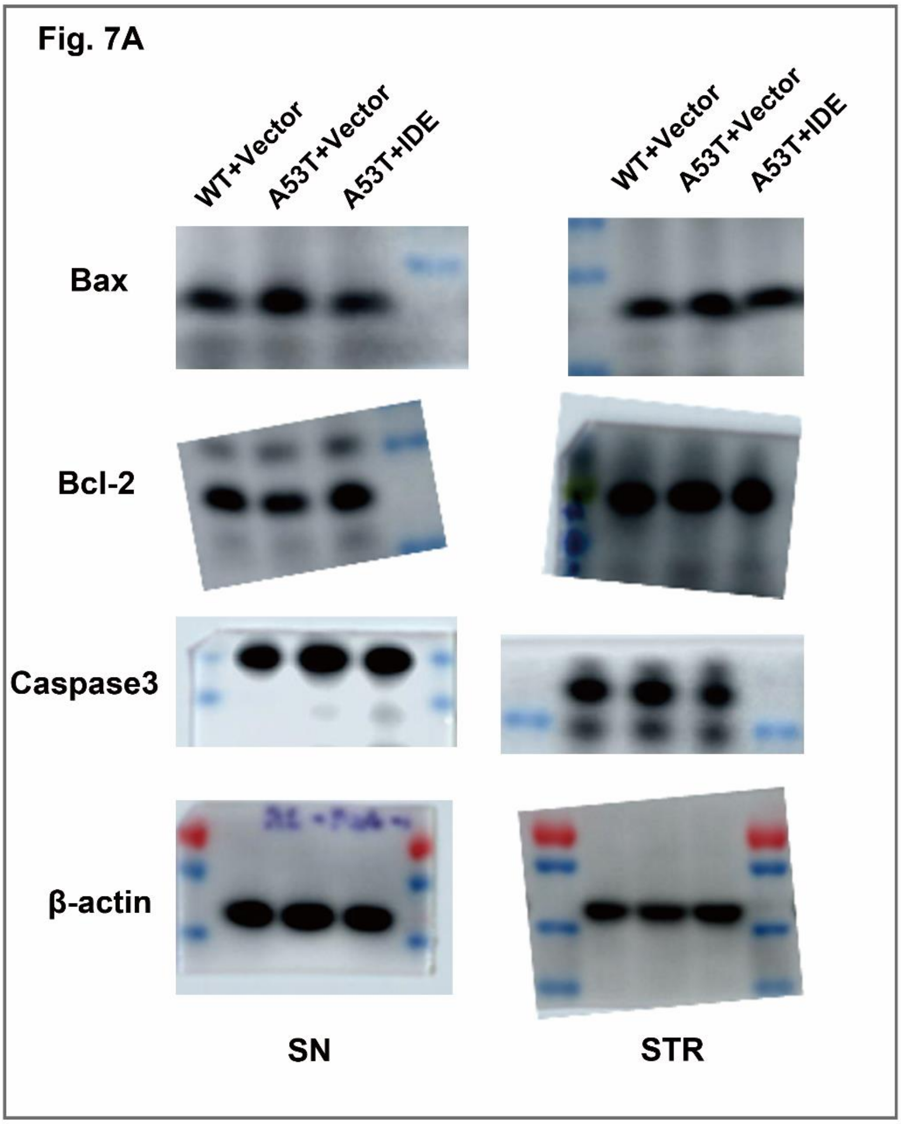
Fig. 7A
WT+Vector
A53T+Vector
A53T+IDE
WT+Vector
A53T+Vector
A53T+IDE
Bax
Bcl-2
Caspase3
β-actin
SN
STR

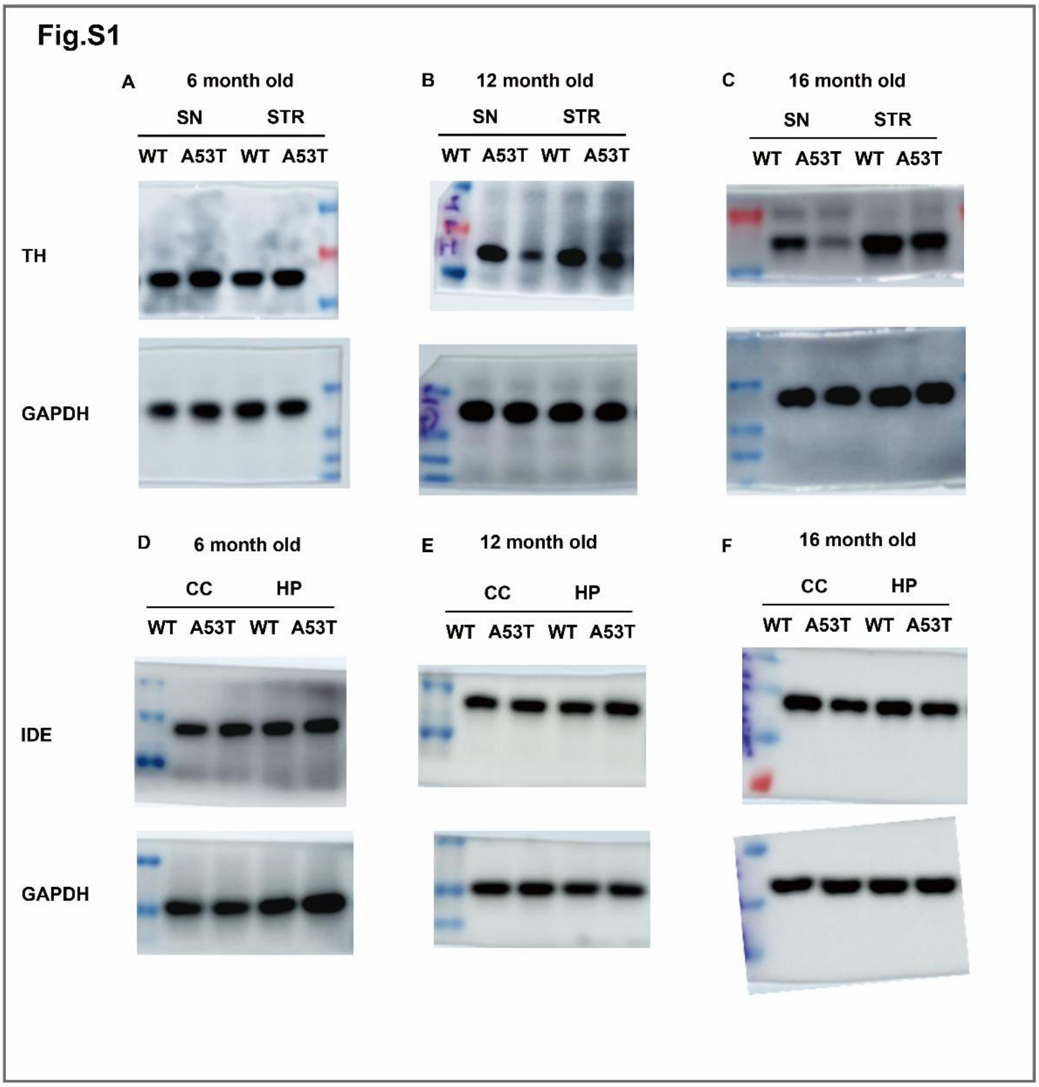

Fig.S1
A
6 month old
SN
STR
WT
A53T
WT
A53T
B
12 month old
SN
STR
WT
A53T
WT
A53T
C
16 month old
SN
STR
WT
A53T
WT
A53T
TH
GAPDH
D
6 month old
CC
HP
WT
A53T
WT
A53T
E
12 month old
CC
HP
WT
A53T
WT
A53T
F
16 month old
CC
HP
WT
A53T
WT
A53T
IDE
GAPDH

Fig. S3A

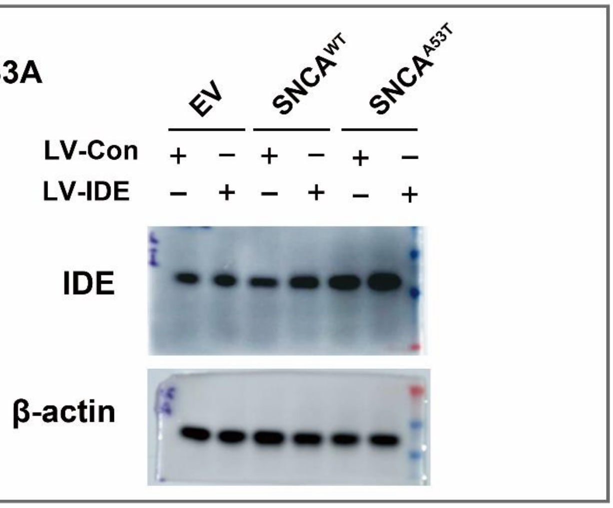

Fig. S3G

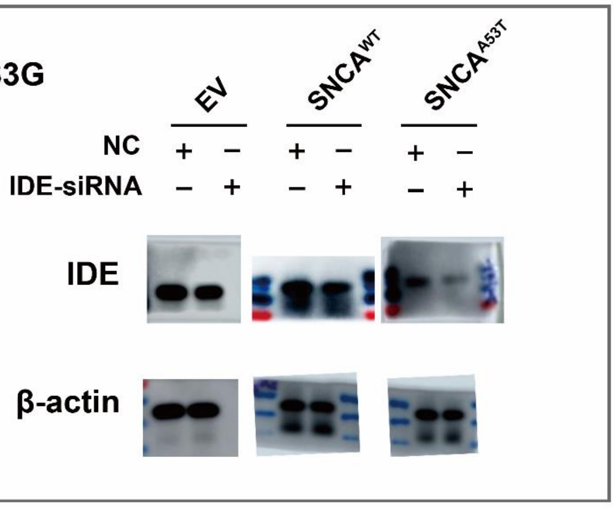

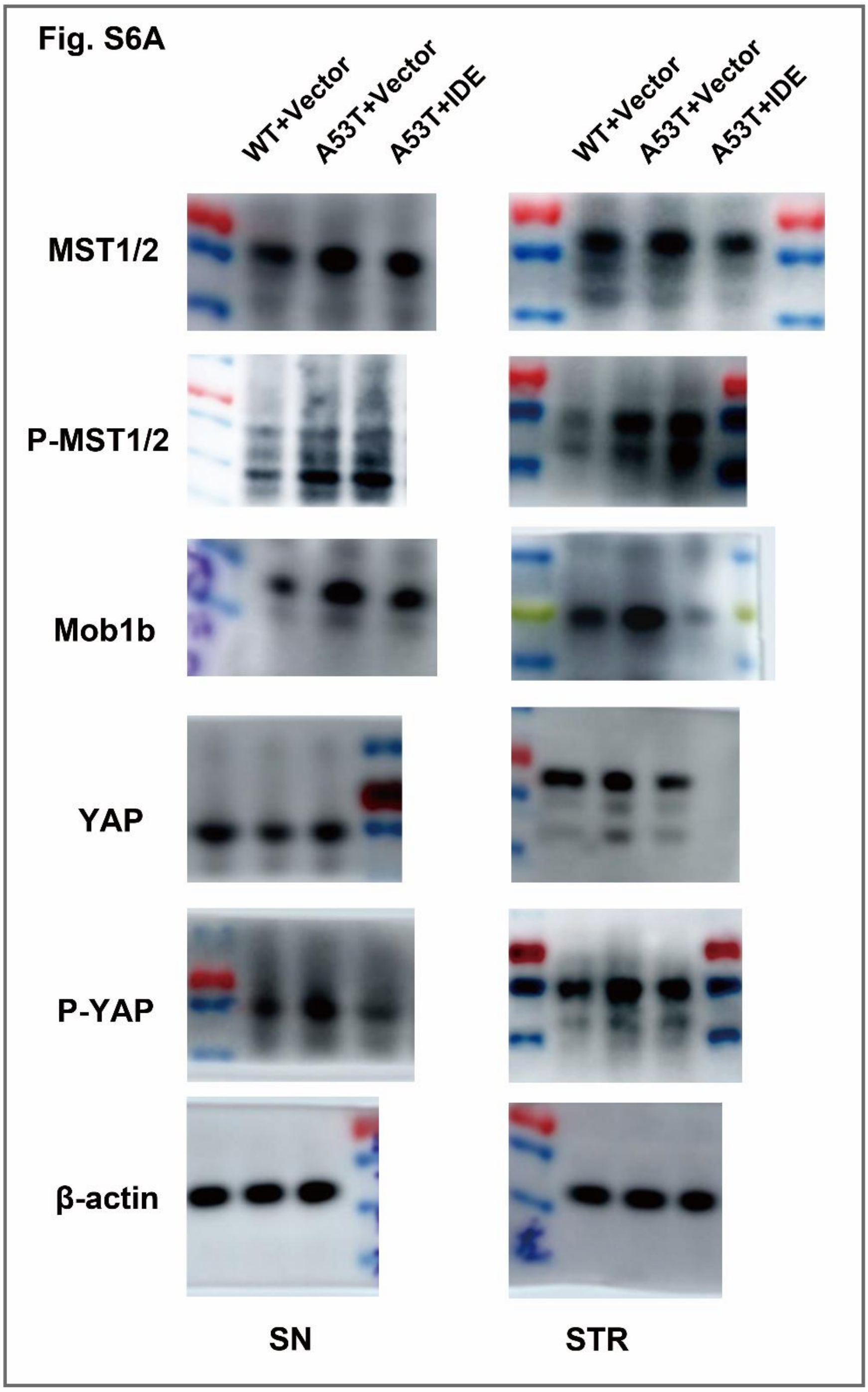
Fig. S6A
WT+Vector
A53T+Vector
A53T+IDE
WT+Vector
A53T+Vector
A53T+IDE
MST1/2
P-MST1/2
Mob1b
YAP
P-YAP
β-actin
SN
STR

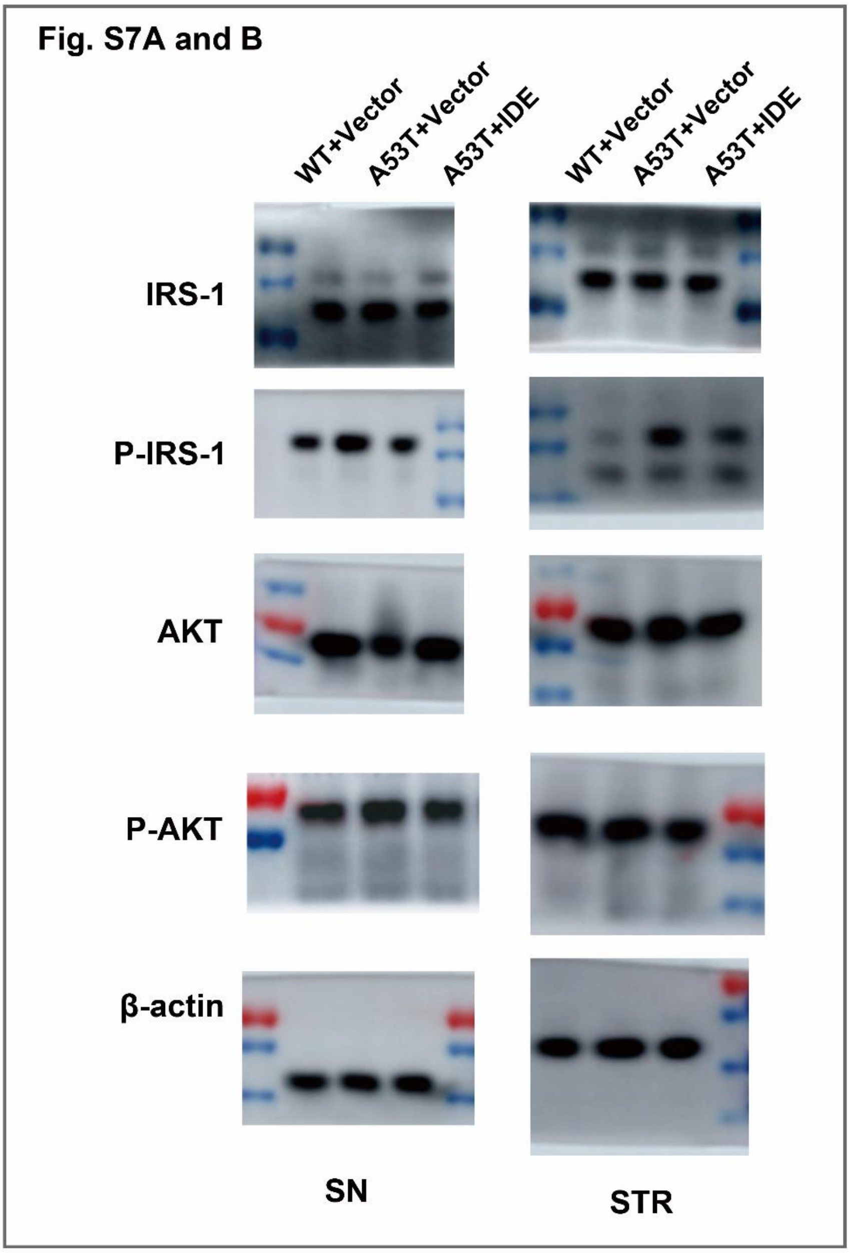
Fig. S7A and B
WT+Vector
A53T+Vector
A53T+IDE
WT+Vector
A53T+Vector
A53T+IDE
IRS-1
P-IRS-1
AKT
P-AKT
β-actin
SN
STR

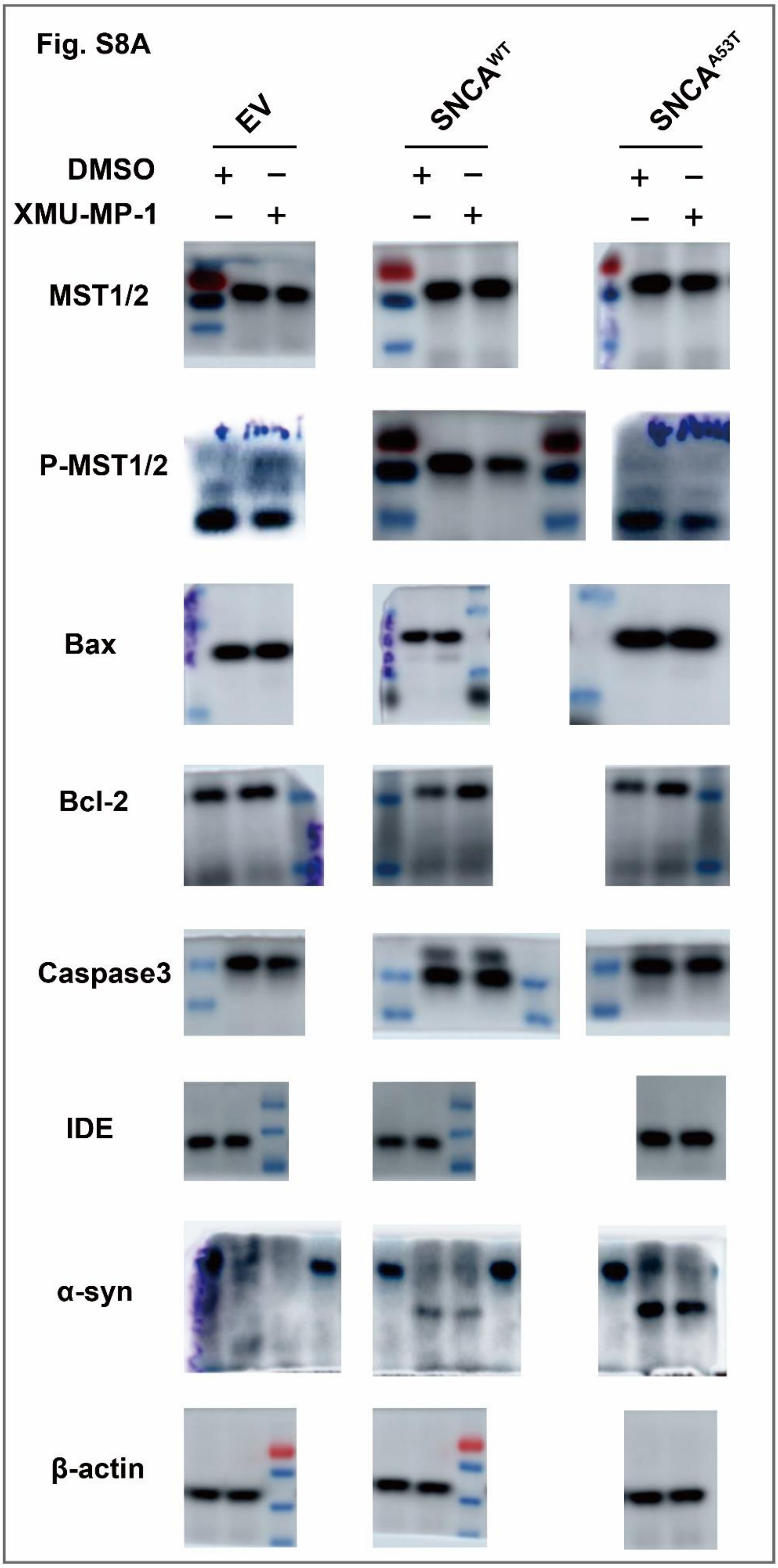
Fig. S8A
EV
SNCA^WT
SNCA^A53T
DMSO
XMU-MP-1
+ −
− +
MST1/2
P-MST1/2
Bax
Bcl-2
Caspase3
IDE
α-syn
β-actin
